# The cumene/O_2_ system: a very simple tool for the radical chain oxidation of some functional groups†
†All the work presented here was conducted in Kaiserslautern.
‡
‡Electronic supplementary information (ESI) available: Experimental procedures and analytical data. See DOI: 10.1039/c7nj01666b


**DOI:** 10.1039/c7nj01666b

**Published:** 2017-06-30

**Authors:** A. Malekafzali, K. Malinovska, F. W. Patureau

**Affiliations:** a FB Chemie , Technische Universiät Kaiserslautern , Erwin-Schrödinger Str. 52 , 67663 Kaiserslautern , Germany . Email: patureau@chemie.uni-kl.de ; http://www.chemie.uni-kl.de/patureau; b Chemistry Department , Tarbiat Modares University , Jalale-Ale-Ahmad Highway , 14117-13116 Tehran , Iran

## Abstract

Due to the relative stability of the cumyl radical, cumenes and α-methyl-styrenes are ideally structured to directly harvest the oxidizing reactivity of O_2_ and initiate radical chain reactions in catalyst-free conditions. In the absence of additional substrates, these processes can lead to acetophenones. In the presence of substrates, the cumene oxidation process can be intercepted in various chain reactions, affording very simple protocols for functional group oxidation.

## Abstract 2

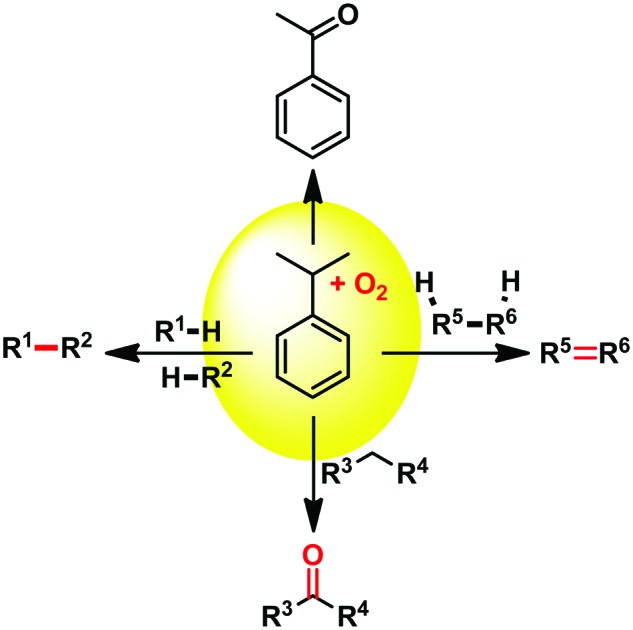
Just boil it in cumene! A general metal-free oxidation method is described.

Phenones are traditionally prepared by ozonolysis (C=C bond cleaving oxidation of styrenes with O_3_),^1^ metal catalysed oxidation of styrenes with O_2_, such as in the Wacker process,^2^ or simply by oxidation of their alkyl benzene precursors, to name only a few methods.^3^ The chemistry of phenones, their preparation but also their very rich and versatile reactivity has been at the heart of organic synthesis for well over a century. Recently, Jianliang Xiao reported a C=C bond cleaving oxidation of styrenes towards the corresponding phenones while using an iron(iii)-triflate catalyst bearing a tridentate amine ligand, with O_2_ as the terminal oxidant (Scheme 1).^4^ More recently, Xiao Wang reported an analogous protocol utilizing an aromatic disulfide organocatalyst under white LED, also under 1 atm of O_2_.^5^ The latter methods afford the corresponding phenones in good yields at mild temperatures (as low as 25 °C, Scheme 1). However to what extend exactly does one need a catalyst for this reaction to occur? While the activation of molecular O_2_ can be troublesome in mild reaction conditions without a catalyst, some early work conducted by Hayashi in 2002 did document the neat transformation of α-methyl-styrenes into acetophenones with O_2_ as the sole reagent.^6^

**Scheme 1:**
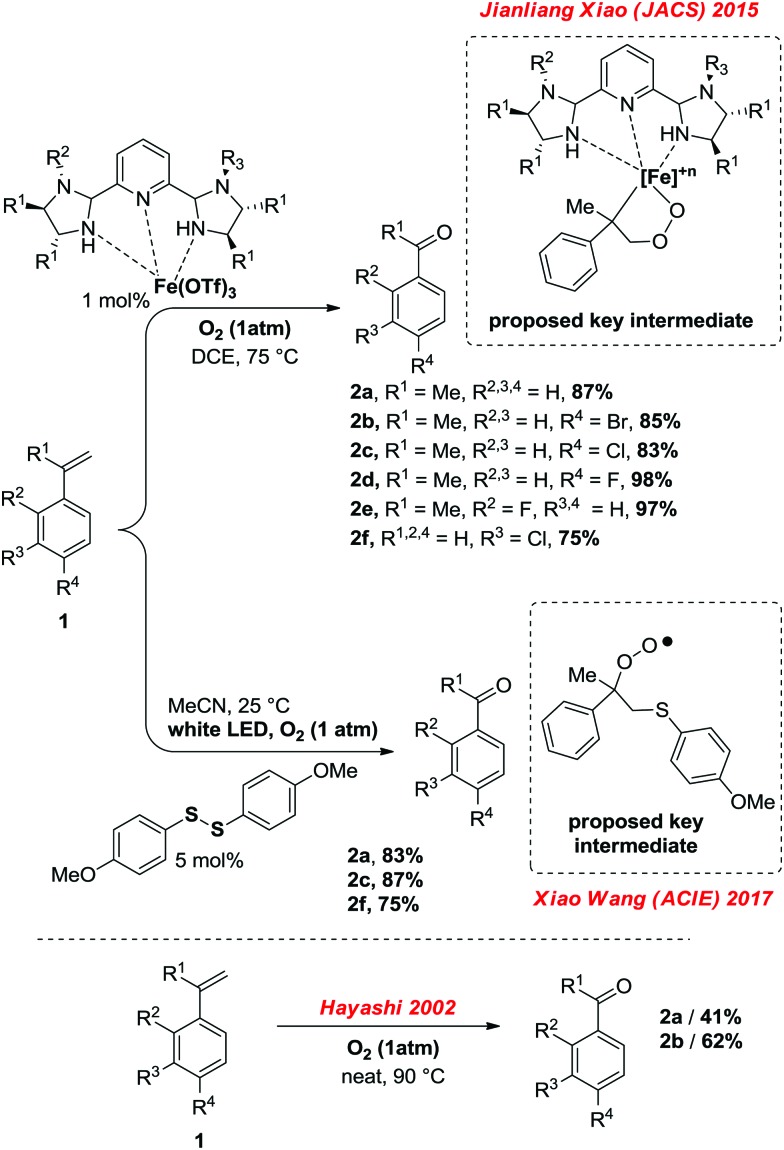
Oxygenolysis of (α-methyl)-styrenes according to recent reports of Xiao (top)^4^ Wang (middle),^5^ selected examples, in comparison to the catalyst-free method of Hayashi (beneath).^6^

Beyond Hayashi's catalyst free method for α-methyl-styrenes,^6^ it seemed clear that based on the known chemistry of cumyl-hydroperoxide, pure cumene should also be a competent substrate in this reaction. This assumption is moreover supported by the decades-old decomposition of cumyl-hydroperoxide, which is known to yield acetophenone.^7^ It thus occurred to us that cumene,^8^ a precursor to α-methyl-styrene, would probably display a similar reactivity than Hayashi's oxygenolysis with O_2_, in completely additive free conditions.^9^ We thus conducted an initial reactivity and mechanistic survey, summarized in Table 1.

**Table 1:** Initial screening overview in 85 mL sealed reactors, with 1 atm of O_2_ (3.5 mmol ± 0.2 mmol), yields in (mmol) determined by ^1^H NMR integration, internal standard: 1,1,2,2-tetrachloroethane

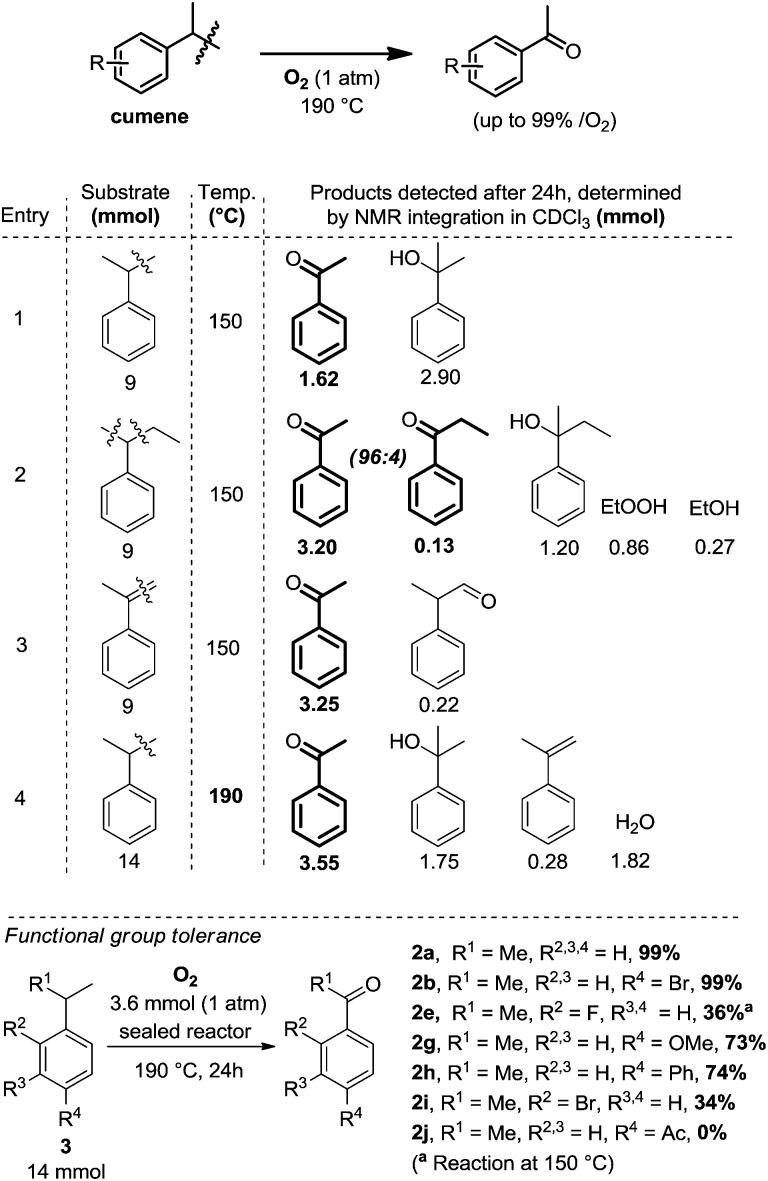

The following reaction conditions were eventually selected: cumene, 14 mmol, is placed in an 85 mL glass reactor, which is then flushed with O_2_ for approximatively 1 minute, and then sealed and heated up for 24 h at 190 °C. When cumene is submitted to those conditions, one molecule of O_2_ produces approximatively one molecule of acetophenone. The reaction is still operational at 150 °C but conversion is then lower (1.62 mmol, Table 1, entry 1). By increasing reaction temperature, dehydration of the dimethyl-phenyl-carbinol byproduct increases also, yielding the corresponding α-methyl styrene which can then re-engage itself in an oxidation event with O_2_ to produce the desired acetophenone. As opposed to the Xiao^4^ and Wang^5^ systems, in which the position of the olefin starting material is already defined in the substrate, starting directly from unsymmetrical alkyl-benzene precursors can offer competing C–C bond cleaving pathways. Interestingly, utilizing 2-phenyl-butane instead of cumene is more effective at 150 °C than cumene, with a yield of acetophenone of 3.20 mmol (entry 2). Notably, C–C bond cleavage occurs with a very large preference on the side of the longer alkyl chain, as the acetophenone product largely exceeds the propiophenone product by a ratio of 96 : 4 (entry 2). This suggests the intermediacy of the corresponding styrene as a significant intermediate, similarly to the Xiao,^4^ Wang^5^ and Hayashi^6^ systems, the internal alkene being largely favored over the external one in the case of an *in situ* dehydrogenative event. This is confirmed by entry 3, in which neat α-methylstyrene is also converted to acetophenone (3.25 mmol).^9^ We also tried to run the reaction under air at cumene's boiling point. Only 2.6% of the cumene converted to acetophenone in those open-flask conditions. With those initial conditions in hand, we then evaluated the effects of some simple electron donating and withdrawing substitution patterns (Table 1). We found that strongly withdrawing groups (R^4^ = Ac, **2j**), make the cumenes unreactive, likely by preventing the first oxidation step: the cumyl H-atom abstraction. For halides, methoxy, and phenyl functional groups, the tolerance is generally acceptable. A mechanism is proposed in Scheme 2 consisting in a stepwise radical^10^ dehydrogenation, producing α-methyl-styrene which would then be further oxidized with O_2_ in a Hayashi-like mechanism.^6^ Alternatively, a classical cumyl-hydroperoxide route can be considered, as documented notably by Di Somma,^7^ which is also reasonable in view of some of the byproducts observed in Table 1. In any case, 2 mmol of 2,4,6-tri-*tert*-butylphenol added to the reactor as a radical inhibitor suppresses acetophenone formation (Scheme 2). This result thus confirms the radical chain character of this transformation. Because cumene is so readily oxidized by O_2_, it may potentially be a good solvent to carry out simple functional group oxidation and/or oxidative coupling reactions through radical chain oxidations. In two recent cross-dehydrogenative coupling methods, the cumene/O_2_ couple was utilized as an enabling (re-)oxidizing strategy (Scheme 3). One method relies on a Ru/Cu catalysed C–H/N–H bond activation system,^11^ while the other method is entirely additive-free.^8^ The cumene/O_2_ system notably enables the direct and additive-free cross dehydrogenative C–N coupling of phenols with phenothiazines in excellent yields, a reaction which we previously reported.^8^ A similar system also allows the C–S cross dehydrogenative coupling between phenols and thiophenols, although with moderate yields (product **8a**, 44%).^12^ We thus wondered whether the cumene/O_2_ system would also be competent for simple functional group oxidation. We therefore considered the oxidation of a series of organic substrates under those conditions. For example, benzhydrol **9** yields benzophenone (entry **14**, 97% isolated yield). Diphenylmethane **10** also yields benzophenone (entry **15**, 88%), while xanthene (**11**) and fluorene (**12**) produce xanthone **16** and fluorenone **17** with 93% and 74% yield respectively. Finally 1,2-diphenylhydrazine (**13**) yields azobenzene **18** (93%). All were efficiently transformed while simply being heated up in cumene under 1 atm of O_2_ at 150 °C, without any further additive. These trivial oxidation processes are among the most studied reactions in the literature.^13^–^16^ However the vast majority of these oxidative methods rely on metal additives, sometimes also on externally added oxidants, which arguably makes our method an interesting alternative.

**Scheme 2:**
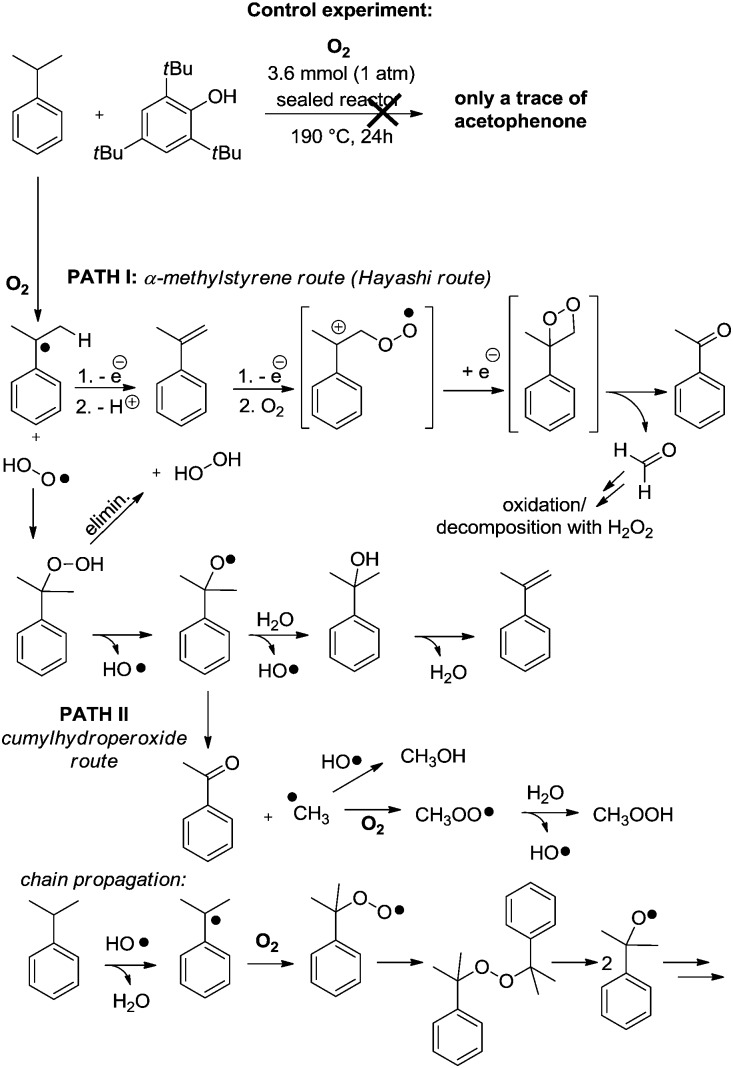
Possible chain propagation mechanisms taking into account the observed by-products of Table 1, PATH I through α-methyl-styrene, notably proposed by Hayashi,^6^ and PATH II through the cumyl-hydroperoxide route, notably documented by Di Somma.^7^

**Scheme 3:**
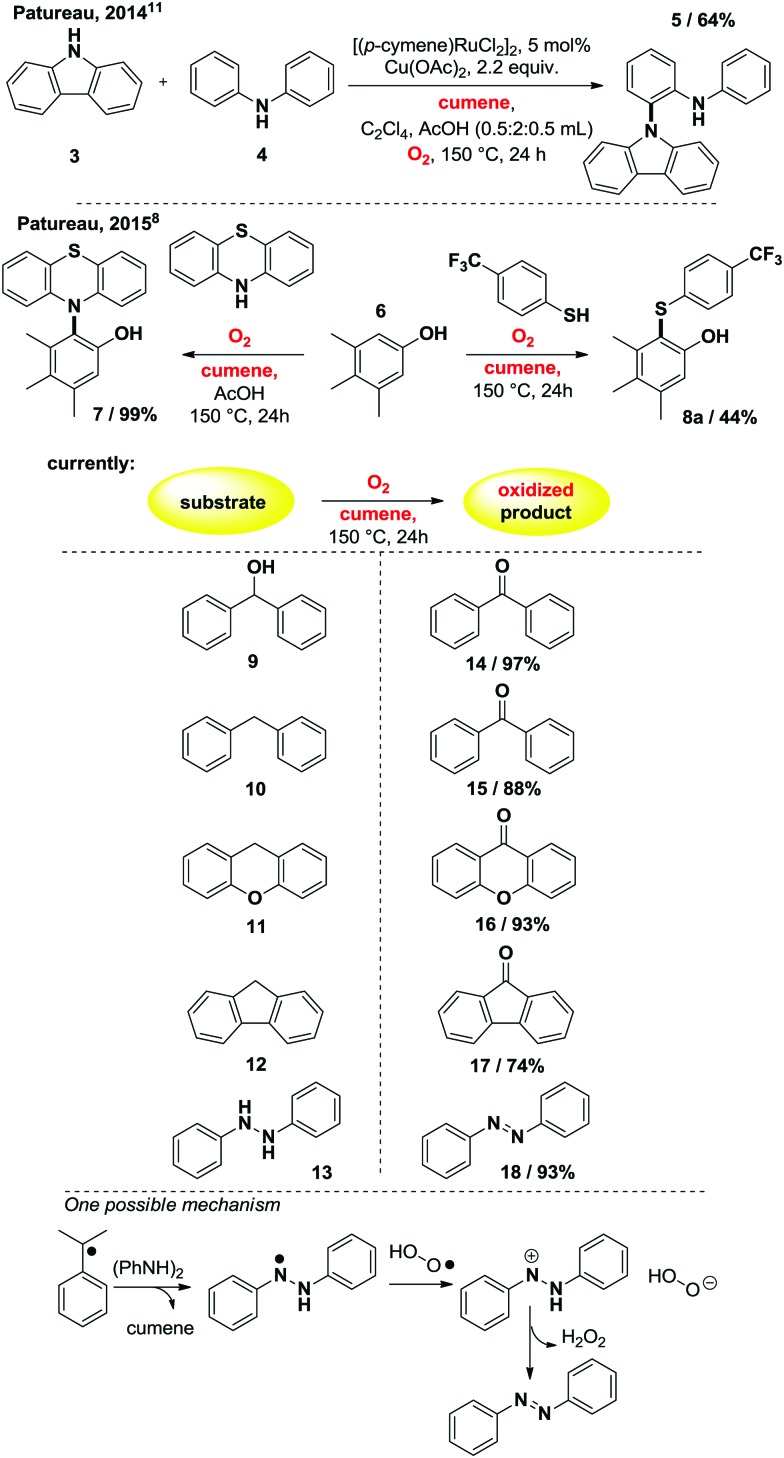
Cumene/O_2_ as a simple oxidizing method, isolated yields.

In conclusion, the oxidative C–C bond cleaving oxidation of cumenes or α-methyl-styrenes with O_2_ towards the corresponding phenones can proceed efficiently without a catalyst. These typically require significantly higher temperatures however than under catalytic conditions.^17^ Importantly, the O_2_ mediated oxidation of cumene can be intercepted in the frame of cross dehydrogenative couplings, or the simple oxidation of diverse functional groups. Clearly, cumene and its congeners are well suited for the organic activation of O_2_ towards oxidative applications.

## Supplementary Material

Supplementary information
