# Supplementary material for: The cumene/O2 system: a very simple tool for the radical chain oxidation of some functional groups
Source: New J Chem. 2017 Jun 30;41(15):6981–5. doi: 10.1039/c7nj01666b (PMC6208464; doi:10.1039/c7nj01666b)
Supplement: Supplementary file 1 [file NJ-041-C7NJ01666B-s001.pdf]

Electronic Supplementary Material (ESI) for New Journal of Chemistry.
This journal is © The Royal Society of Chemistry and the Centre National de la Recherche Scientifique 2017

The cumene/O_2 system: a very simple tool for the radical chain oxidation of some functional groups

Alaleh Malekafzali, Karina Malinovska, and Frederic W. Patureau*

Technische Universität Kaiserslautern, FB Chemie, Erwin Schrödinger Strasse 52, Kaiserslautern, Germany

patureau@chemie.uni-kl.de

Supporting Information, NJC 2017

Contents :

1) **General Information, p. S2**
2) **Methods, p. S2**
3) **Product characterization, p. S3**
4) **Spectra, p. S8**

1) General Information

All reactions were carried out in dried reaction vessels with sealed Teflon screw caps under oxygen. NMR spectra were obtained on Bruker AMX 400 using $CDCl_3$ as solvent, with proton and carbon resonances at 400 and 101 MHz, respectively. Coupling constants (J) are quoted in Hz. Flash chromatography was performed on silica gel (40-63 mesh) by standard technique. Substrates were purchased either from Sigma Aldrich, Alfa Aesar, TCI, or ABCR. HR-MS data were determined on a WATERS GCT-Premier™ mass spectrometer.

2) Methods

Standard conditions for the oxidation of cumenes with O_2: 85 ml reactor equipped with Teflon screw cap was charged with cumene derivatives (14 mmol). The reactor was then flushed with oxygen atmosphere (1-2 min.), then sealed (tightly) and exposed to 190°C for 24 h (magnetic stirring set to approx. 500 turns/min). The reactor was then cooled to room temperature. $C_2H_2Cl_4$ (0.5 mmol) was added to the mixture and the mixture has been measured by NMR spectroscopy.

Control experiment: 2 mmol of 2,4,6-tritertbutylphenol is added to the otherwise standard reactions described above. Only a trace of benzophenone was observed.

Standard conditions for the cross dehydrogenative coupling of phenols with thiophenols under cumene/O_2: phenol (1 mmol), thiophenol (0.5 mmol) and cumene (1 ml) are united under air in a 85 ml reactor equipped with Teflon screw cap. The reactor is then flushed with oxygen atmosphere (1-2 min.), then sealed (tightly) and exposed to 150°C for 24 h. (magnetic stirring set to approx. 500 turns/min). The reactor is then cooled to room temperature. The solvent from the mixture was evaporated under reduced pressure to leave a residue that was purified by column chromatography (SiO_2 gel).

Standard conditions for the oxidation of 9, 10, 11, 12 and 13 under cumene/O_2: The substrate (1 mmol) and cumene (1.2 ml) are united under air in an 85 ml reactor equipped with Teflon screw cap. The reactor is then flushed with oxygen atmosphere (1-2 min.), then sealed (tightly) and exposed to 150°C for 24 h. (magnetic stirring set to approx. 500 turns/min). The reactor is then cooled to room temperature. The solvent from the mixture was evaporated under reduced pressure to leave a residue that was purified by column chromatography (SiO_2 gel) hexane/ethyl acetate (15:1).

Note: for the cross dehydrogenative coupling of phenols with phenothiazines, please refer to this paper: M.-L. Louillat-Habermeyer, R. Jin & F. W. Patureau, *Angew. Chem. Int. Ed.* **2015**, *54*, 4102.

3) Product Characterization

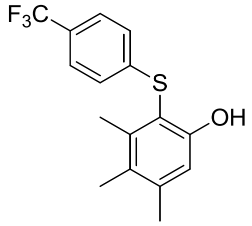

Chemical Formula: $C_{16}H_{15}F_3OS$
Exact Mass: 312,0796
Molecular Weight: 312,3499
m/z: 312.0796 (100.0%), 313.0829 (17.3%), 314.0754 (4.5%), 314.0863 (1.4%)
Elemental Analysis: C, 61.52; H, 4.84; F, 18.25; O, 5.12; S, 10.27

8a. From 4-trifluoromethylthiophenol and 3,4,5-trimethylphenol. The concentrated reaction mixture is purified by SiO_2 gel column chromatography hexane/DCM (5:1). Isolated yield: 44% (white powder).

^{1}H NMR (400 MHz, $CDCl_3$)δ (ppm): 2.09 (3H, s, Me), 2.24 (3H, s, Me), 2.28 (3H, s, Me), 6.37 (1H, s, OH), 6.75 (1H, s, CH), 6.98 (2H, d, ^3J = 8.4 Hz, CH), 7.36 (2H, d, ^3J = 8.4 Hz, CH).

^{13}C {^{1}H} NMR (101 MHz, $CDCl_3$) δ (ppm): 15.97 (s, Me), 18.23 (s, Me), 21.25 (s, Me), 111.55(s, C), 114.33 (s, CH), 124.07 (q, ^1J = 270.6 Hz, CF_3), 125.66 (s, CH), 125.96 (q, ^3J = 3.7 Hz, CH), 127.75 (q, ^2J = 32.6 Hz, C), 128.25 (s, C), 141.25 (s, C), 141.42 (s, C), 141.62 (s, C), 155.17(s, C).

^{19}F NMR (376.5 MHz, $CDCl_3$) δ (ppm): -62.43 (s, CF_3).

IR (neat, cm^{-1}): ν: 3408, 2925, 1919, 1603, 1566, 1496, 1458, 1401, 1320, 1291, 1223, 1201, 1164, 1152, 1127, 1107, 1087, 1061, 1040, 1011, 952, 851, 830, 779.

EI-HRMS: mass spectrometry: m/z calc. 312.0796 [$C_{16}H_{15}F_3OS$] $^{\bullet+}$, measured 312.0766.

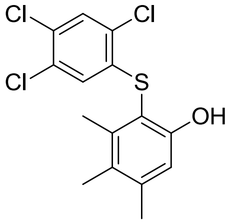

Chemical Formula: $C_{15}H_{13}Cl_3OS$
Exact Mass: 345,9753
Molecular Weight: 347,6871
m/z: 345.9753 (100.0%), 347.9723 (95.9%), 349.9694 (30.6%), 346.9786 (16.2%), 348.9757 (15.6%), 350.9727 (5.0%), 347.9711 (4.5%), 349.9681 (4.3%), 351.9664 (3.3%), 351.9652 (1.4%), 347.9820 (1.2%), 349.9790 (1.2%)
Elemental Analysis: C, 51.82; H, 3.77; Cl, 30.59; O, 4.60; S, 9.22

8b. From 2,4,5-trichlorothiophenol and 3,4,5-trimethylphenol. The concentrated reaction mixture is purified by SiO_2 gel column chromatography hexane/DCM (5:1). Isolated yield: 24% (pale yellow powder).

^{1}H NMR (400 MHz, $CDCl_3$) δ (ppm): 2.11 (3H, s, Me), 2.26 (3H, s, Me), 2.28 (3H, s, Me), 6.21 (1H, s, OH), 6.39 (1H, s, CH), 6.76 (1H, s, CH), 7.39 (1H, s, CH).

^{13}C {^{1}H} NMR (101 MHz, $CDCl_3$) δ (ppm): 16.04 (s, Me), 18.20 (s, Me), 21.31 (s, Me), 110.35 (s, C), 114.74 (s, CH), 126.55 (s, CH), 128.67 (s, C), 129.79 (s, C), 129.81 (s, C), 130.74 (s, CH), 132.04 (s, C), 135.79 (s, C), 141.60 (s, C), 142.07 (s, C), 155.17 (s, C).

IR (neat, cm^{-1}): ν: 3429, 3409, 3070, 2921, 2853, 1737, 1598, 1563, 1460, 1430, 1374, 1322, 1295, 1256, 1225, 1204, 1149, 1107, 1054, 1038, 1000, 949, 926, 879, 863, 852, 782.

EI-HRMS: mass spectrometry: m/z calc. 345.9753 and 347.9723 $[C_{15}H_{13}Cl_3OS]^{\bullet+}$, measured 345.9746 and 347.9726 respectively.

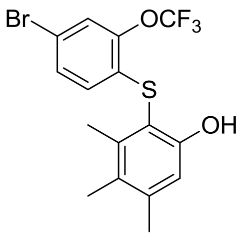

Chemical Formula: $C_{16}H_{14}BrF_3O_2S$
Exact Mass: 405,9850
Molecular Weight: 407,2454
m/z: 405.9850 (100.0%), 407.9830 (97.3%), 406.9884 (17.3%), 408.9863 (16.8%), 407.9808 (4.5%), 409.9787 (4.4%), 407.9917 (1.4%), 409.9897 (1.4%)
Elemental Analysis: C, 47.19; H, 3.47; Br, 19.62; F, 14.00; O, 7.86; S, 7.87

8c. From 4-bromo-2-(trifluoromethoxy)thiophenol and 3,4,5-trimethylphenol. The concentrated reaction mixture is purified by SiO_2 gel column chromatography hexane/DCM (5:1). Isolated yield: 24% (pale yellow powder).

^{1}H NMR (400 MHz, $CDCl_3$) δ (ppm): 2.09 (3H, s, Me), 2.24 (3H, s, Me), 2.26 (3H, s, Me), 6.28 (1H, s, OH), 6.32 (1H, d, ^3J= 8.5 Hz, CH), 6.74 (1H, s, CH), 7.10 (1H, dd, ^3J = 8.5 Hz, J = 2.0 Hz, CH), 7.33 (1H, quint, J = 1.5 Hz, CH).

^{13}C {^{1}H} NMR (101 MHz, $CDCl_3$) δ (ppm): 15.97 (s, Me), 18.09 (s, Me), 21.24 (s, Me), 110.04 (s, C), 114.49 (s, CH), 118.85 (s, C), 120.54 (q, ^1J = 259.7 Hz, CF_3), 124.31 (s, CH), 127.74 (s, CH), 128.43 (s, C), 129.65 (s, C), 130.67 (s, CH), 141.64 (s, C), 141.85 (s, C), 145.89 (s, C), 155.41 (s, C).

^{19}F NMR (376.5 MHz, $CDCl_3$) δ (ppm): -57.51 (s, CF_3).

IR (neat, cm^{-1}): ν: 3402, 2925, 2857, 1595, 1561, 1465, 1382, 1316, 1245, 1213, 1197, 1168, 1149, 1087, 1054, 1037, 938, 866, 852, 841, 817, 799.

EI-HRMS: mass spectrometry: m/z calc. 405.9850 and 407.9830 $[C_{16}H_{14}BrF_3O_2S]^{\bullet+}$, measured 405.9861 and 407.9842 respectively.

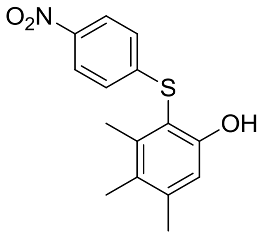

Chemical Formula: $C_{15}H_{15}NO_3S$
Exact Mass: 289,0773
Molecular Weight: 289,3495
m/z: 289.0773 (100.0%), 290.0806 (16.2%), 291.0731 (4.5%), 291.0840 (1.2%)
Elemental Analysis: C, 62.26; H, 5.23; N, 4.84; O, 16.59; S, 11.08

8d. From 4-nitrothiophenol and 3,4,5-trimethylphenol. The concentrated reaction mixture is purified by SiO_2 gel column chromatography hexane/EtOAc (20:1). Isolated yield: 21% (yellow powder).

^1H NMR (400 MHz, $CDCl_3$) δ (ppm): 2.11 (3H, s, Me), 2.26 (3H, s, Me), 2.28 (3H, s, Me), 6.22 (1H, s, OH), 6.77 (1H, s, CH), 6.99 (2H, AA'part, 6.9765, 6.9831, 6.9878, 7.007, 7.0054, 7.0120), 7.99 (2H, BB' part, 7.9719, 7.9782, 7.9832, 7.9957, 8.0008, 8.0070).

^{13}C {^1H} NMR (101 MHz, $CDCl_3$) δ (ppm): 16.00 (s, Me), 18.21 (s, Me), 21.28 (s, Me), 110.72 (s, C), 114.62 (s, CH), 124.26 (s, CH), 125.58 (s, CH), 128.56 (s, C), 141.51 (s, C), 141.93 (s, C), 145.66 (s, C), 145.94 (s, C), 155.12 (s, C).

IR (neat, cm^{-1}): ν: 3432, 2921, 2853, 1593, 1572, 1501, 1475, 1425, 1377, 1334, 1319, 1288, 1222, 1198, 1152, 1110, 1083, 1045, 1008, 957, 926, 854, 838, 781, 740, 680.

EI-HRMS: mass spectrometry: m/z calc. 289.0773 $[C_{15}H_{15}NO_3S]^{\bullet+}$, measured 289.0775.

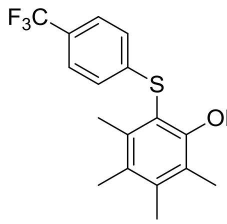

Chemical Formula: $C_{17}H_{17}F_3OS$
Exact Mass: 326,0952
Molecular Weight: 326,3765
m/z: 326.0952 (100.0%), 327.0986 (18.4%), 328.0910 (4.5%), 328.1019 (1.6%)
Elemental Analysis: C, 62.56; H, 5.25; F, 17.46; O, 4.90; S, 9.82

8e. From 4-trifluorothiophenol and 2,3,4,5-tetramethylphenol[1]. The concentrated reaction mixture is purified by SiO_2 gel column chromatography hexane/DCM (10:1). Isolated yield: 23% (white powder).

^{1}H NMR (400 MHz, $CDCl_3$) δ (ppm): 2.14 (3H, s, Me), 2.18 (3H, s, Me), 2.20 (3H, s, Me), 2.29 (3H, s, Me), 6.61 (1H, s, OH), 6.98 (2H, d, ^3J = 8.3 Hz, CH), 7.36 (2H, d, ^3J = 8.3 Hz, CH).

^{13}C {^{1}H} NMR (101 MHz, $CDCl_3$) δ (ppm): 13.14 (s, Me), 16.74 (s, Me), 16.80 (s, Me), 18.45 (s, Me), 111.12 (s, C), 120.62 (s, C), 124.10 (q, ^1J = 272.7 Hz, CF_3), 125.66 (s, CH), 125.92 (q, ^3J = 3.6 Hz, CH), 127.68 (q, ^2J = 32.8 Hz, C), 127.79 (s, C), 138.24 (s, C), 139.95 (s, C), 141.36 (s, C), 153.34 (s, C).

^{19}F NMR (376.5 MHz, $CDCl_3$) δ (ppm): -62.41 (s, CF_3).

IR (neat, cm^{-1}): ν: 3424, 2925, 1605, 1568, 1497, 1449, 1402, 1325, 1239, 1215, 1164, 1124, 1108, 1086, 1063, 1014, 829, 767, 750.

EI-HRMS: mass spectrometry: m/z calc. 326.0952 $[C_{17}H_{17}F_3OS]^{\bullet+}$, measured 326.0956.

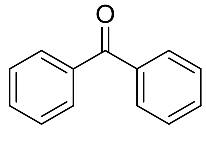

Chemical Formula: $C_{13}H_{10}O$
Exact Mass: 182,0732
Molecular Weight: 182,2179
m/z: 182.0732 (100.0%), 183.0765 (14.1%)
Elemental Analysis: C, 85.69; H, 5.53; O, 8.78

14/15. From Benzhydrol. Isolated yield: 97% . From Diphenylmethane. Isolated yield: 88% .

^{1}H NMR (400 MHz, $CDCl_3$) δ (ppm): 7.36 (4H, t, ^3J = 7.3 Hz, CH), 7.47 (2H, t, ^3J = 7.3 Hz, CH), 7.69 (4H, d, ^3J = 7.1 Hz, CH).

^{13}C {^{1}H} NMR (101 MHz, $CDCl_3$) δ (ppm): 128.36 (CH), 130.08 (CH), 132.52 (CH), 137.69 (C), 196.73 (CO).

IR (neat, cm^{-1}): ν: 1657, 1598, 1580, 1494, 1447, 1318, 1275, 1176, 1150, 1074, 1028, 999, 974, 941, 918, 883, 848, 810, 763, 696, 670.

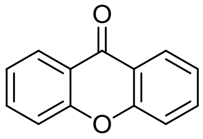

Chemical Formula: $C_{13}H_8O_2$
Exact Mass: 196,0524
Molecular Weight: 196,2014
m/z: 196.0524 (100.0%), 197.0558 (14.1%)
Elemental Analysis: C, 79.58; H, 4.11; O, 16.31

16. From Xanthene. Isolated yield: 93% .

^{1}H NMR (400 MHz, $CDCl_3$) δ (ppm): 7.30 (2H, dt, ^{3}J = 7.1 Hz, J = 1.4 Hz, CH), 7.49 (2H, dt, ^{3}J = 7.4 Hz, J = 1.1 Hz, CH), 7.52 (2H, td, ^{3}J = 7.4 Hz, J = 1.3 Hz, CH), 7.66 (2H, td, ^{3}J = 7.4 Hz, J = 0.9 Hz, CH).

^{13}C {^{1}H} NMR (101 MHz, $CDCl_3$) δ (ppm): 120.31 (CH), 124.32 (CH), 129.13 (CH), 134.14 (C), 134.70 (CH), 144.44 (C), 193.95 (CO).

IR (neat, cm^{-1}): ν: 1653, 1616, 1604, 1567, 1479, 1452, 1345, 1329, 1276, 1239, 1211, 1192, 1143, 1113, 1099, 1031, 1023, 989, 967, 932, 881, 842, 829, 807, 783, 754, 700, 680, 656.

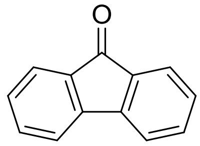

Chemical Formula: $C_{13}H_8O$
Exact Mass: 180,0575
Molecular Weight: 180,2020
m/z: 180.0575 (100.0%), 181.0609 (14.1%)
Elemental Analysis: C, 86.65; H, 4.47; O, 8.88

17. From Fluorene. Isolated yield: 74% .

^{1}H NMR (400 MHz, $CDCl_3$) δ (ppm): 7.29 (2H, dt, ^{3}J = 7.3 Hz, J = 1.3Hz, CH), 7.48 (2H, dt, ^{3}J = 7.4 Hz, J = 1.0 Hz, CH), 7.52 (2H, d, ^{3}J = 7.4 Hz, CH), 7.66 (2H, d, ^{3}J = 7.4 Hz, CH).

^{13}C {^{1}H} NMR (101 MHz, $CDCl_3$) δ (ppm): 120.25 (CH), 124.37 (CH), 129.11 (CH), 134.04 (C), 134.76 (CH), 144.25 (C), 194.03 (CO).

IR (neat, cm^{-1}): ν: 1712, 1610, 1598, 1472, 1450, 1371, 1324, 1296, 1189, 1147, 1095, 1012, 988, 945, 914, 876, 809, 774, 724, 664.

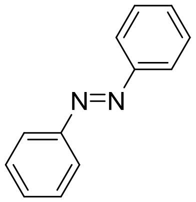

Chemical Formula: $C_{12}H_{10}N_2$
Exact Mass: 182,0844
Molecular Weight: 182,2212
m/z: 182.0844 (100.0%), 183.0878 (13.0%)
Elemental Analysis: C, 79.10; H, 5.53; N, 15.37

18. From Hydrazobenzene. Isolated yield: 93% .

^{1}H NMR (400 MHz, $CDCl_3$) δ (ppm): 7.39 (2H, t, ^{3}J = 7.1 Hz, CH), 7.44 (4H, t, ^{3}J = 6.8 Hz, CH), 7.84 (4H, d, ^{3}J = 7.5 Hz, CH).

^{13}C {^{1}H} NMR (101 MHz, $CDCl_3$) δ (ppm): 122.88 (CH), 129.17 (CH), 130.99 (CH), 152.67 (C).

IR (neat, cm^{-1}): ν: 1582, 1535, 1483, 1453, 1398, 1346, 1300, 1221, 1159, 1150, 1071, 1019, 999, 984, 925, 851, 769, 664.

4) Spectra

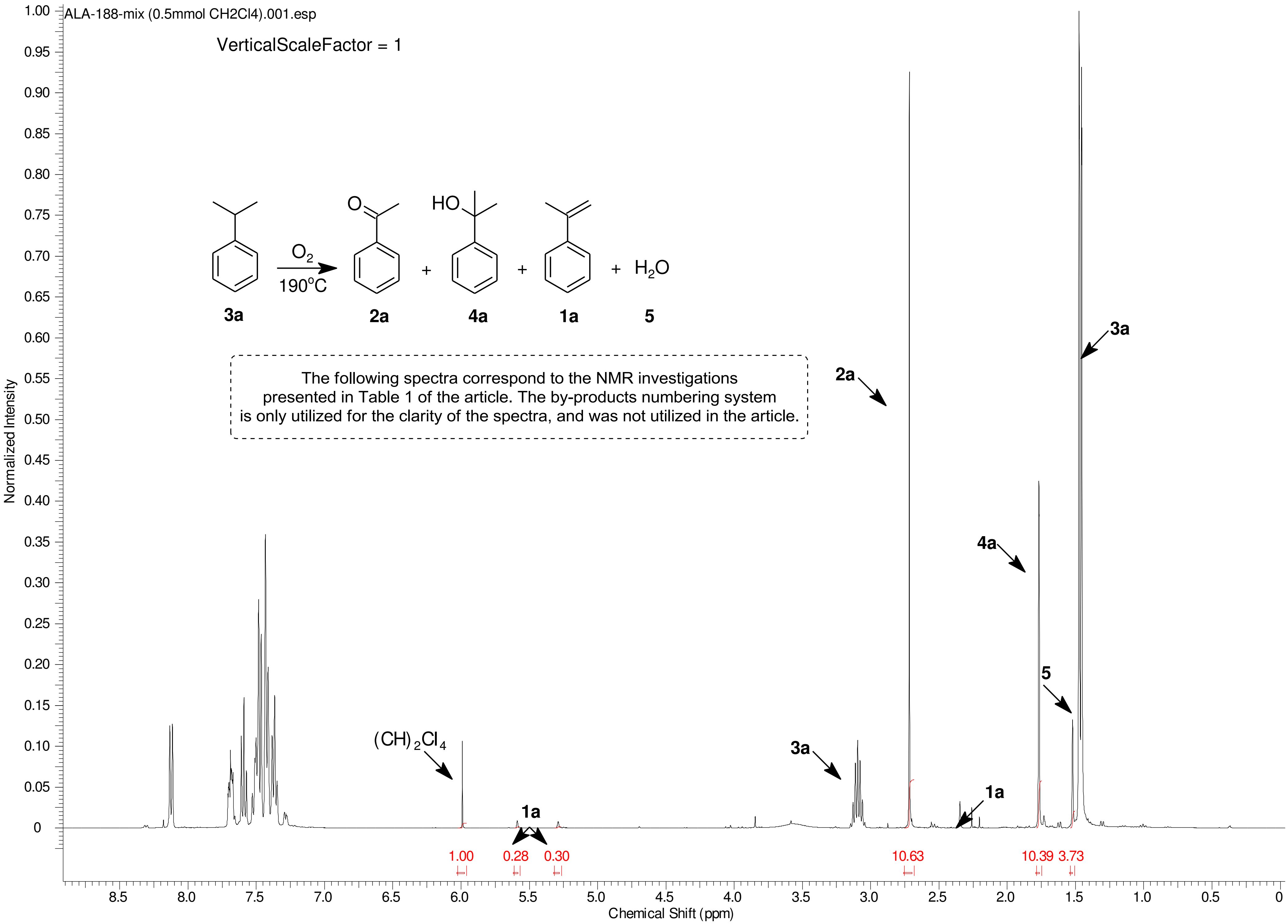
ALA-188-mix (0.5mmol CH2Cl4).001.esp
VerticalScaleFactor = 1
O2
190oC
HO
O
H2O
3a
2a
4a
1a
5
The following spectra correspond to the NMR investigations presented in Table 1 of the article. The by-products numbering system is only utilized for the clarity of the spectra, and was not utilized in the article.
(CH)2Cl4
1.00
0.28
0.30
10.63
10.39
3.73
Normalized Intensity
Chemical Shift (ppm)

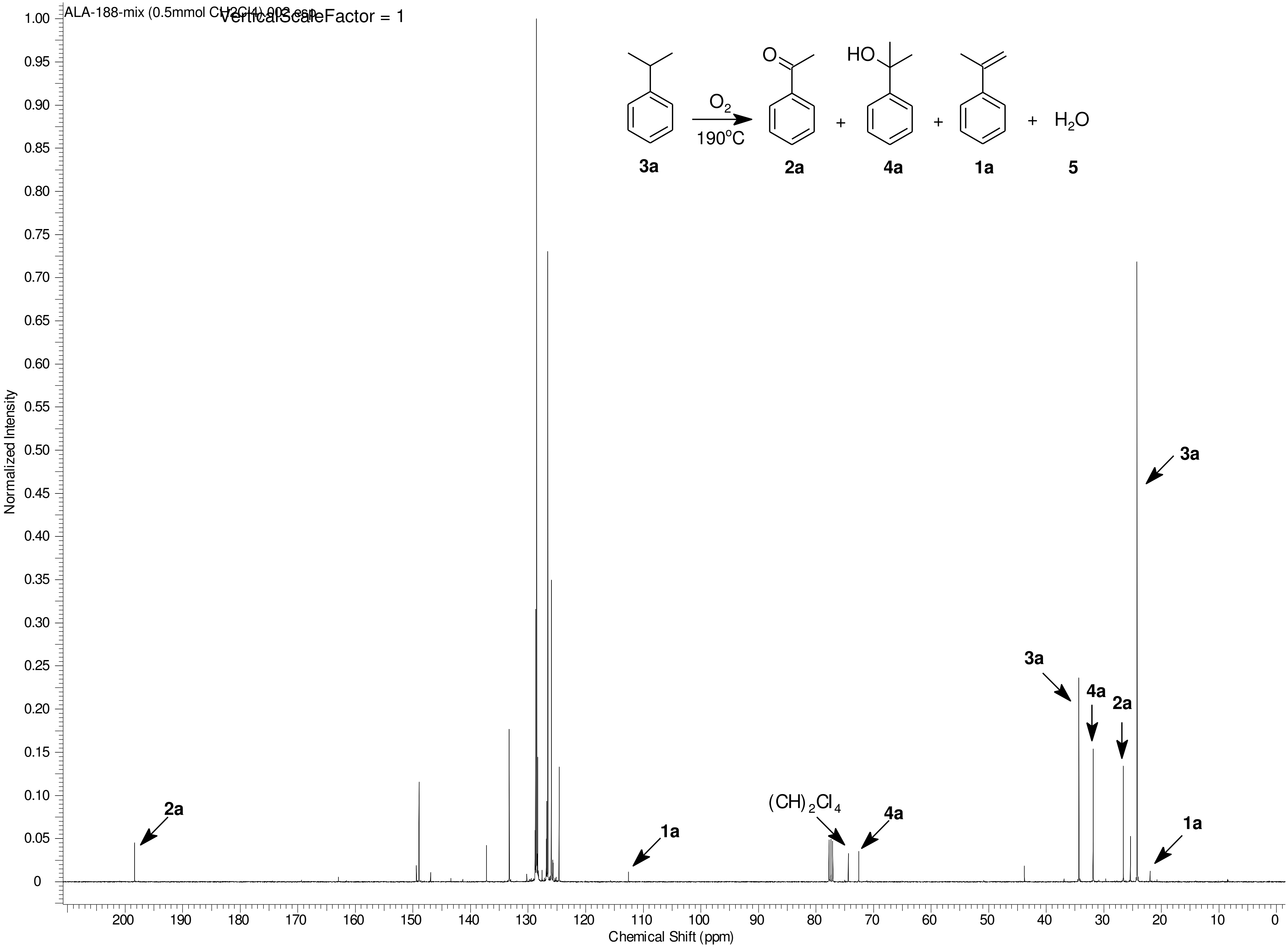
ALA-188-mix (0.5mmol CH2Cl4) 002.esp
VerticalScaleFactor = 1
O2
190°C
3a
2a
4a
1a
5
H2O
HO
O
(CH)2Cl4
Normalized Intensity
Chemical Shift (ppm)

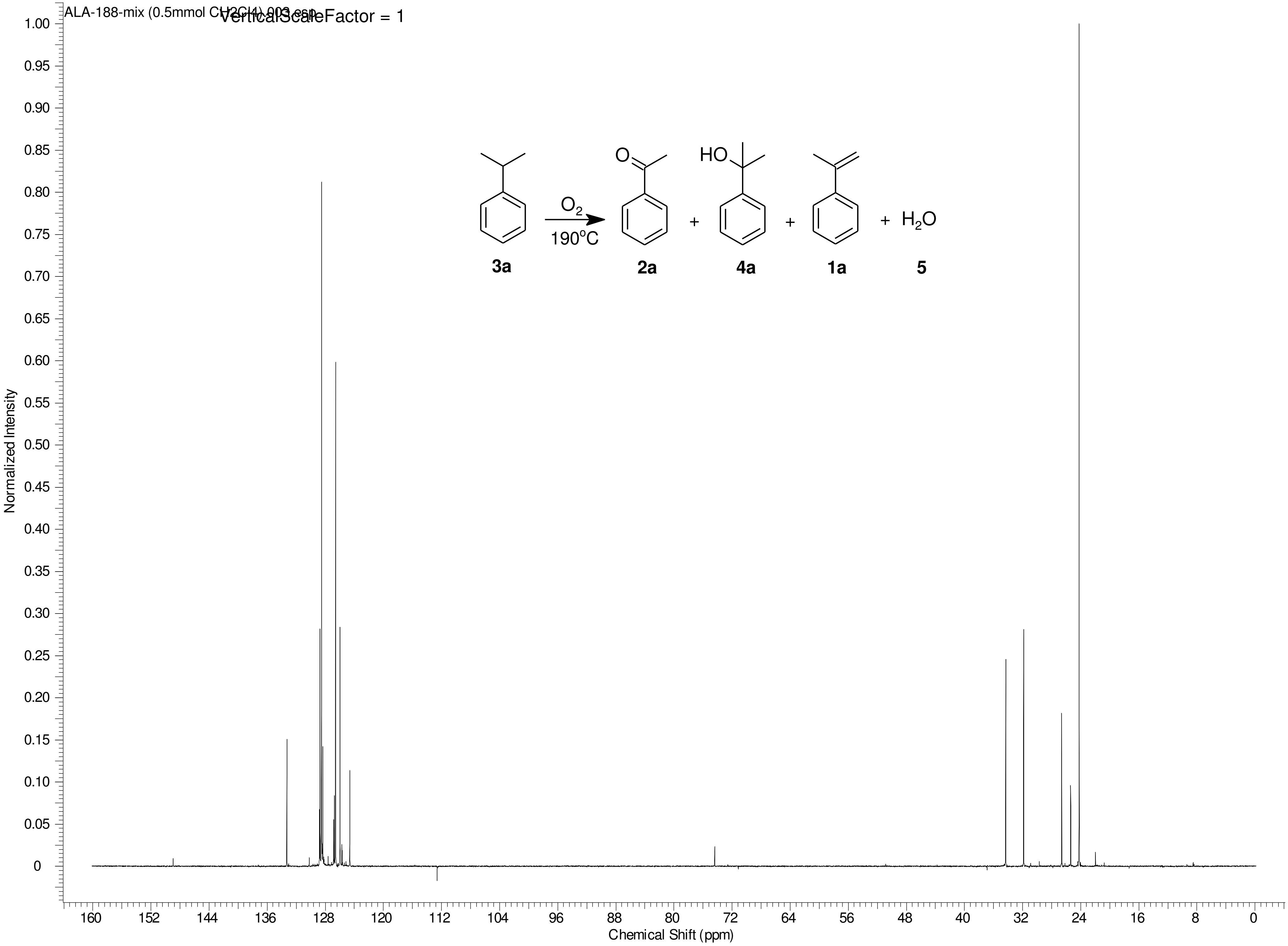
ALA-188-mix (0.5mmol CH2Cl4) 003.esp
VerticalScaleFactor = 1
Normalized Intensity
Chemical Shift (ppm)
O2
190°C
3a
2a
4a
1a
5
HO
+ H2O

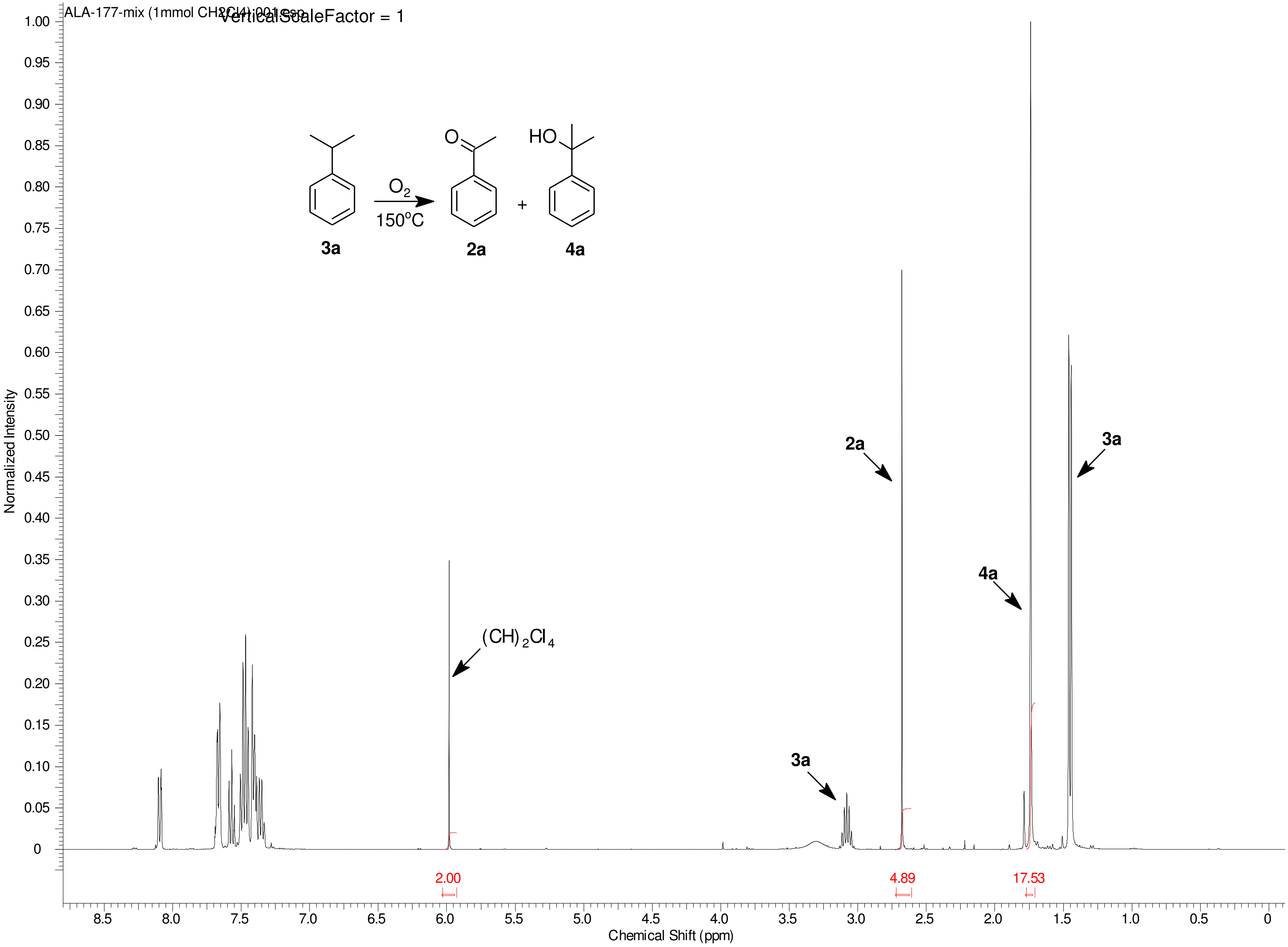
ALA-177-mix (1mmol CH2Cl4).001.esp
VerticalScaleFactor = 1
O2
150°C
3a
2a
4a
HO
O
(CH)2Cl4
2a
3a
3a
4a
2.00
4.89
17.53
Normalized Intensity
Chemical Shift (ppm)

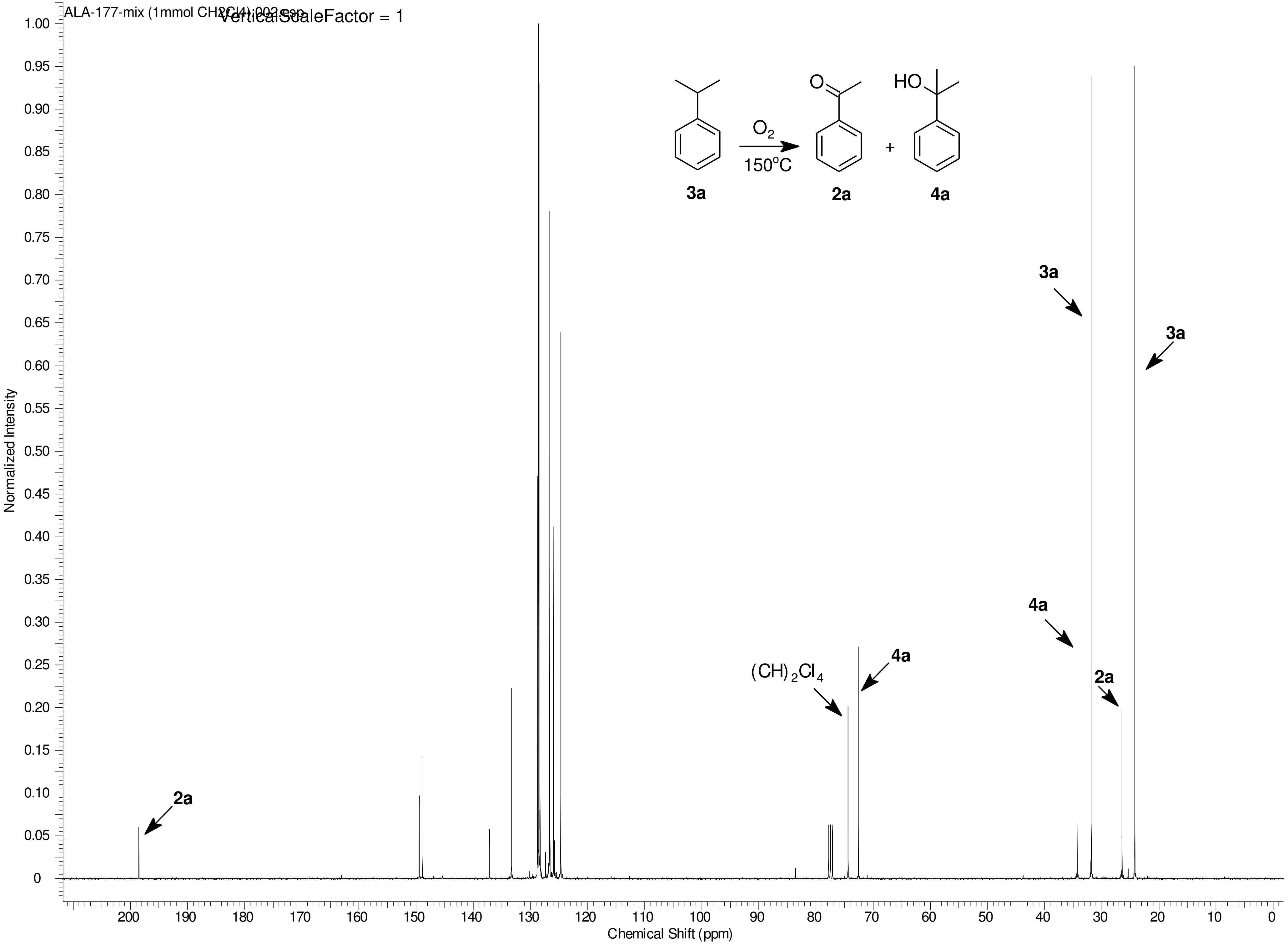

ALA-177-mix (1mmol CH2Cl4).002.esp
VerticalScaleFactor = 1
O2
150oC
3a
2a
+
4a
HO
O
3a
3a
4a
2a
(CH)2Cl4
4a
2a
Normalized Intensity
Chemical Shift (ppm)

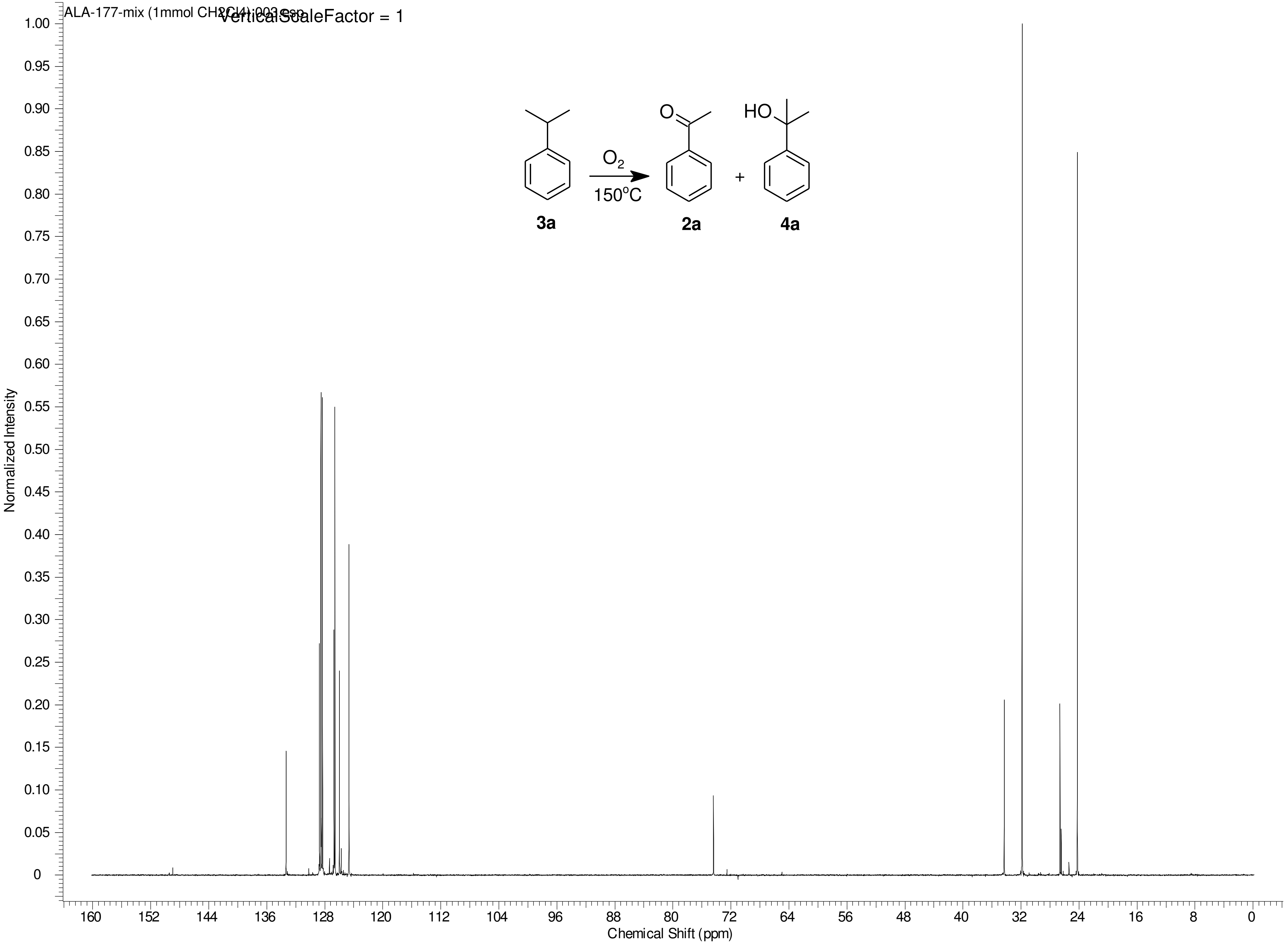

ALA-177-mix (1mmol CH2Cl4):003.esp
VerticalScaleFactor = 1
O2
150oC
3a
2a
4a
HO
O
+
Normalized Intensity
Chemical Shift (ppm)
160
152
144
136
128
120
112
104
96
88
80
72
64
56
48
40
32
24
16
8
0
1.00
0.95
0.90
0.85
0.80
0.75
0.70
0.65
0.60
0.55
0.50
0.45
0.40
0.35
0.30
0.25
0.20
0.15
0.10
0.05
0

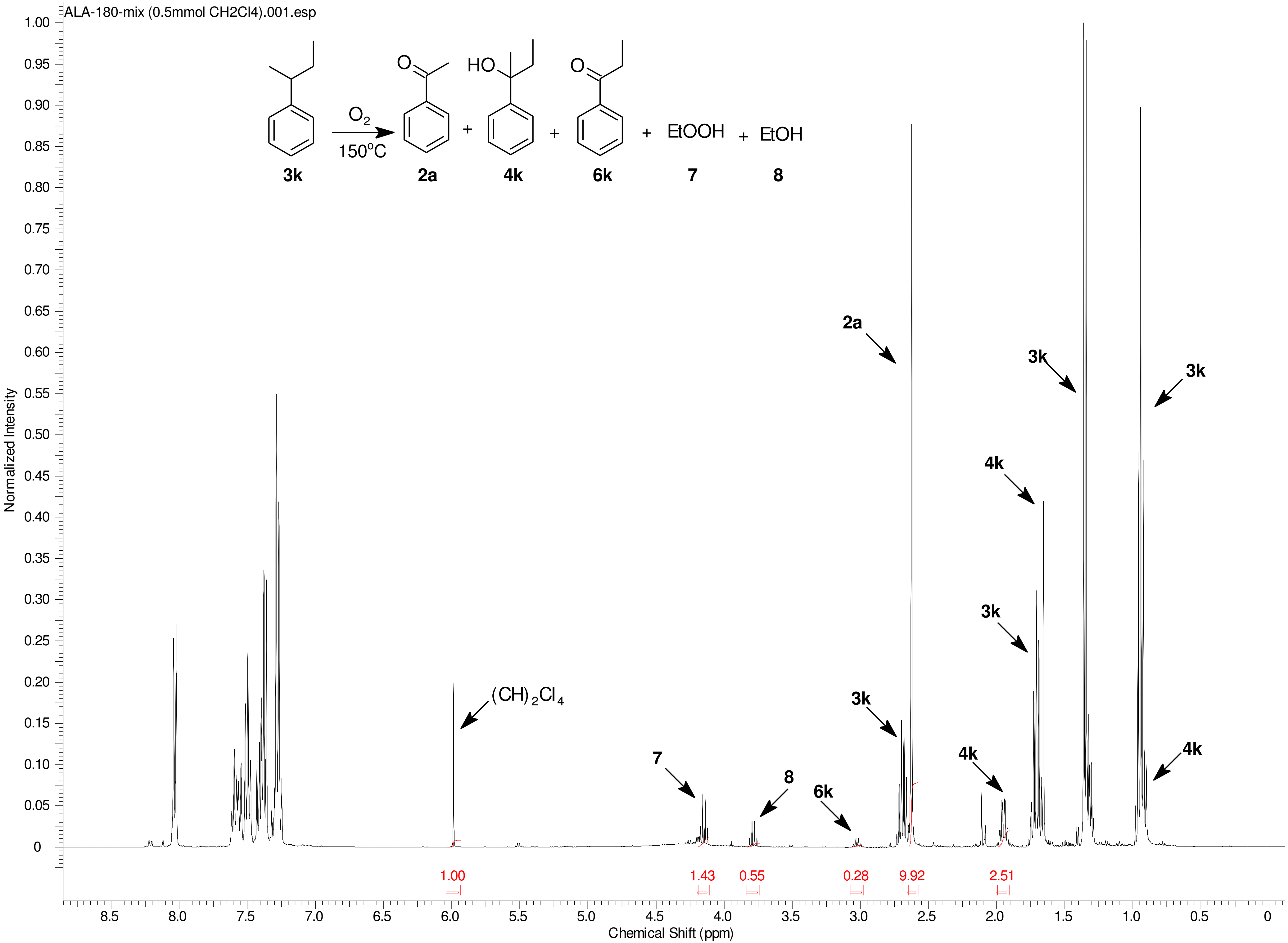

ALA-180-mix (0.5mmol CH2Cl4).001.esp
O2
150°C
3k
2a
4k
6k
EtOOH
EtOH
7
8
(CH)2Cl4
1.00
1.43
0.55
0.28
9.92
2.51
Normalized Intensity
Chemical Shift (ppm)

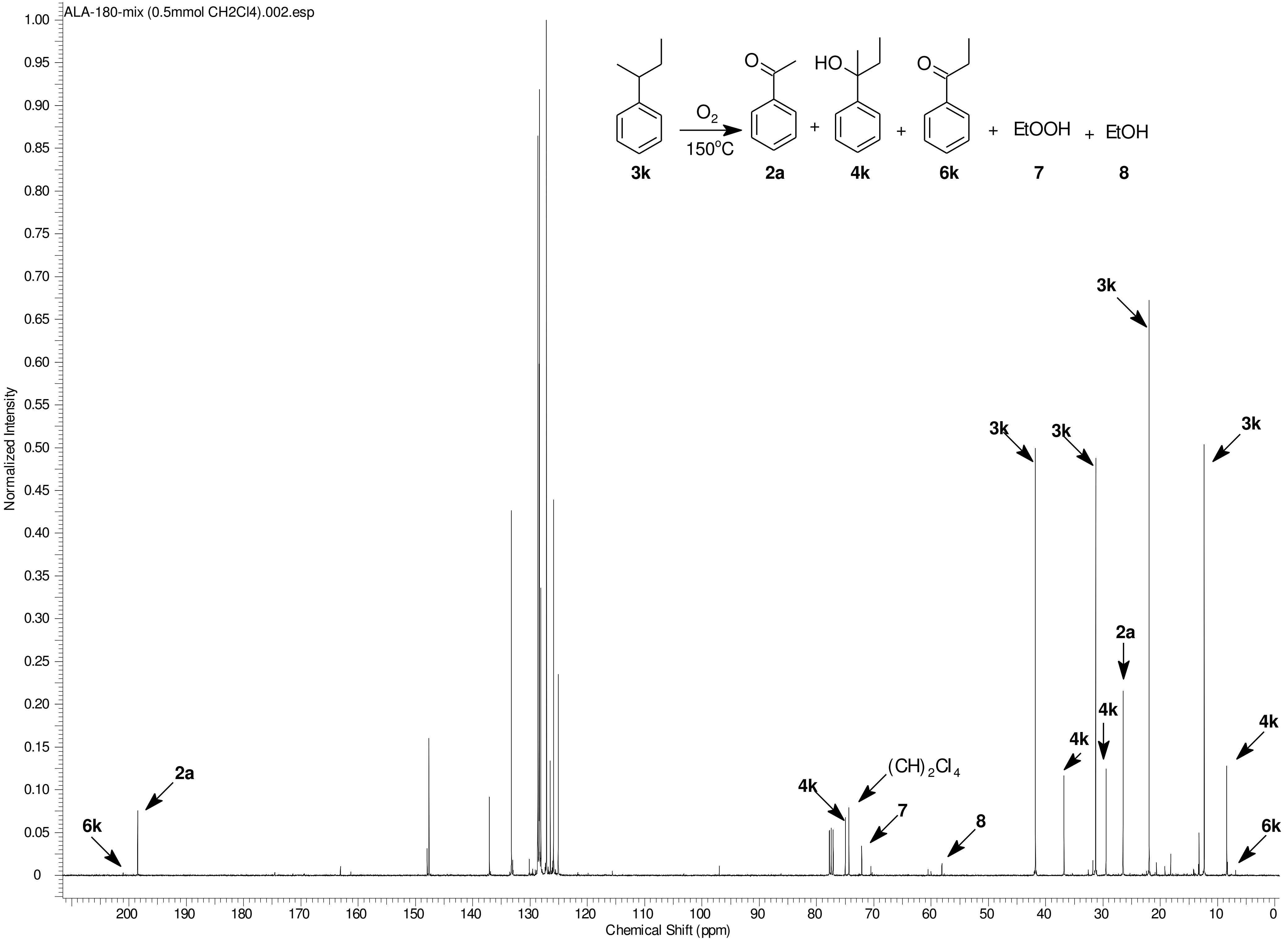

ALA-180-mix (0.5mmol CH2Cl4).002.esp
O2
150°C
3k
2a
4k
6k
7
8
EtOOH
EtOH
HO
O
(CH)2Cl4
Normalized Intensity
Chemical Shift (ppm)

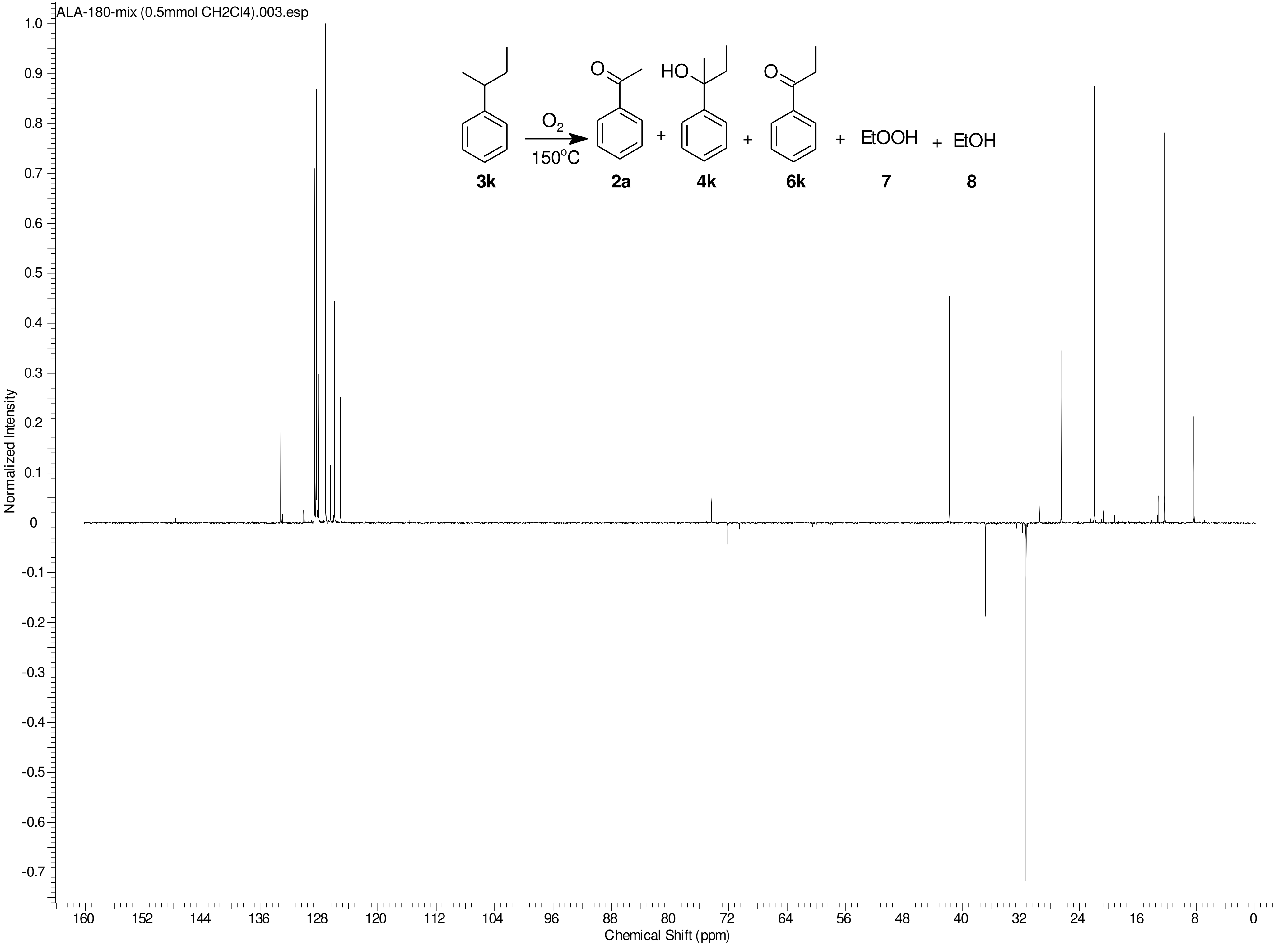

ALA-180-mix (0.5mmol CH2Cl4).003.esp
O2
150°C
3k
2a
4k
6k
EtOOH
EtOH
7
8
Normalized Intensity
Chemical Shift (ppm)

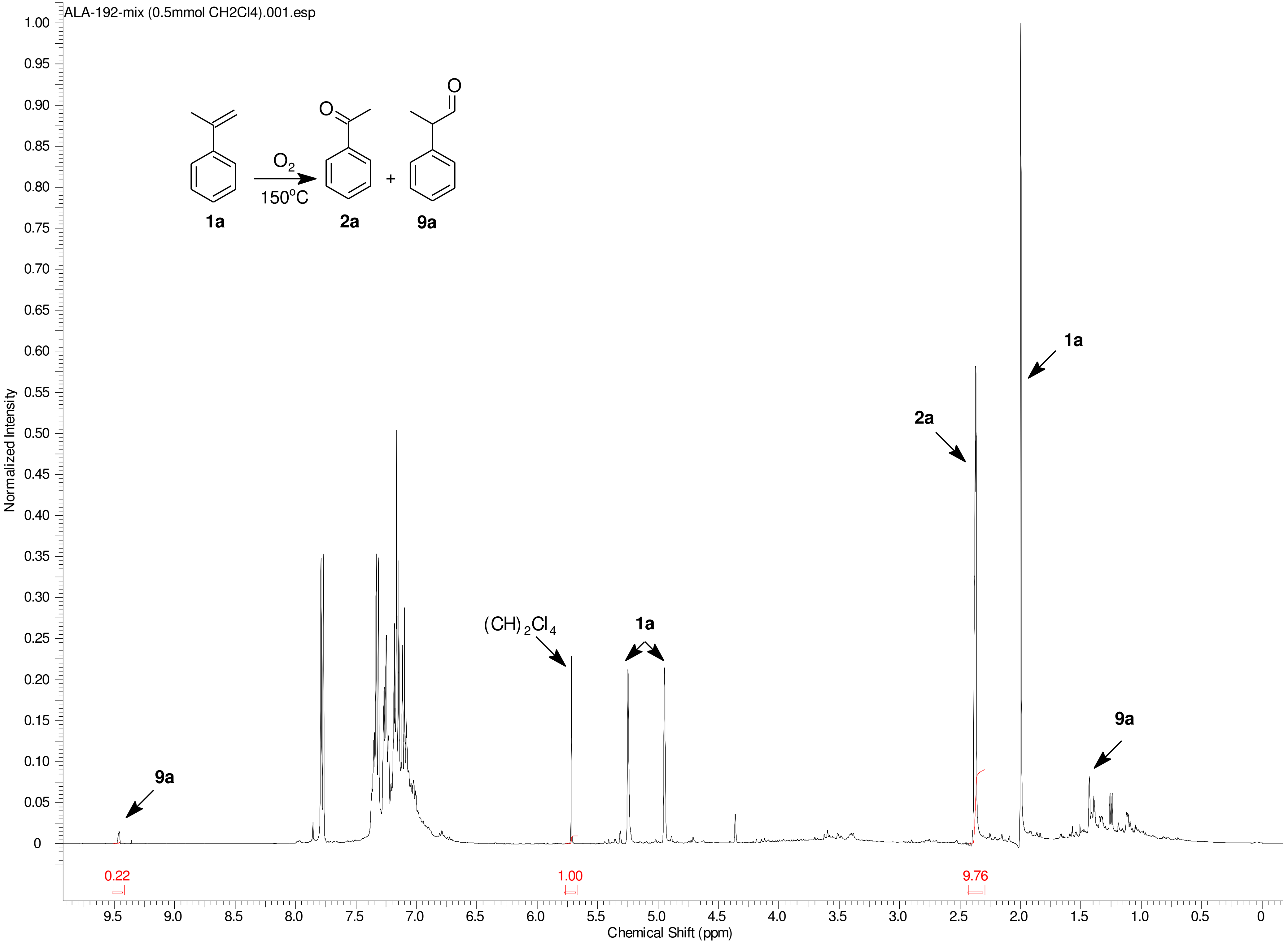

ALA-192-mix (0.5mmol CH2Cl4).001.esp
O2
150°C
1a
2a
9a
(CH)2Cl4
0.22
1.00
9.76
Normalized Intensity
Chemical Shift (ppm)

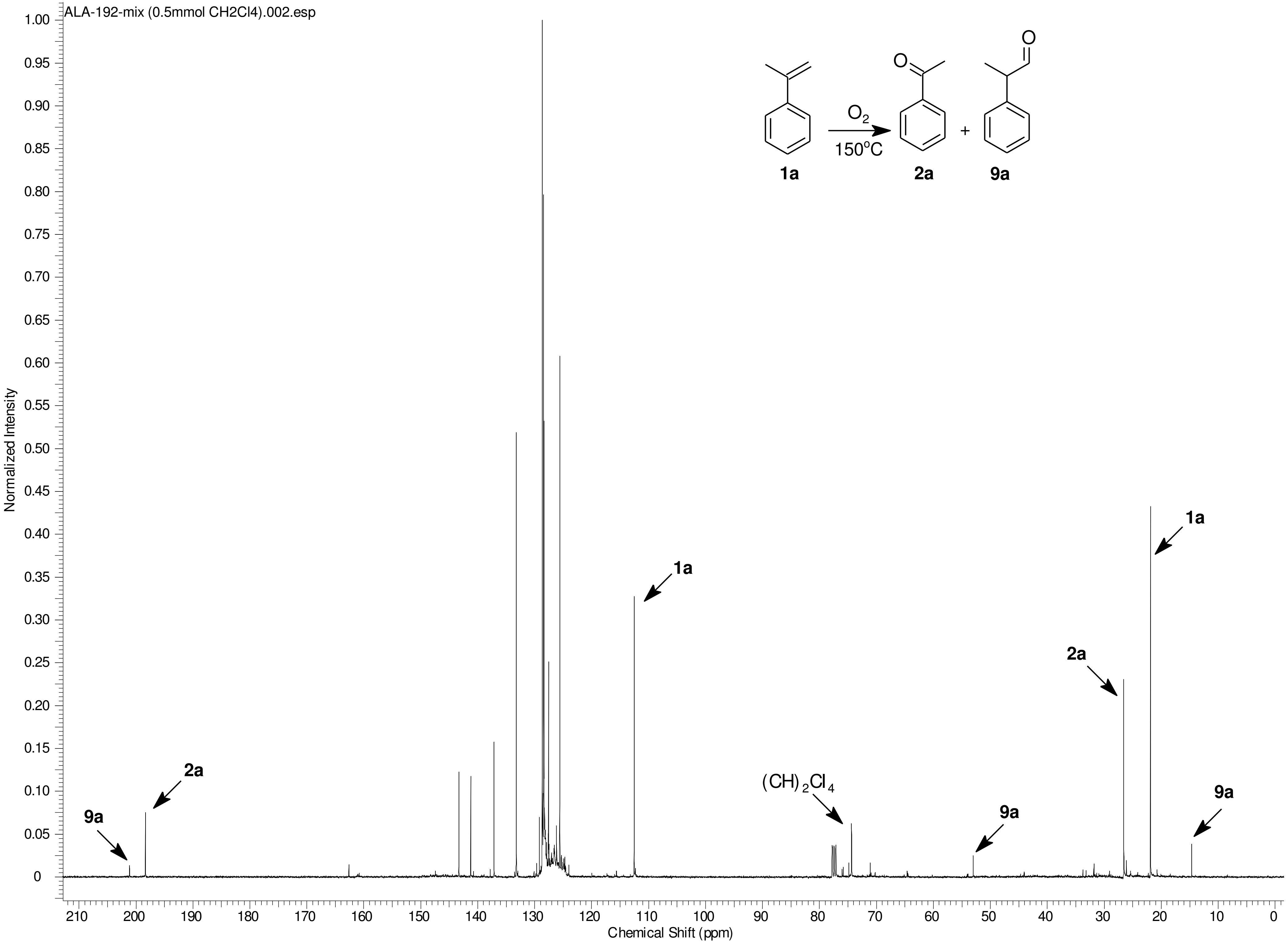

ALA-192-mix (0.5mmol CH2Cl4).002.esp
O_2
150°C
1a
2a
9a
(CH)$_2$Cl$_4$
Normalized Intensity
Chemical Shift (ppm)

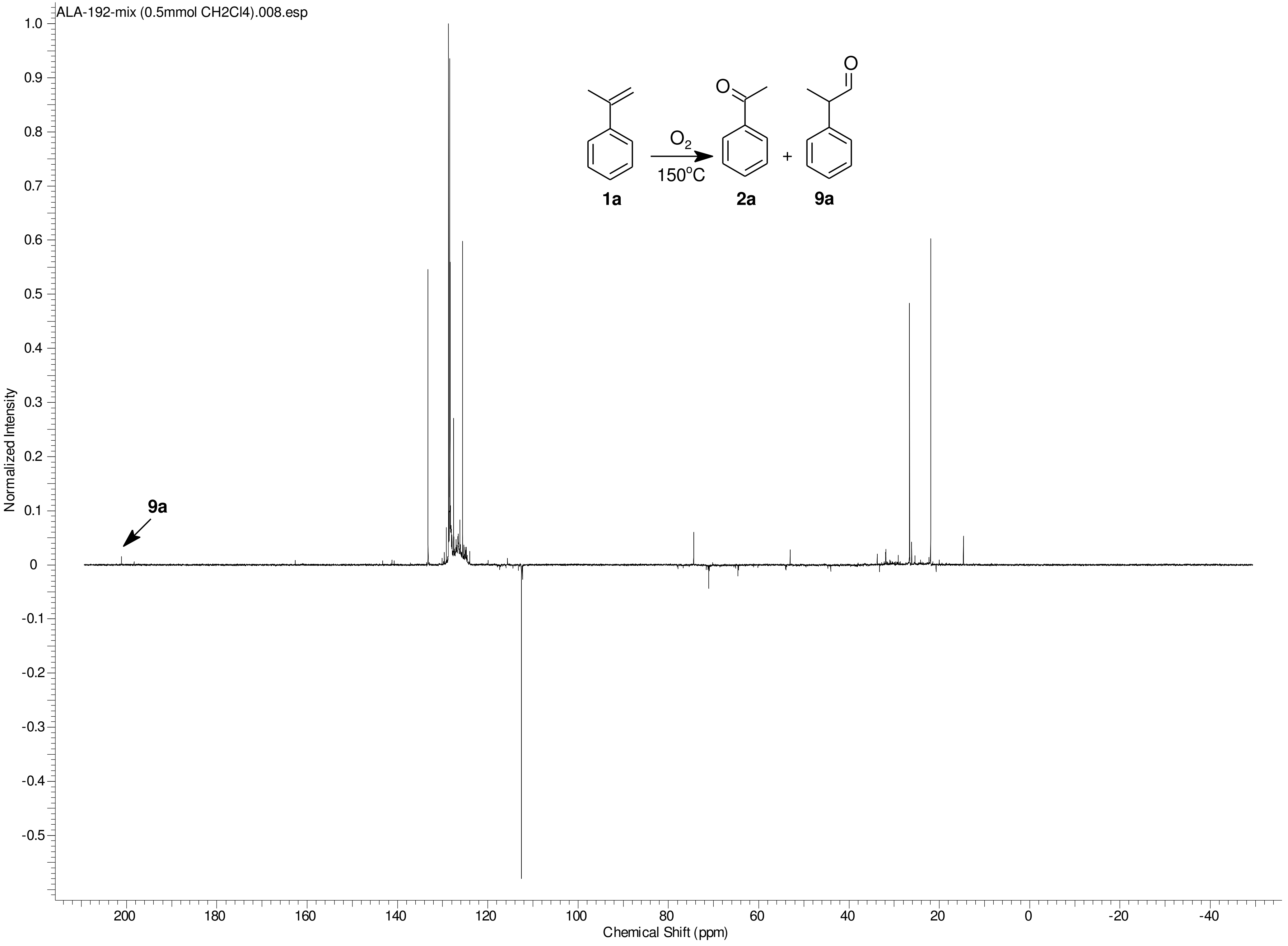

ALA-192-mix (0.5mmol CH2Cl4).008.esp
Normalized Intensity
Chemical Shift (ppm)
9a
O_2
150°C
1a
2a
9a

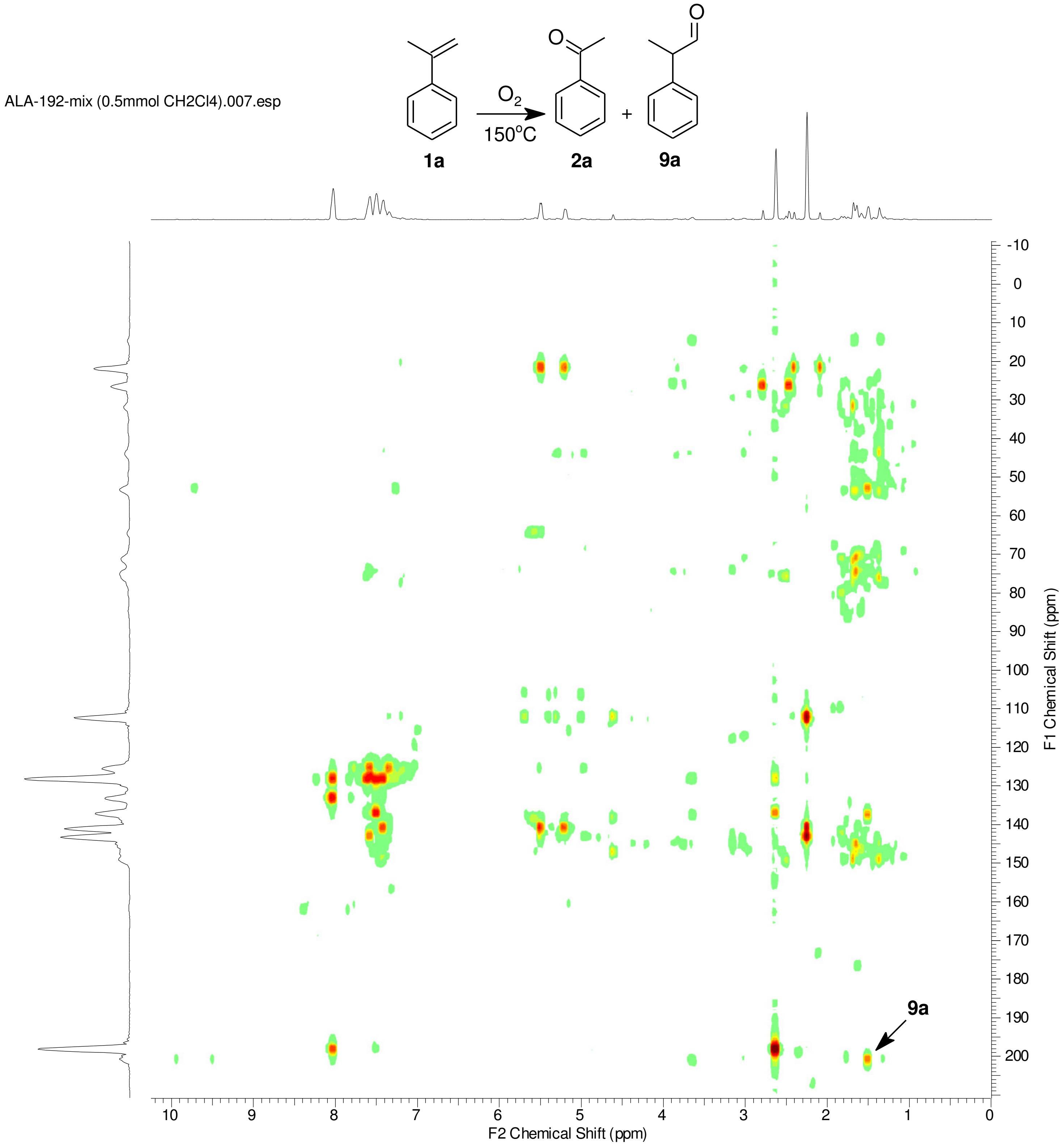
ALA-192-mix (0.5mmol CH2Cl4).007.esp
O2
150oC
1a
2a
+
9a
9a
F2 Chemical Shift (ppm)
F1 Chemical Shift (ppm)

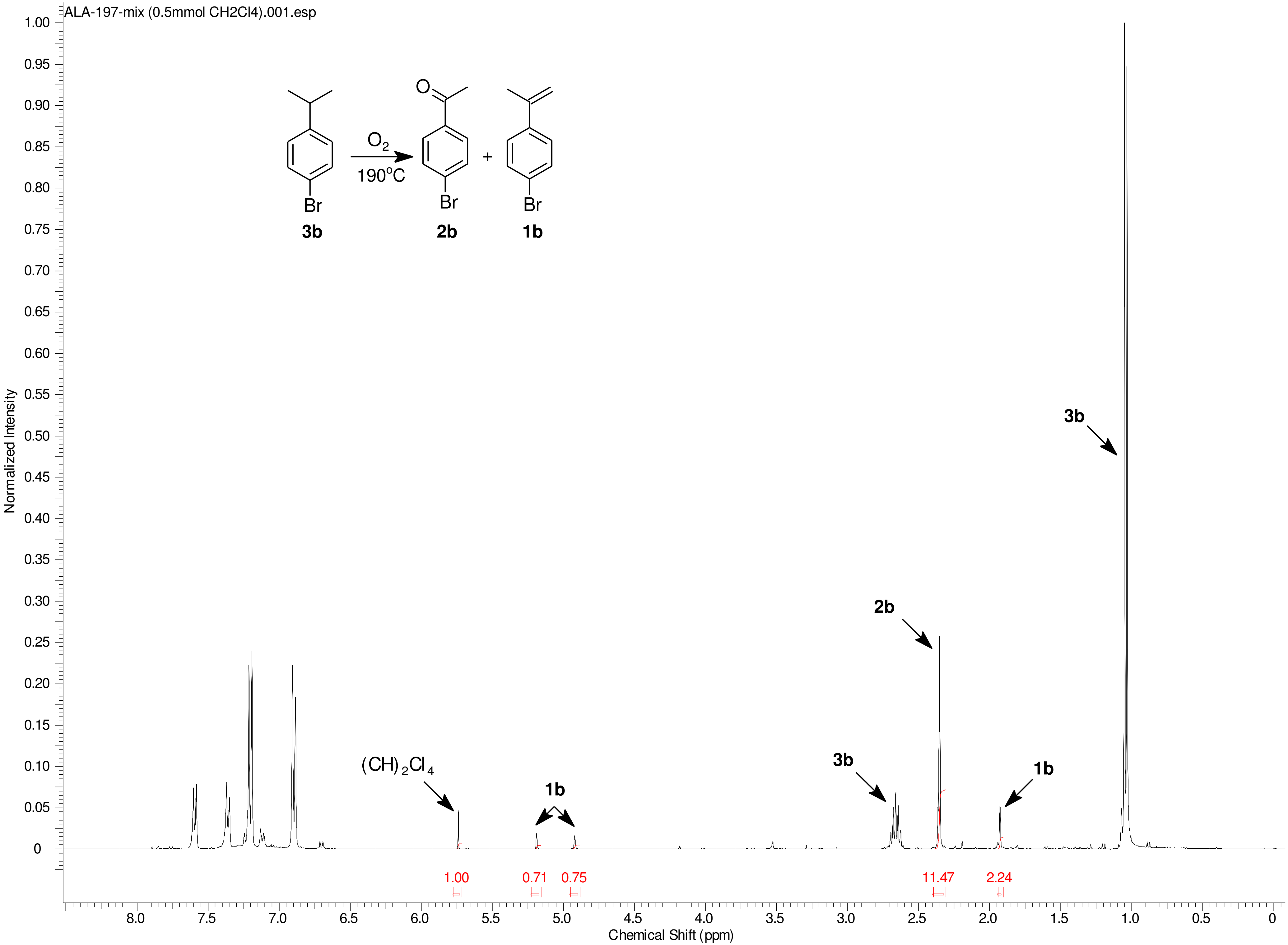

ALA-197-mix (0.5mmol CH2Cl4).001.esp
O2
190°C
+
Br
3b
2b
1b
(CH)2Cl4
1.00
0.71
0.75
11.47
2.24
Normalized Intensity
Chemical Shift (ppm)

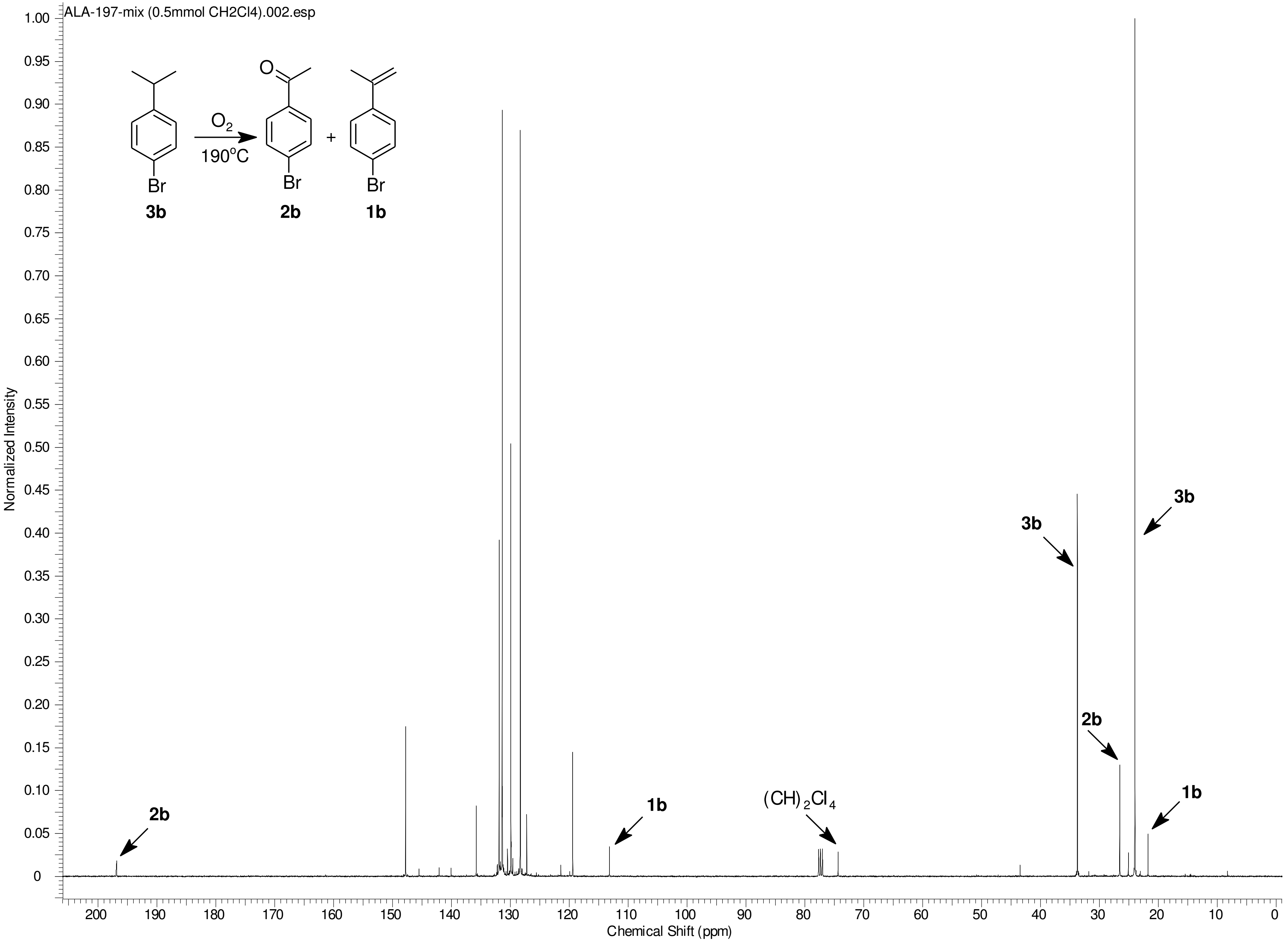

ALA-197-mix (0.5mmol CH2Cl4).002.esp
O_2
190°C
Br
3b
2b
1b
(CH)$_2$Cl$_4$
Normalized Intensity
Chemical Shift (ppm)

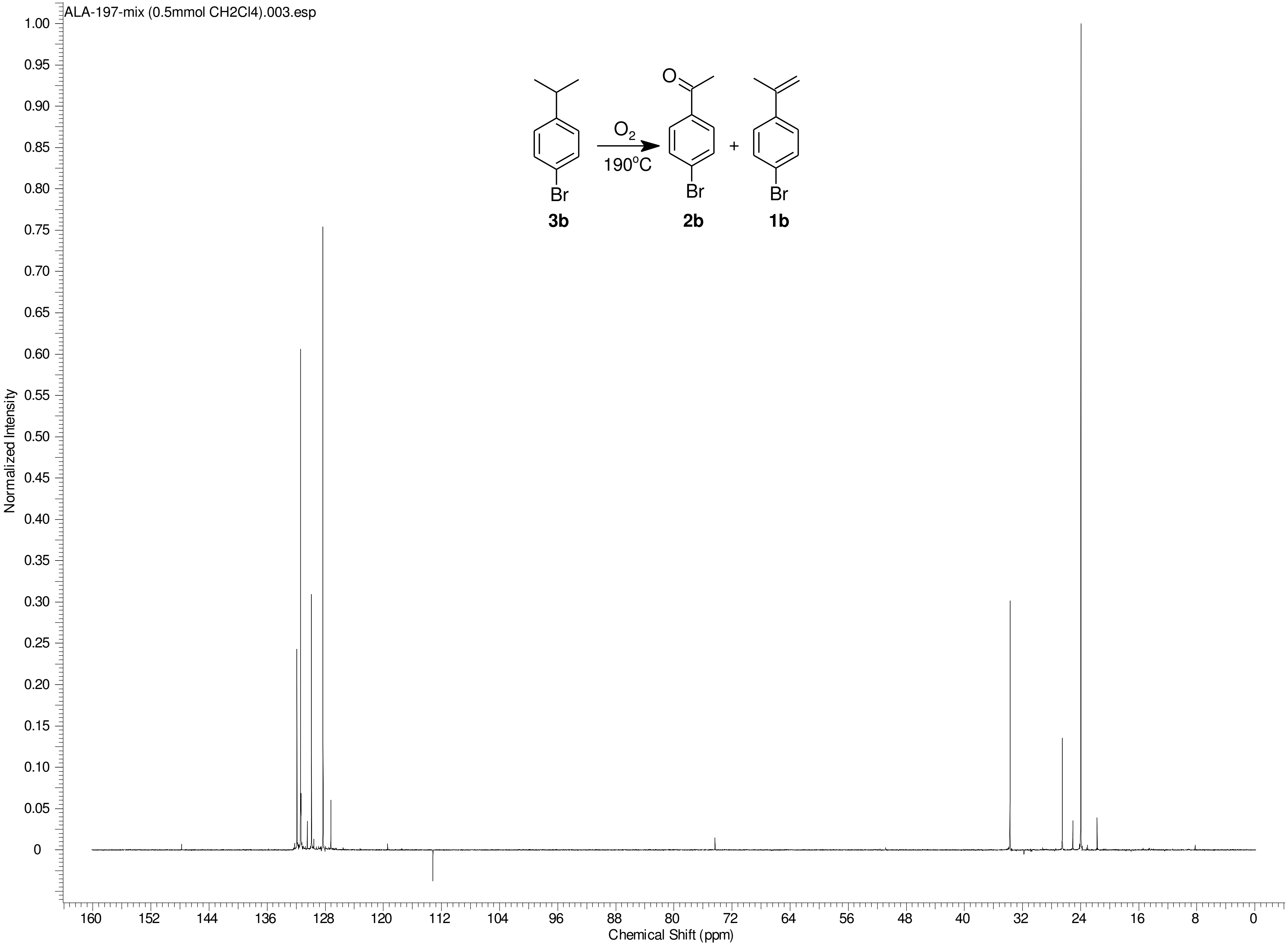
ALA-197-mix (0.5mmol CH2Cl4).003.esp
O
O_2
190°C
+
Br
Br
Br
3b
2b
1b
Normalized Intensity
Chemical Shift (ppm)
1.00
0.95
0.90
0.85
0.80
0.75
0.70
0.65
0.60
0.55
0.50
0.45
0.40
0.35
0.30
0.25
0.20
0.15
0.10
0.05
0
160
152
144
136
128
120
112
104
96
88
80
72
64
56
48
40
32
24
16
8
0

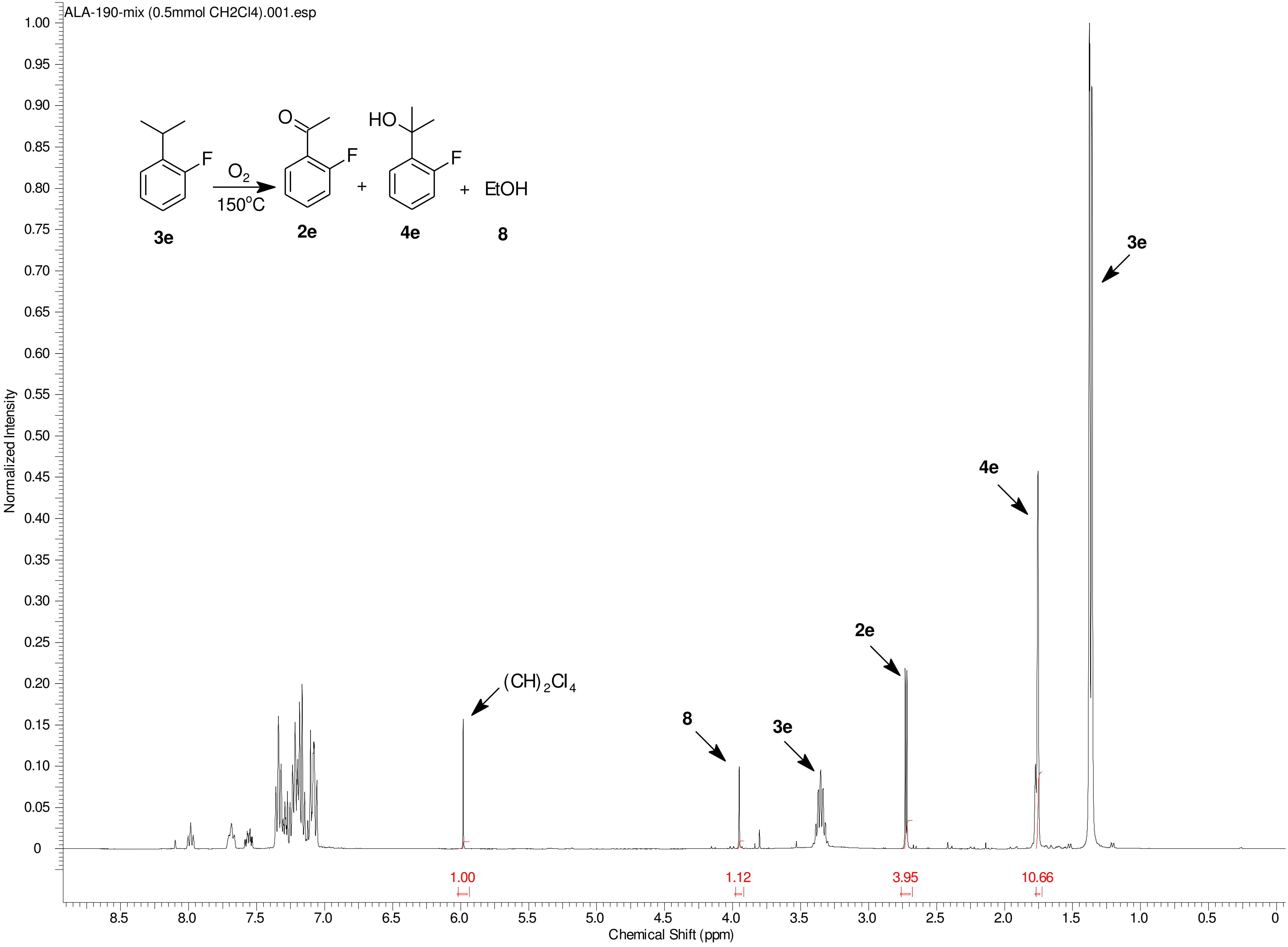

ALA-190-mix (0.5mmol CH2Cl4).001.esp
O$_2$
150°C
+
+ EtOH
3e
2e
4e
8
(CH)$_2$Cl$_4$
8
3e
2e
4e
3e
1.00
1.12
3.95
10.66
Normalized Intensity
Chemical Shift (ppm)

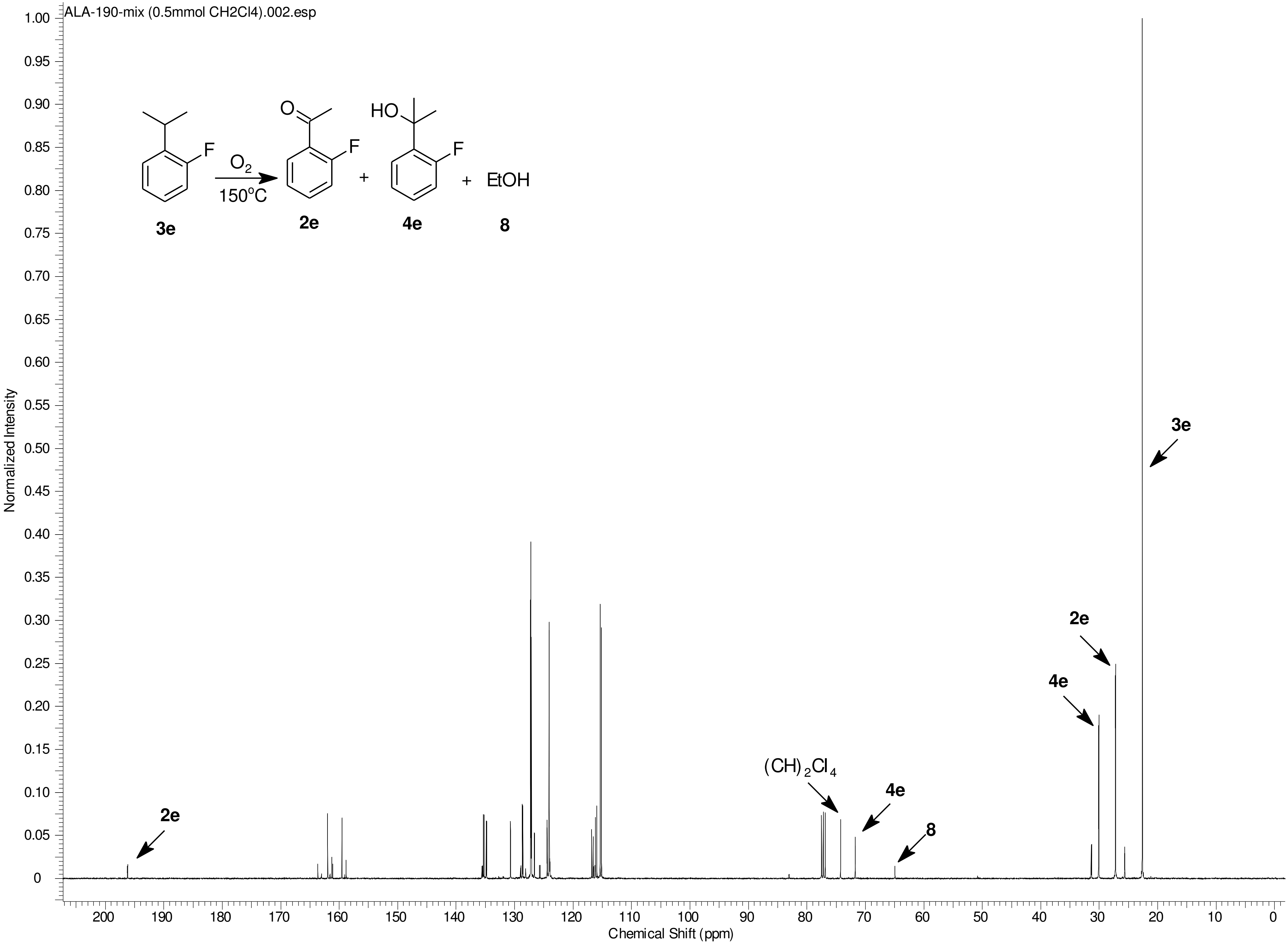

ALA-190-mix (0.5mmol CH2Cl4).002.esp
O2
150°C
+
+ EtOH
3e
2e
4e
8
(CH)2Cl4
Normalized Intensity
Chemical Shift (ppm)

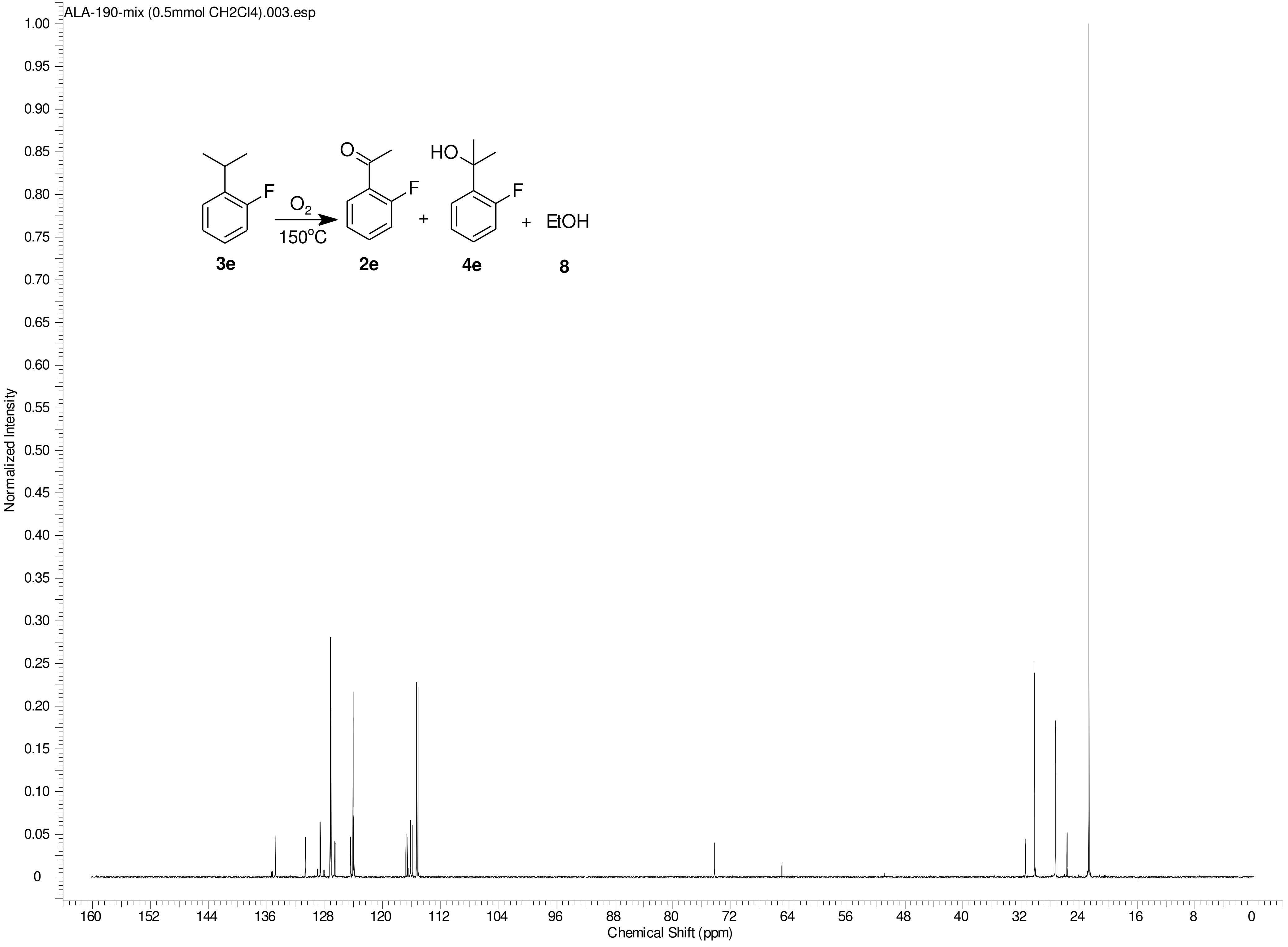
ALA-190-mix (0.5mmol CH2Cl4).003.esp
O2
150°C
3e
2e
4e
8
EtOH
Normalized Intensity
Chemical Shift (ppm)

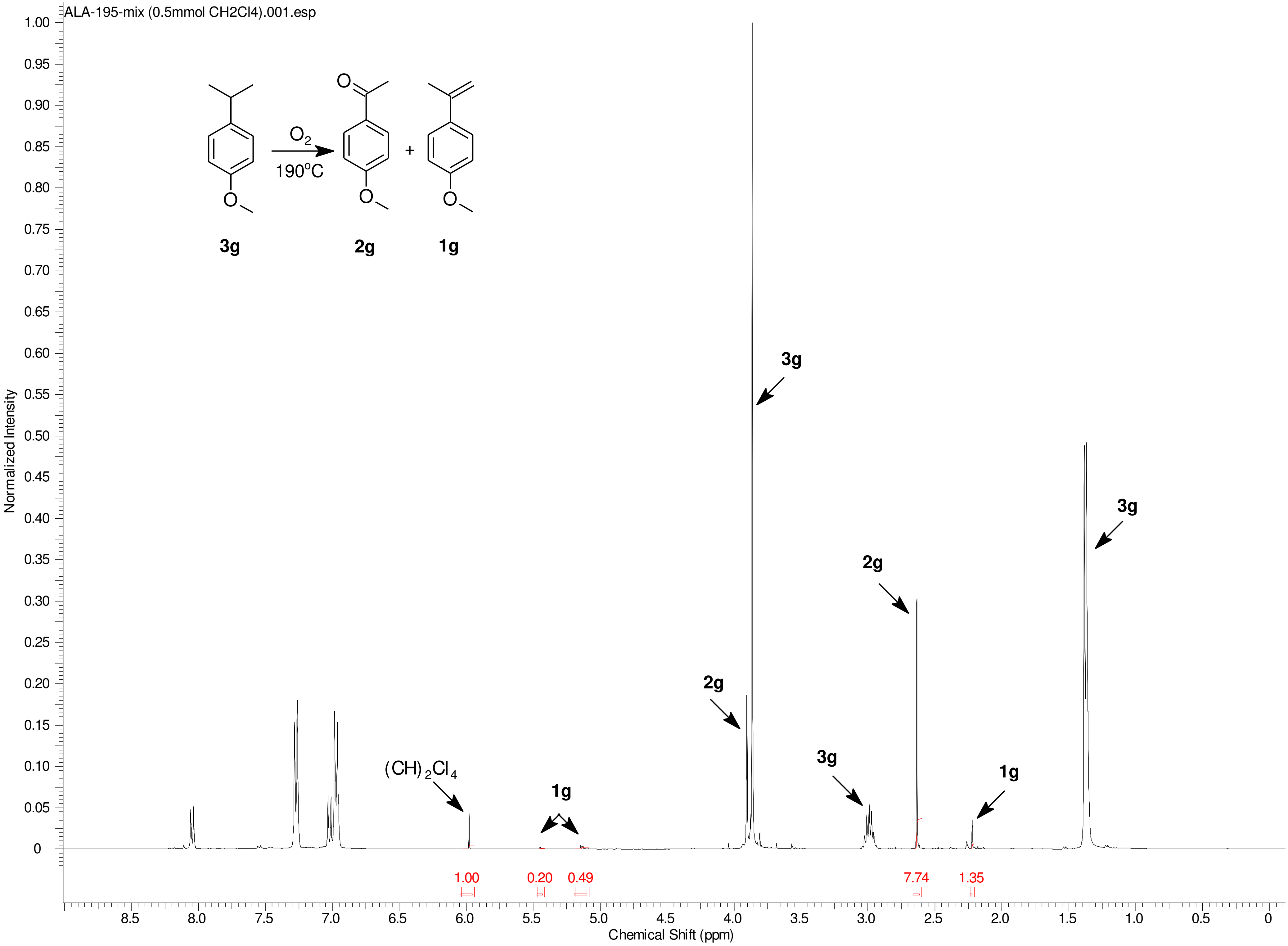

ALA-195-mix (0.5mmol CH2Cl4).001.esp
O2
190°C
3g
2g
1g
(CH)2Cl4
1.00
0.20
0.49
7.74
1.35
Normalized Intensity
Chemical Shift (ppm)

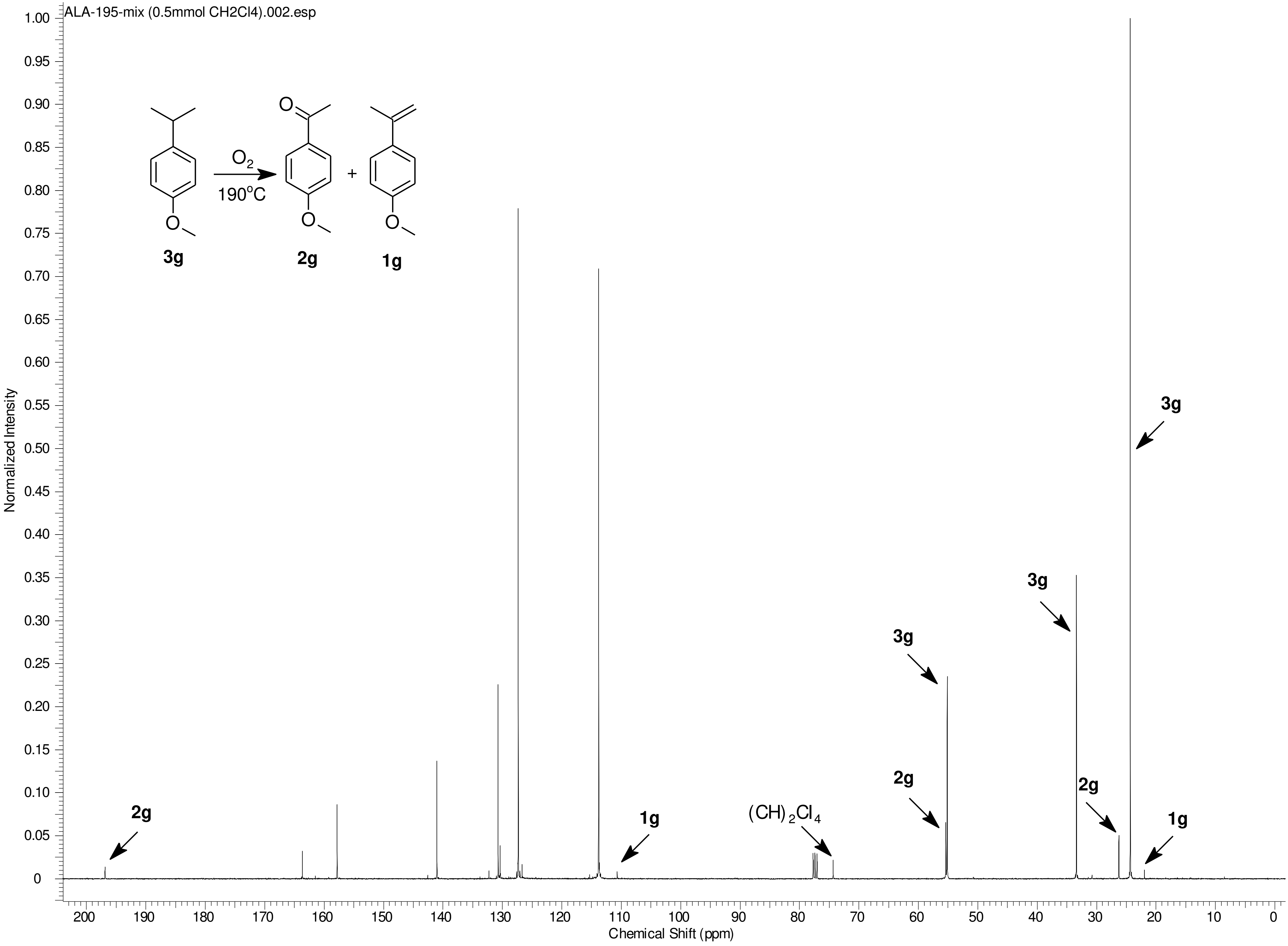
ALA-195-mix (0.5mmol CH2Cl4).002.esp
O_2
190°C
3g
2g
1g
2g
1g
(CH)$_2$Cl$_4$
3g
2g
3g
2g
3g
1g
Normalized Intensity
Chemical Shift (ppm)

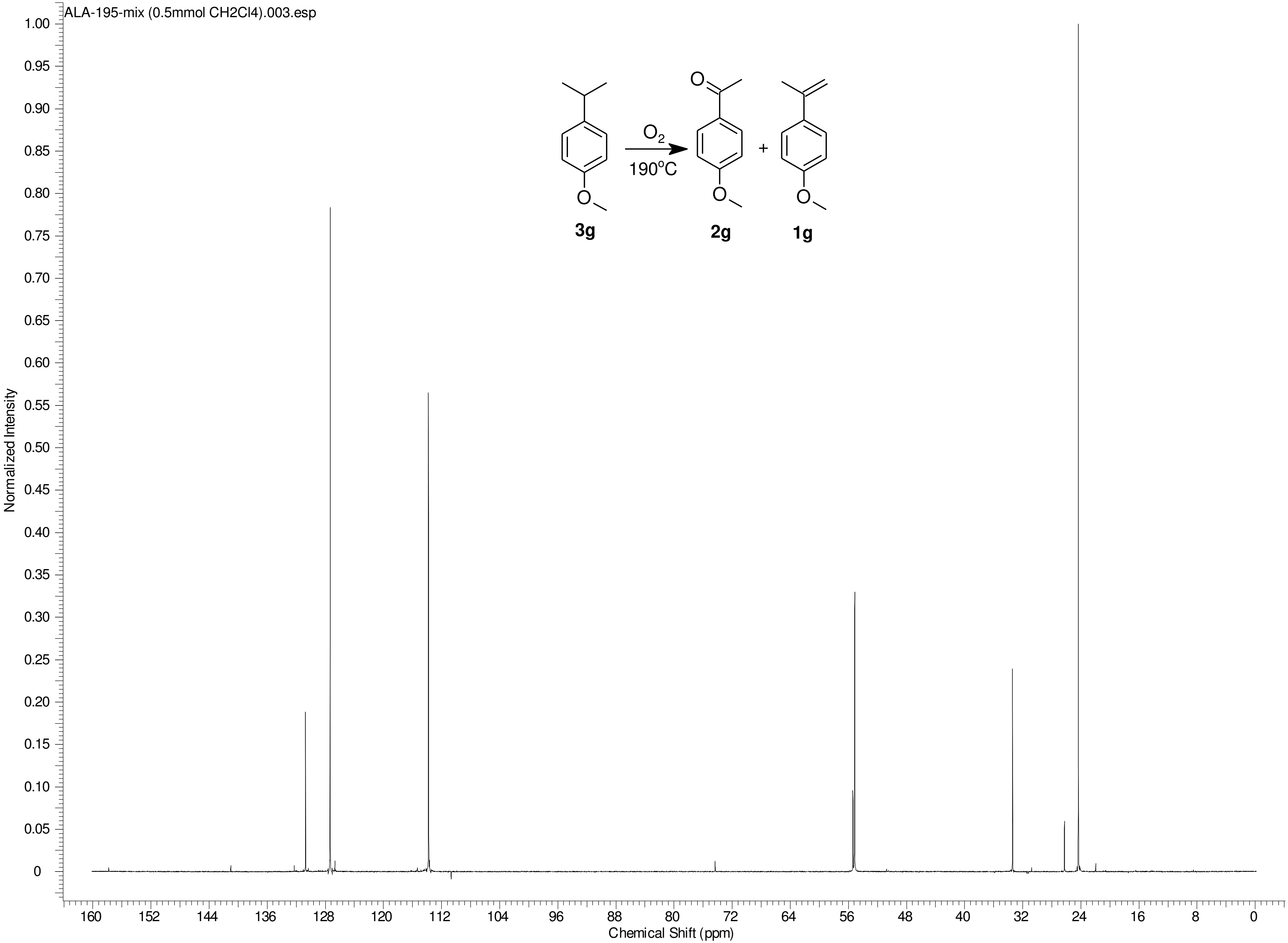
ALA-195-mix (0.5mmol CH2Cl4).003.esp
O_2
190°C
3g
2g
1g
Normalized Intensity
Chemical Shift (ppm)

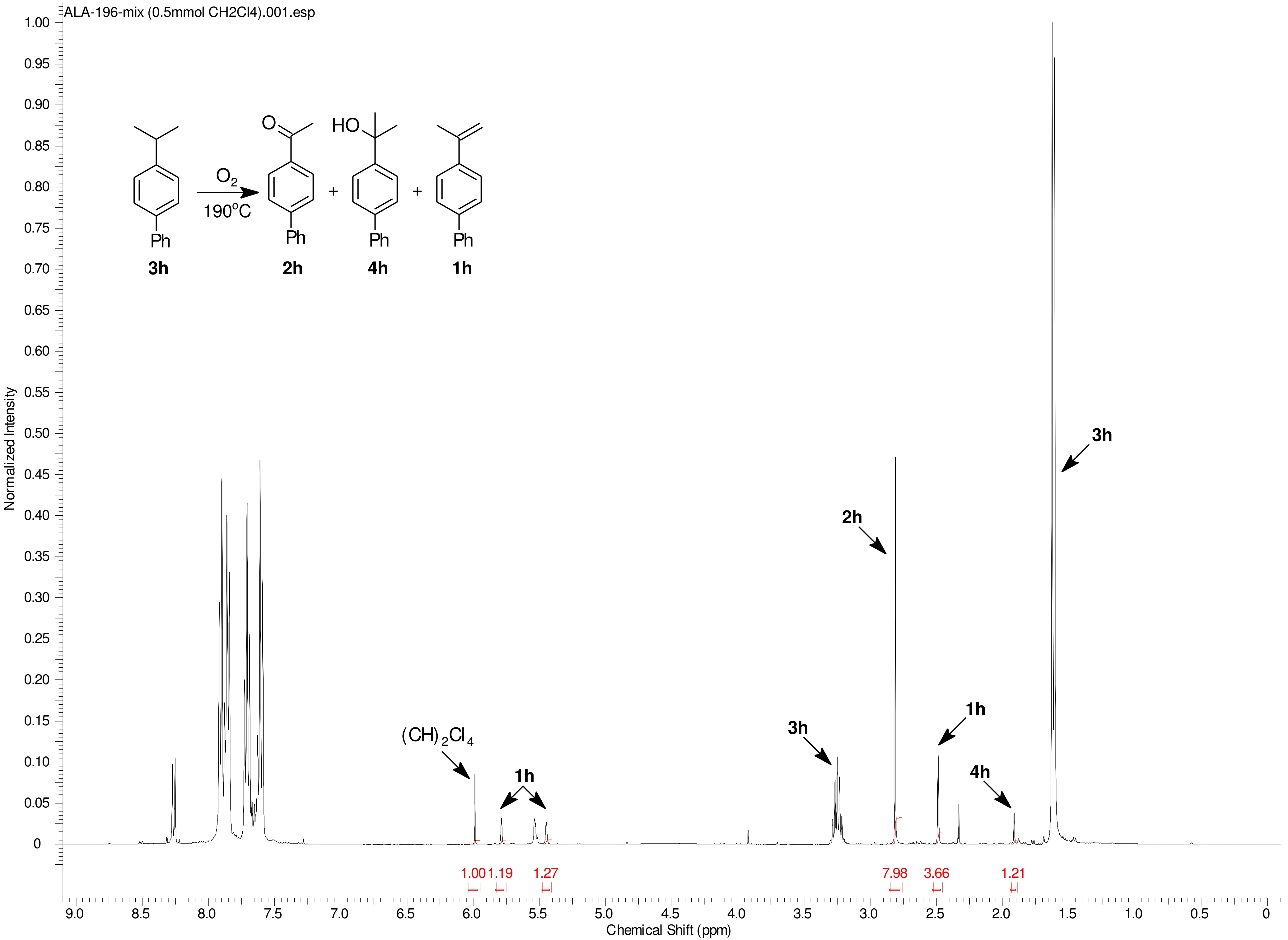
ALA-196-mix (0.5mmol CH2Cl4).001.esp
O2
190oC
Ph
3h
2h
4h
1h
HO
(CH)2Cl4
1.00
1.19
1.27
7.98
3.66
1.21
Normalized Intensity
Chemical Shift (ppm)

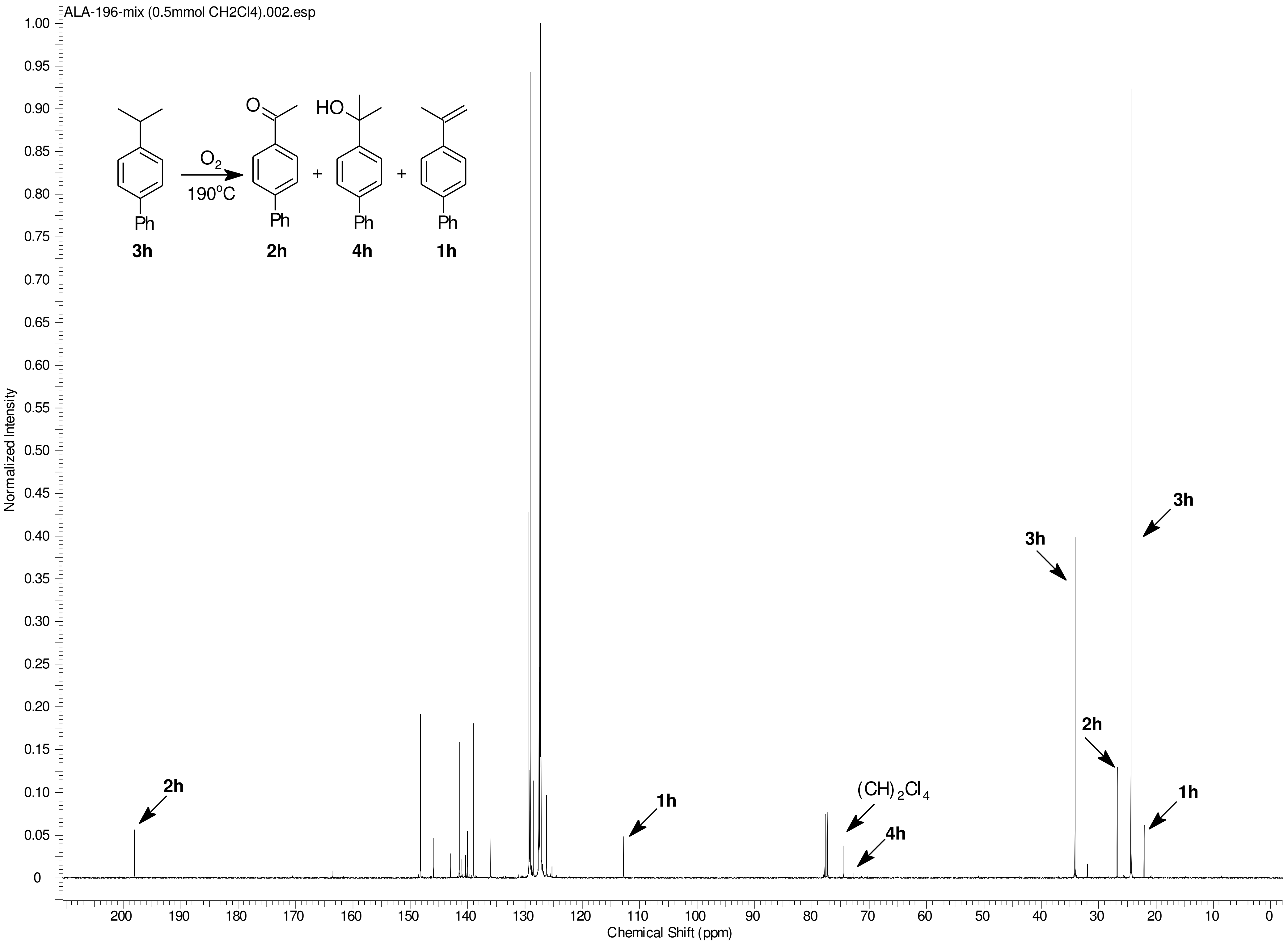

ALA-196-mix (0.5mmol CH2Cl4).002.esp
O2
190°C
Ph
3h
2h
4h
1h
HO
O
+
Normalized Intensity
Chemical Shift (ppm)
2h
1h
(CH)2Cl4
4h
3h
3h
2h
1h

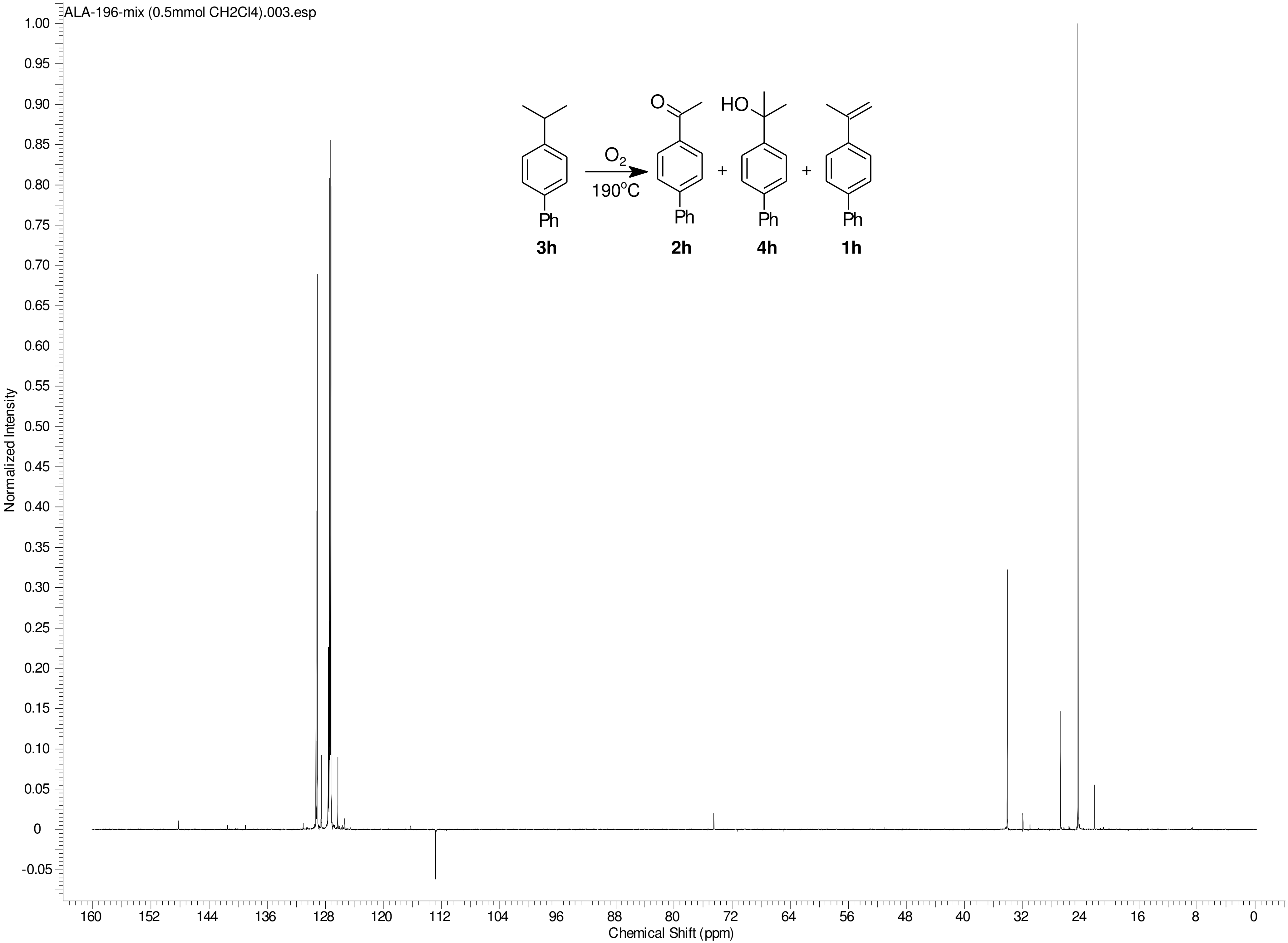
ALA-196-mix (0.5mmol CH2Cl4).003.esp
O2
190°C
Ph
3h
2h
4h
1h
HO
Normalized Intensity
Chemical Shift (ppm)

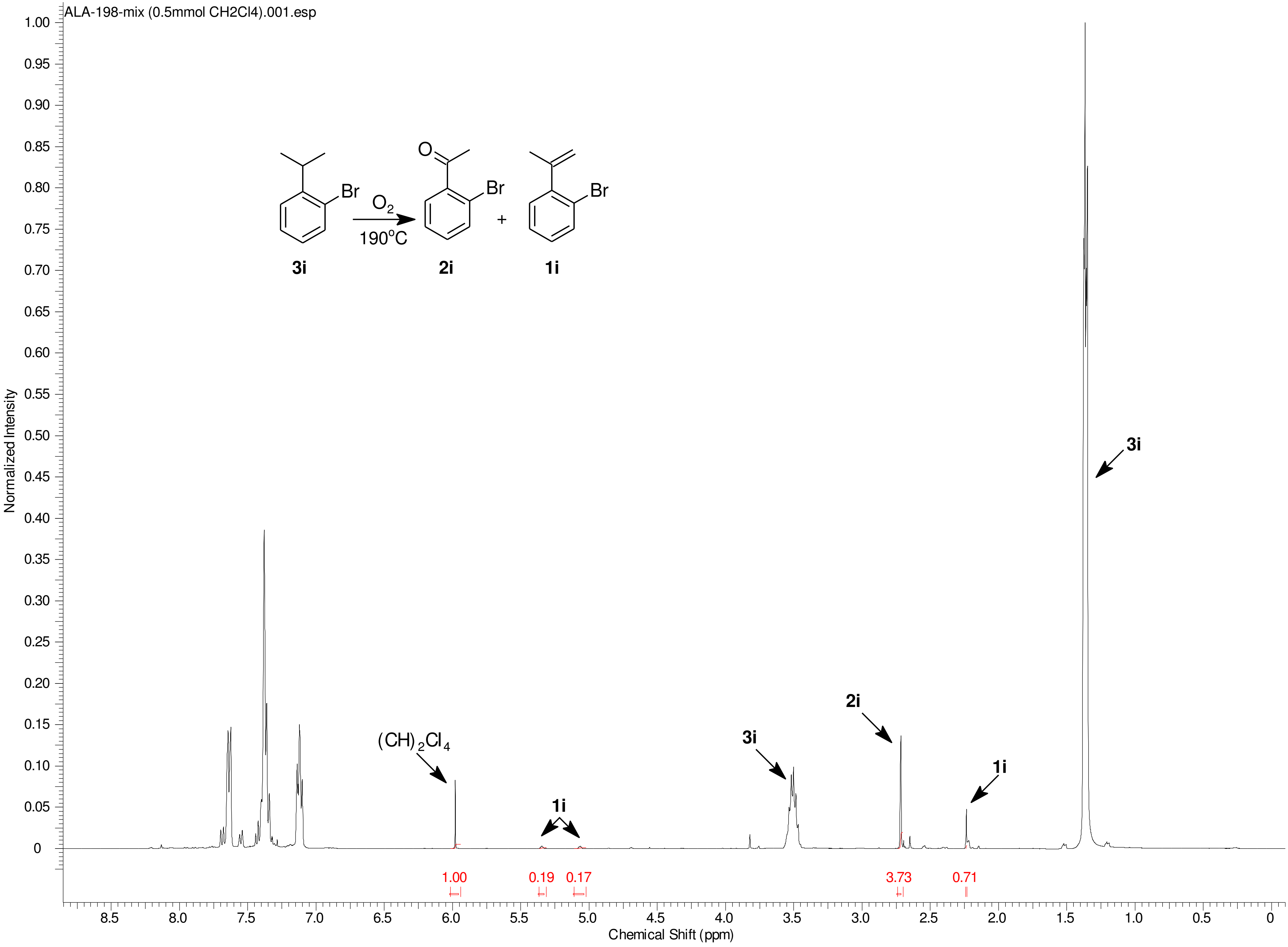

ALA-198-mix (0.5mmol CH2Cl4).001.esp
Br
O2
190°C
Br
+
Br
3i
2i
1i
(CH)2Cl4
1i
3i
2i
1i
3i
1.00
0.19
0.17
3.73
0.71
Normalized Intensity
Chemical Shift (ppm)

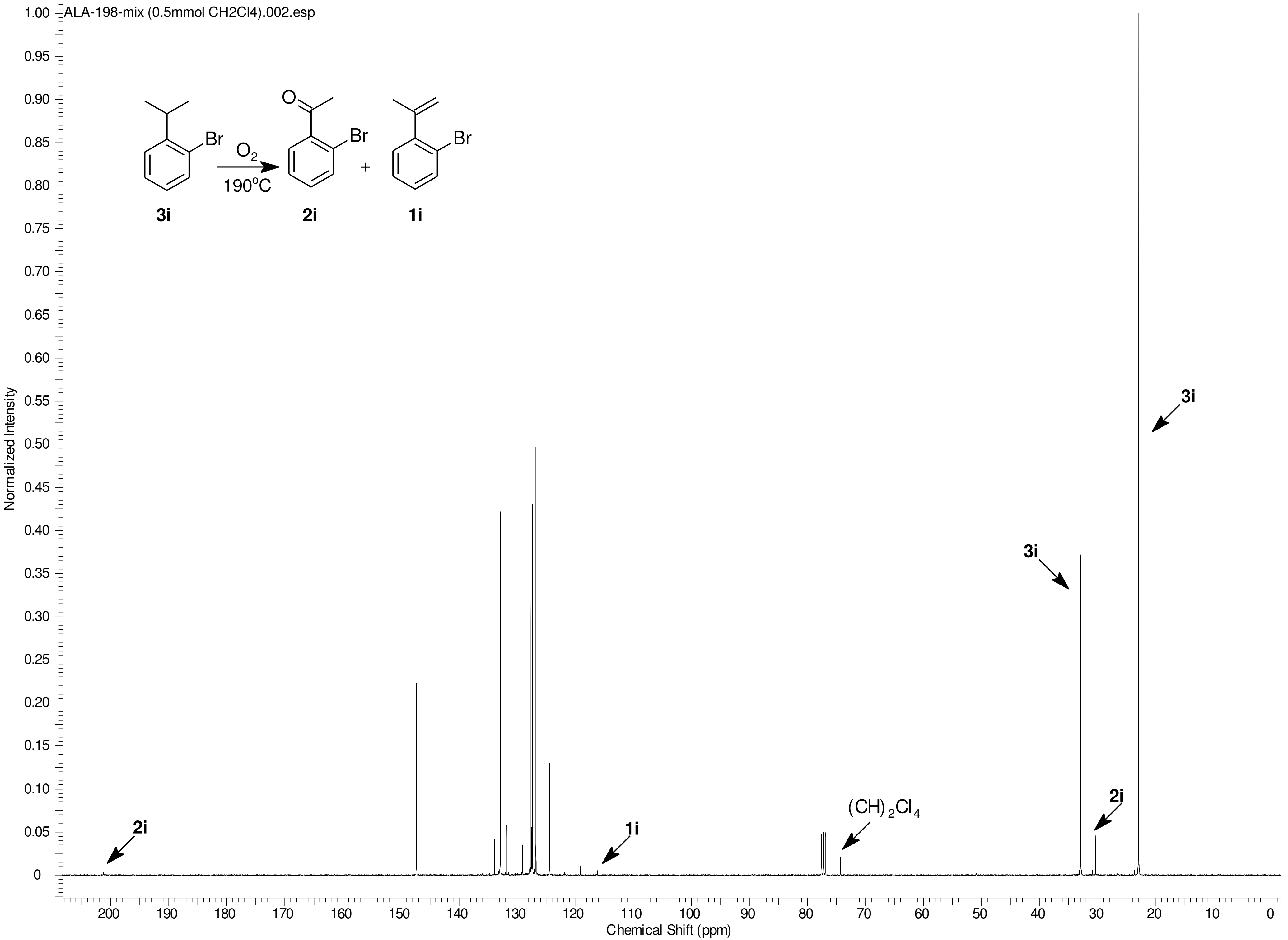
ALA-198-mix (0.5mmol CH2Cl4).002.esp
Br
O2
190°C
Br
+
Br
3i
2i
1i
3i
3i
2i
2i
1i
(CH)2Cl4
Normalized Intensity
Chemical Shift (ppm)

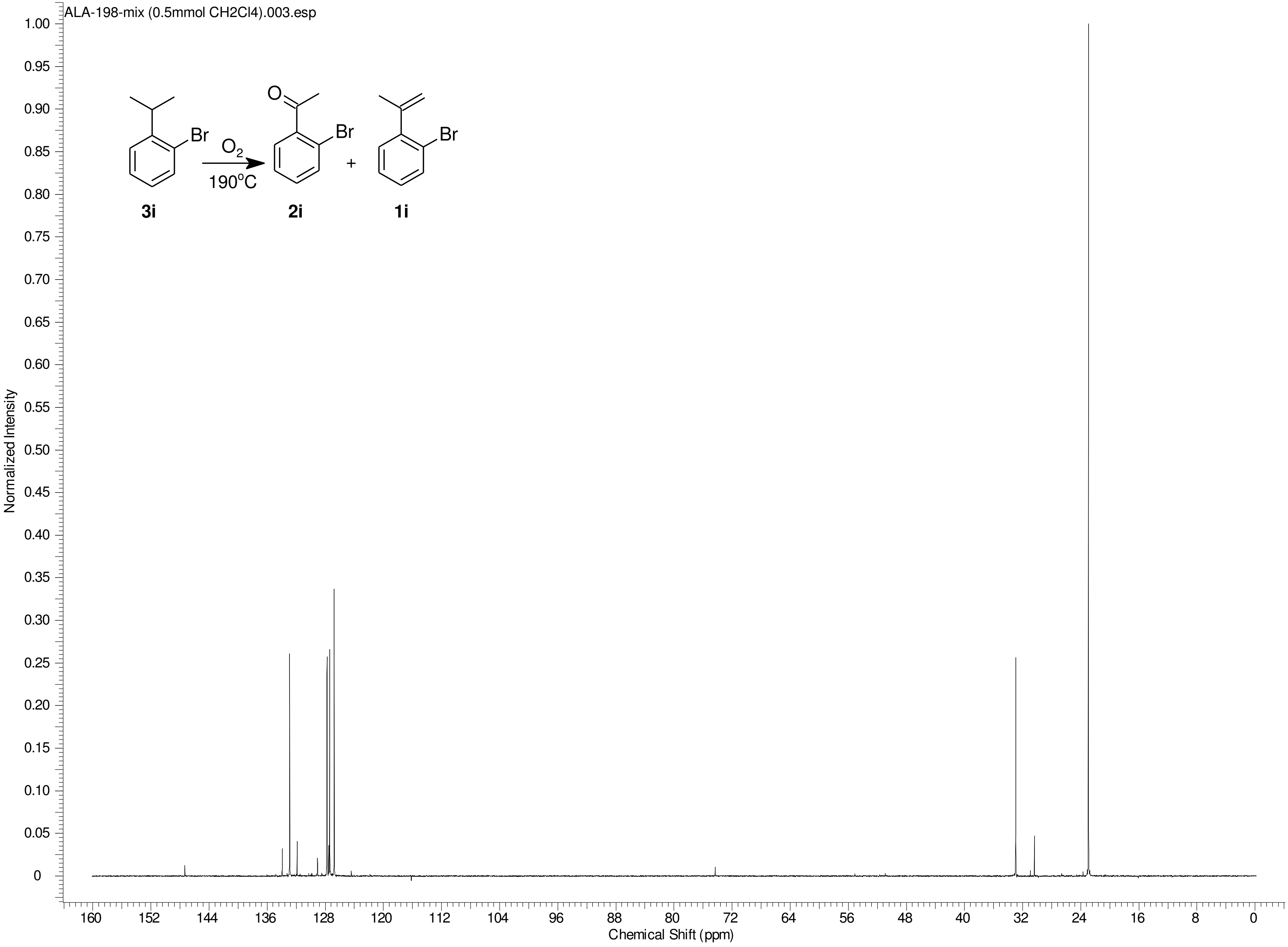
ALA-198-mix (0.5mmol CH2Cl4).003.esp
O2
190°C
Br
O
+
3i
2i
1i
Normalized Intensity
Chemical Shift (ppm)
1.00
0.95
0.90
0.85
0.80
0.75
0.70
0.65
0.60
0.55
0.50
0.45
0.40
0.35
0.30
0.25
0.20
0.15
0.10
0.05
0
160
152
144
136
128
120
112
104
96
88
80
72
64
56
48
40
32
24
16
8
0

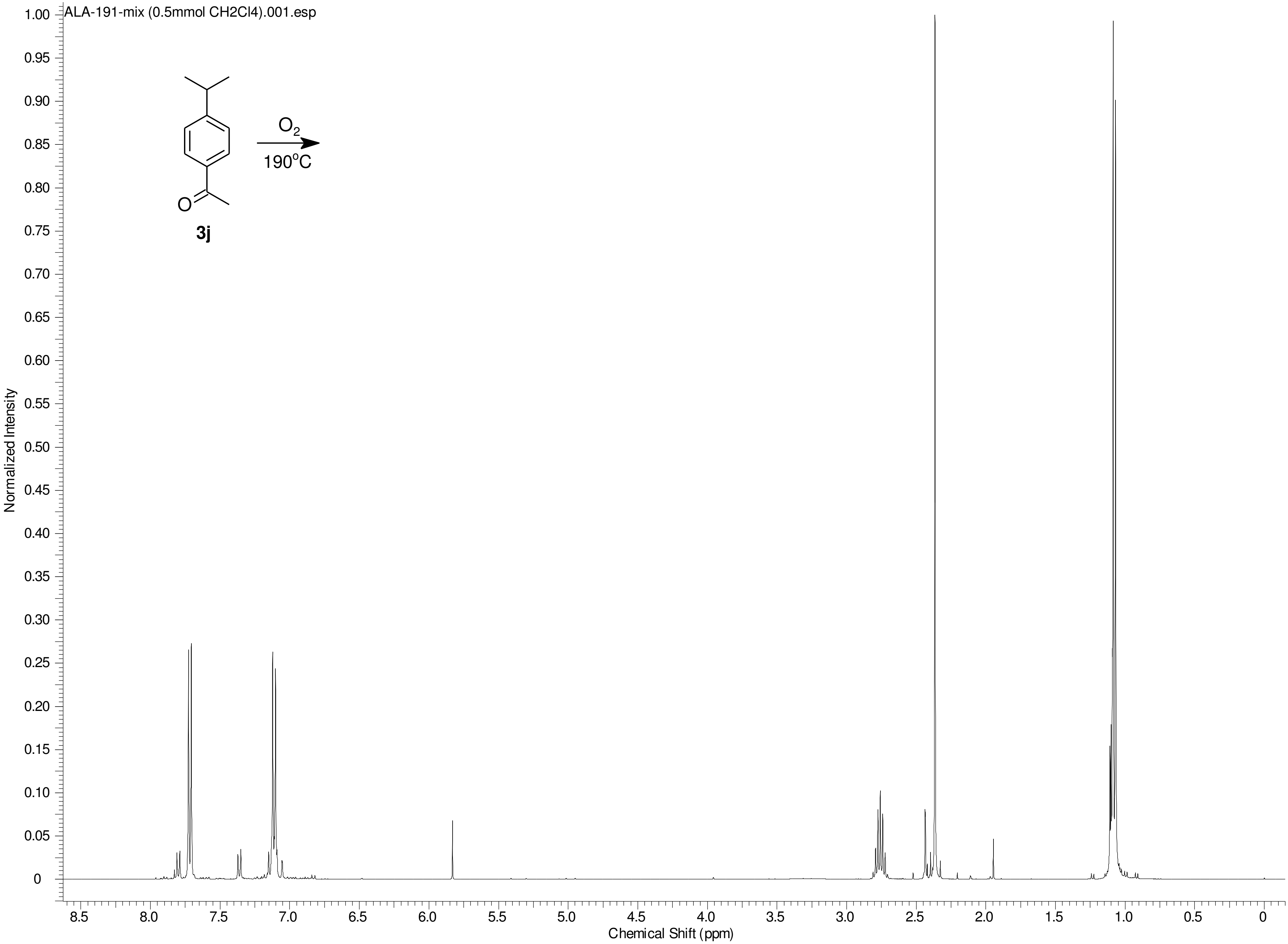
ALA-191-mix (0.5mmol CH2Cl4).001.esp
O2
190°C
3j
Normalized Intensity
Chemical Shift (ppm)

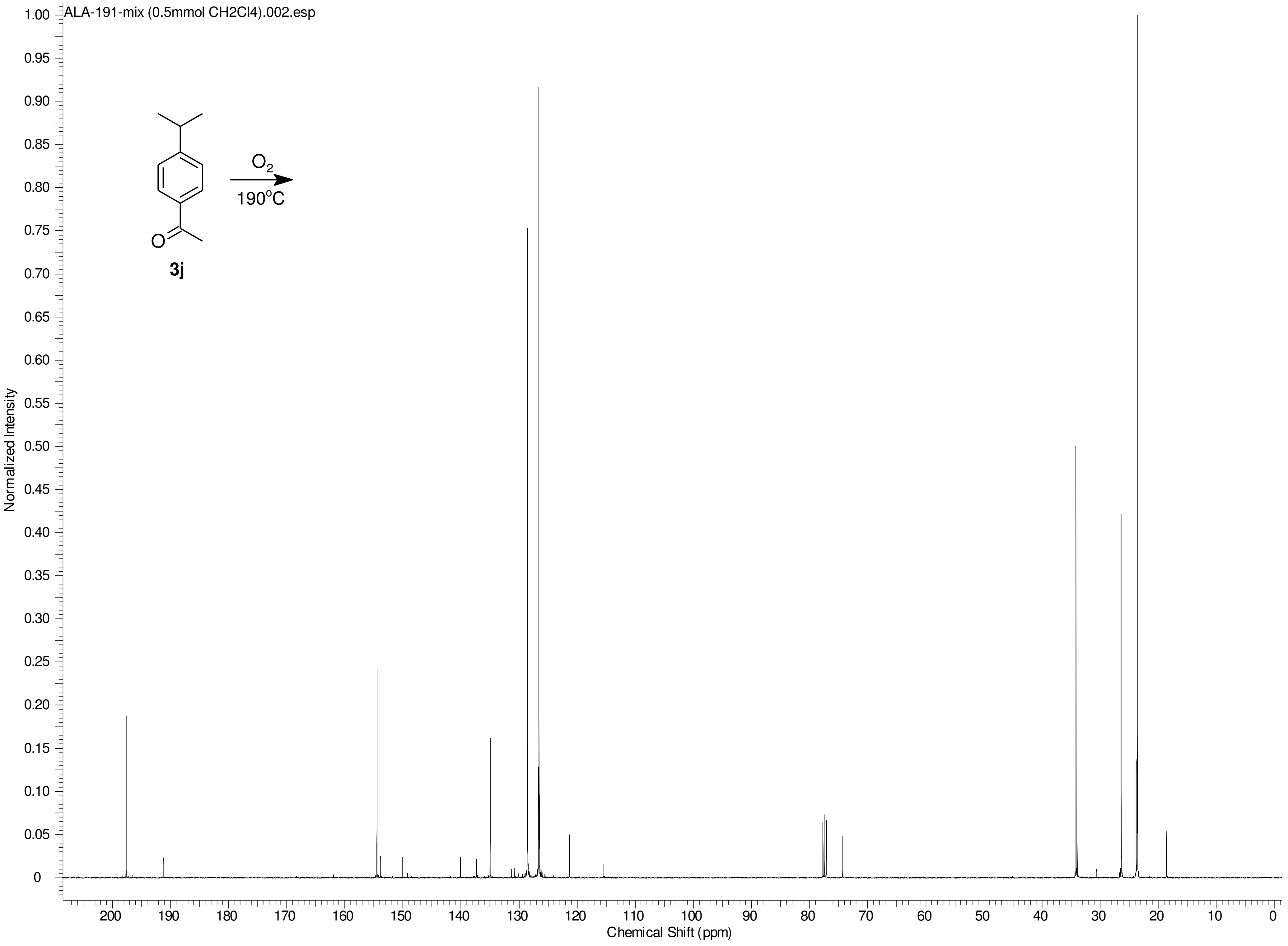
ALA-191-mix (0.5mmol CH2Cl4).002.esp
O2
190°C
3j
Normalized Intensity
Chemical Shift (ppm)

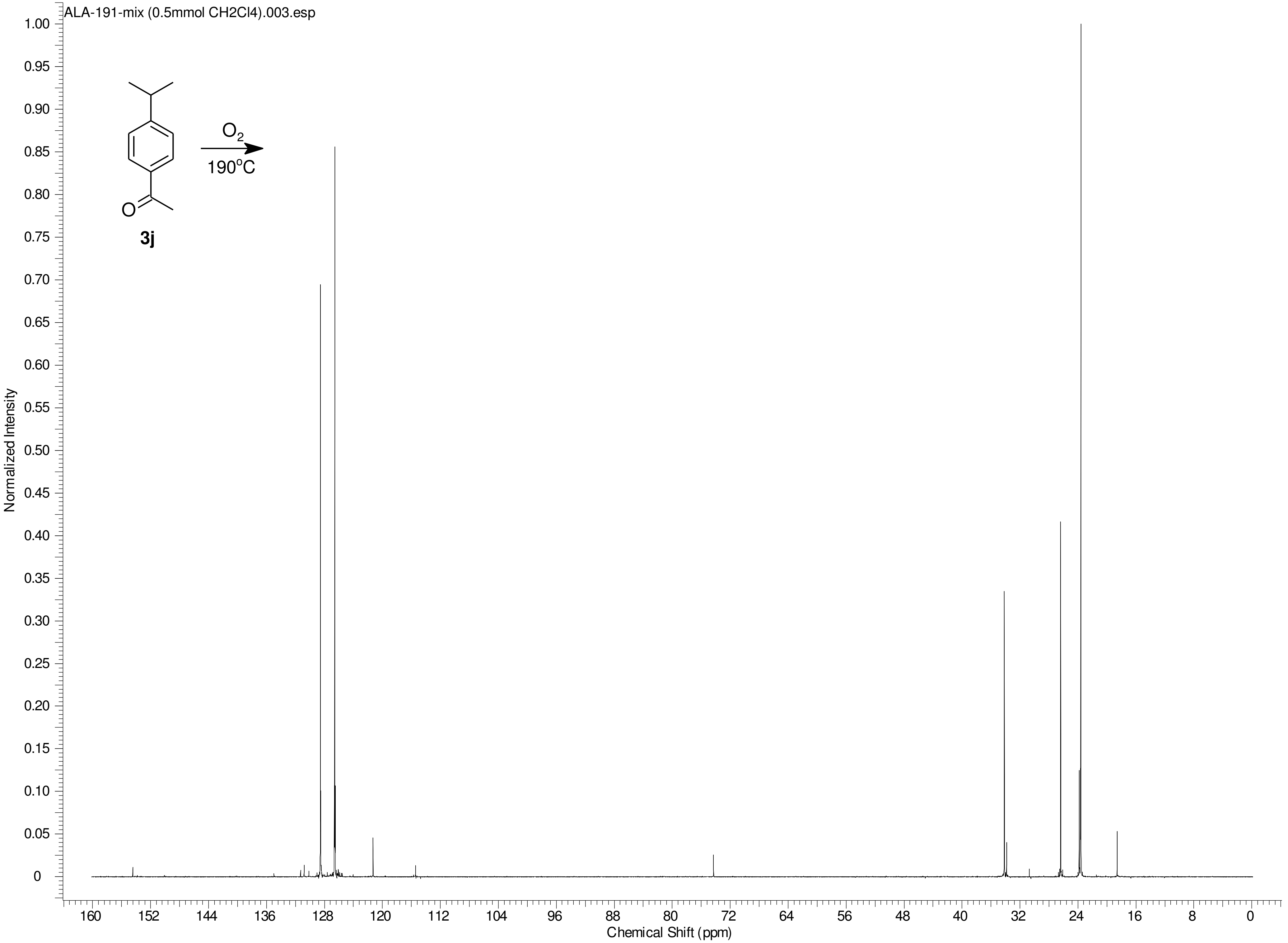
ALA-191-mix (0.5mmol CH2Cl4).003.esp
O2
190°C
3j
Normalized Intensity
Chemical Shift (ppm)
1.00
0.95
0.90
0.85
0.80
0.75
0.70
0.65
0.60
0.55
0.50
0.45
0.40
0.35
0.30
0.25
0.20
0.15
0.10
0.05
0
160
152
144
136
128
120
112
104
96
88
80
72
64
56
48
40
32
24
16
8
0

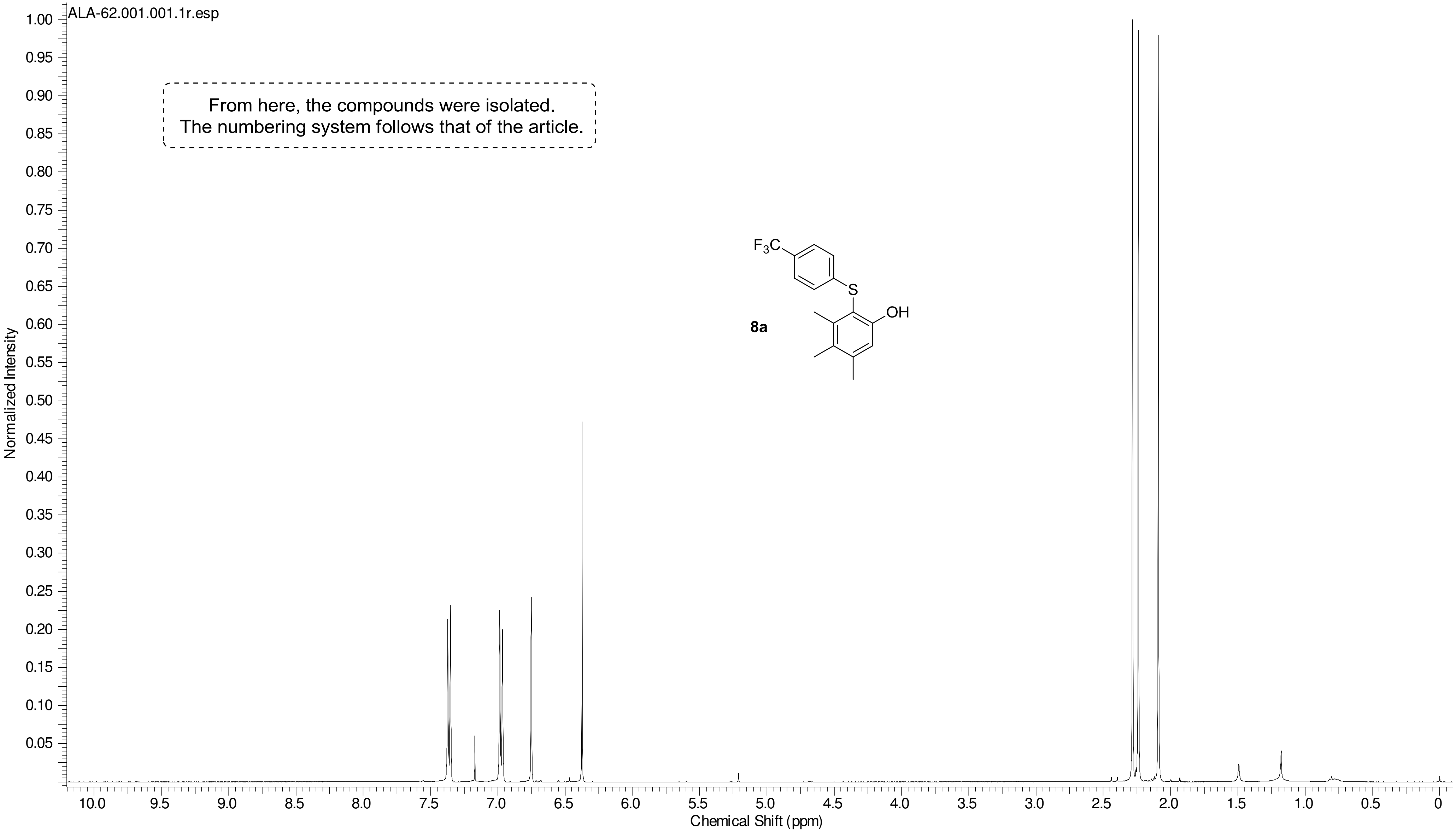
ALA-62.001.001.1r.esp
From here, the compounds were isolated.
The numbering system follows that of the article.
F3C
S
OH
8a
Normalized Intensity
Chemical Shift (ppm)

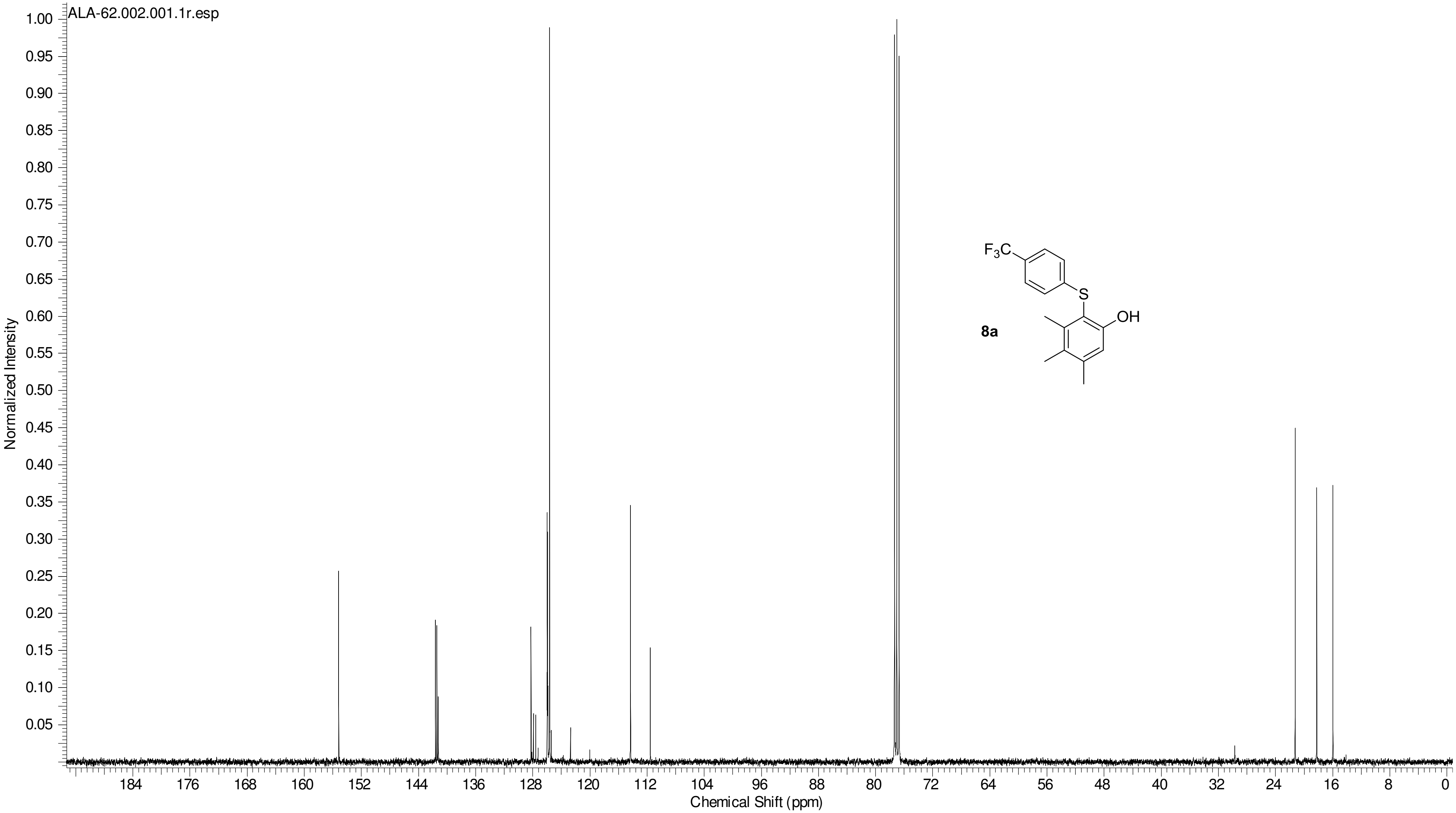
ALA-62.002.001.1r.esp
Normalized Intensity
Chemical Shift (ppm)
F3C
S
OH
8a

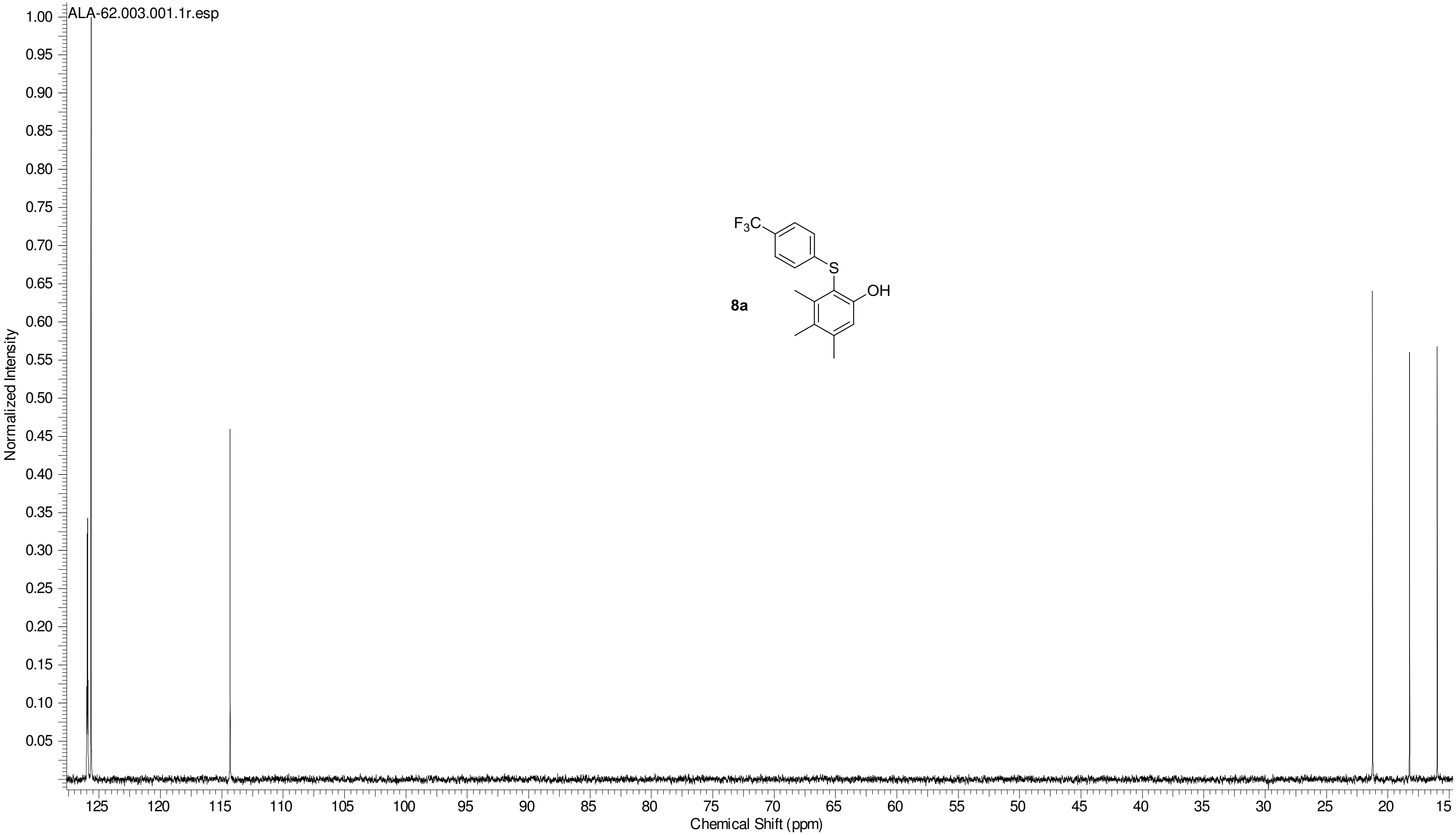
ALA-62.003.001.1r.esp
F3C
S
OH
8a
Normalized Intensity
Chemical Shift (ppm)
1.00
0.95
0.90
0.85
0.80
0.75
0.70
0.65
0.60
0.55
0.50
0.45
0.40
0.35
0.30
0.25
0.20
0.15
0.10
0.05
125
120
115
110
105
100
95
90
85
80
75
70
65
60
55
50
45
40
35
30
25
20
15

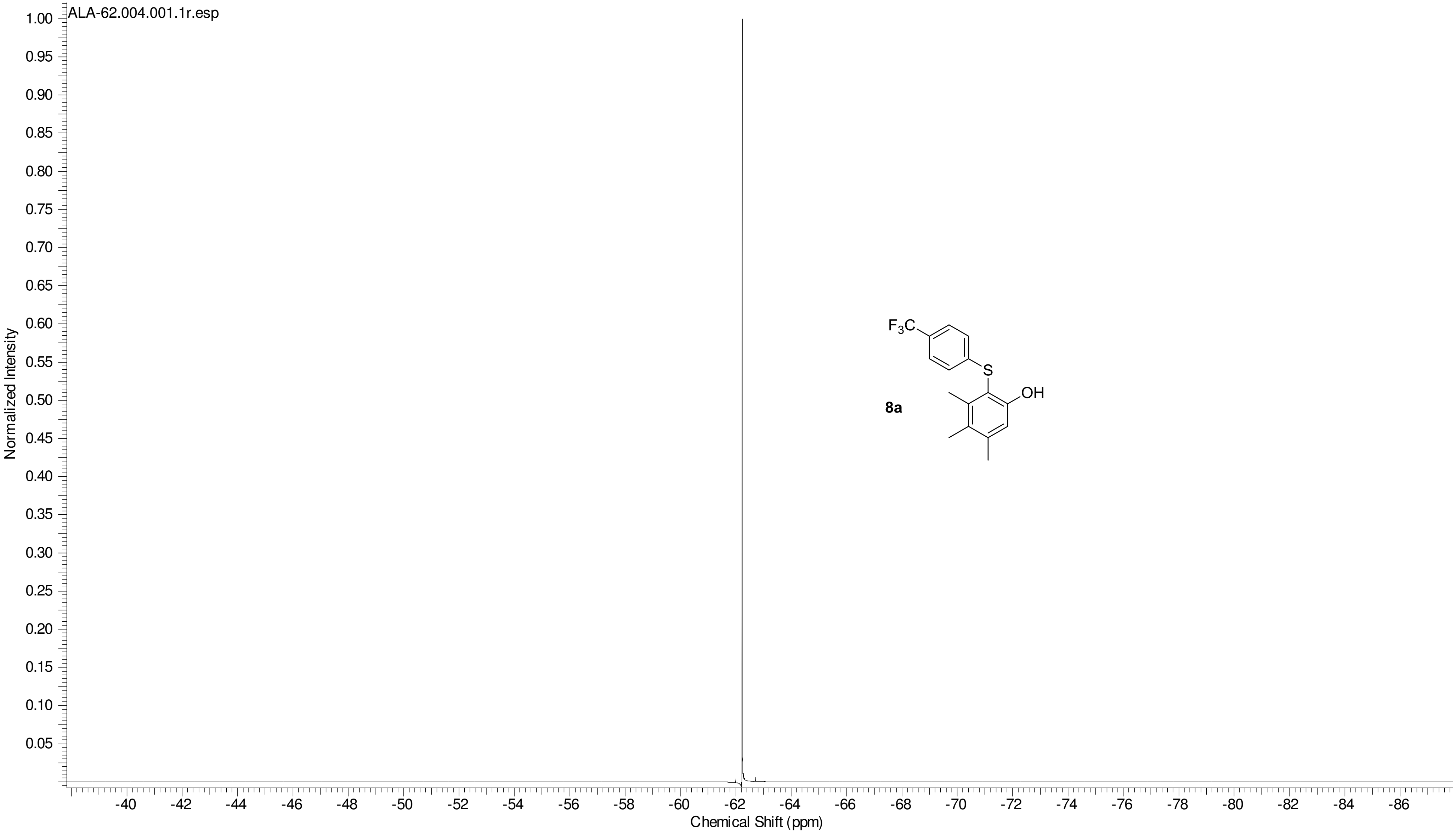
ALA-62.004.001.1r.esp
Normalized Intensity
Chemical Shift (ppm)
F3C
S
OH

8a

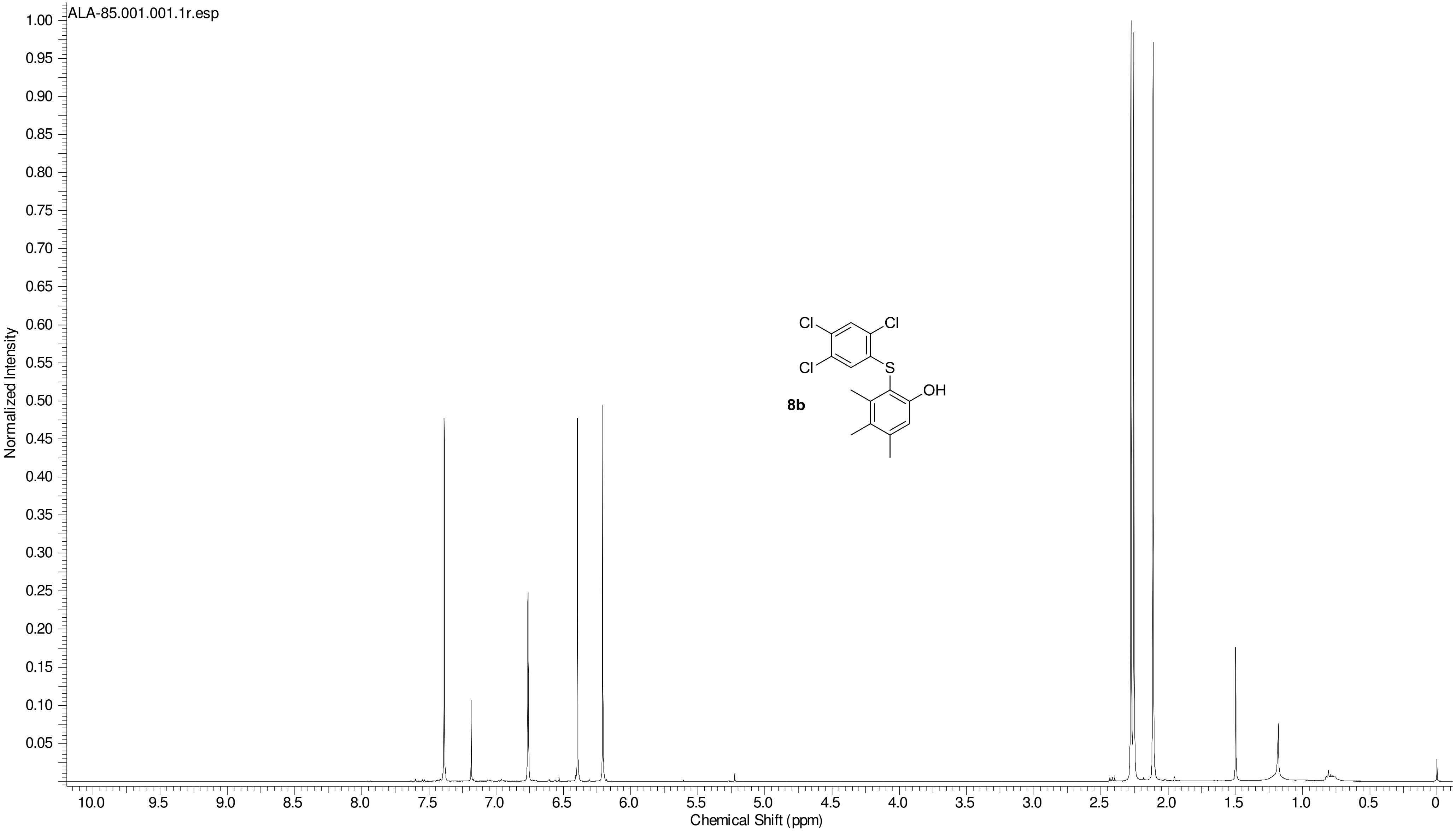
ALA-85.001.001.1r.esp
Normalized Intensity
Chemical Shift (ppm)
Cl
Cl
Cl
S
OH
8b

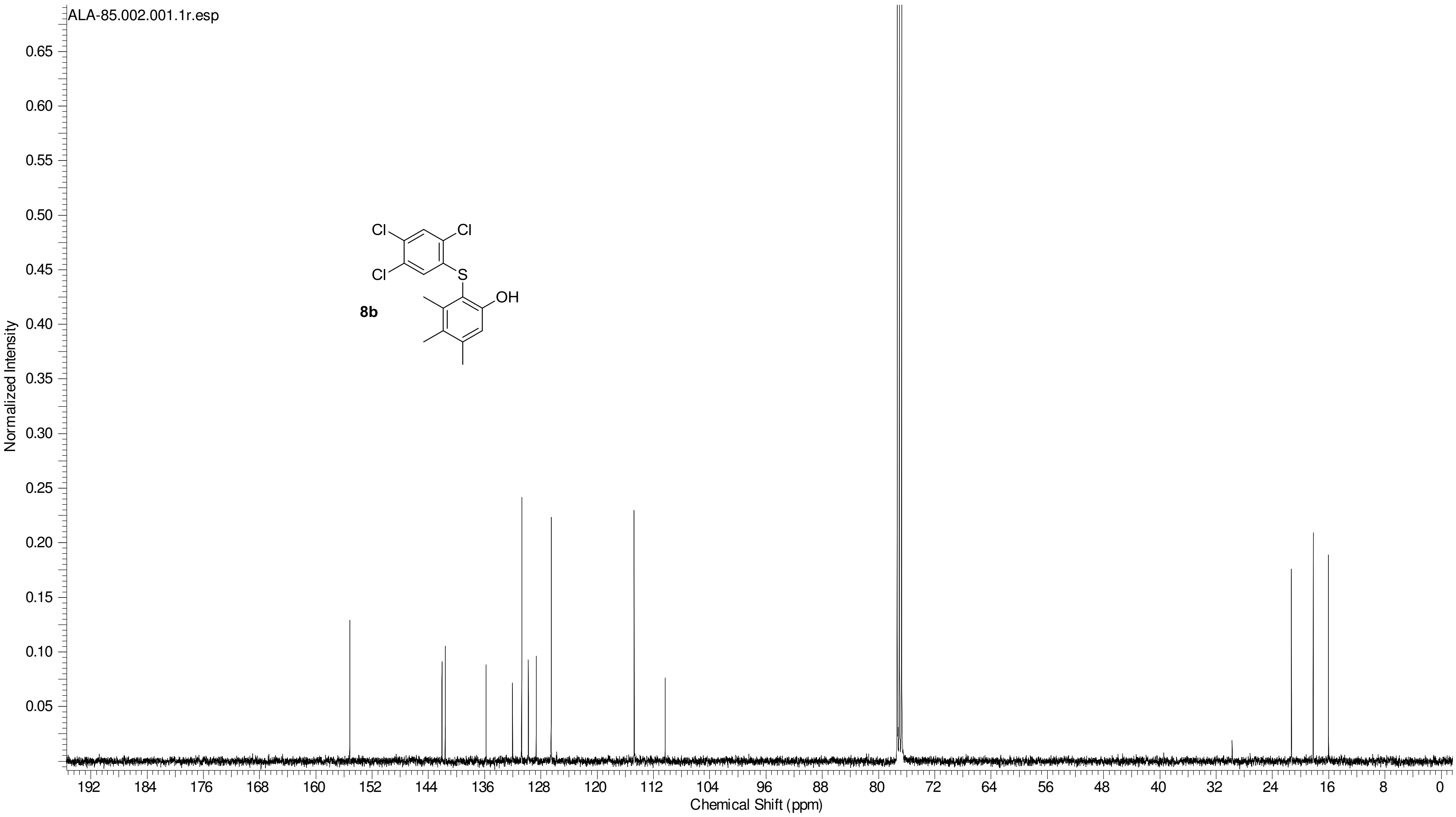
ALA-85.002.001.1r.esp
Cl
Cl
Cl
S
OH
8b
Normalized Intensity
0.65
0.60
0.55
0.50
0.45
0.40
0.35
0.30
0.25
0.20
0.15
0.10
0.05
192
184
176
168
160
152
144
136
128
120
112
104
96
88
80
72
64
56
48
40
32
24
16
8
0
Chemical Shift (ppm)

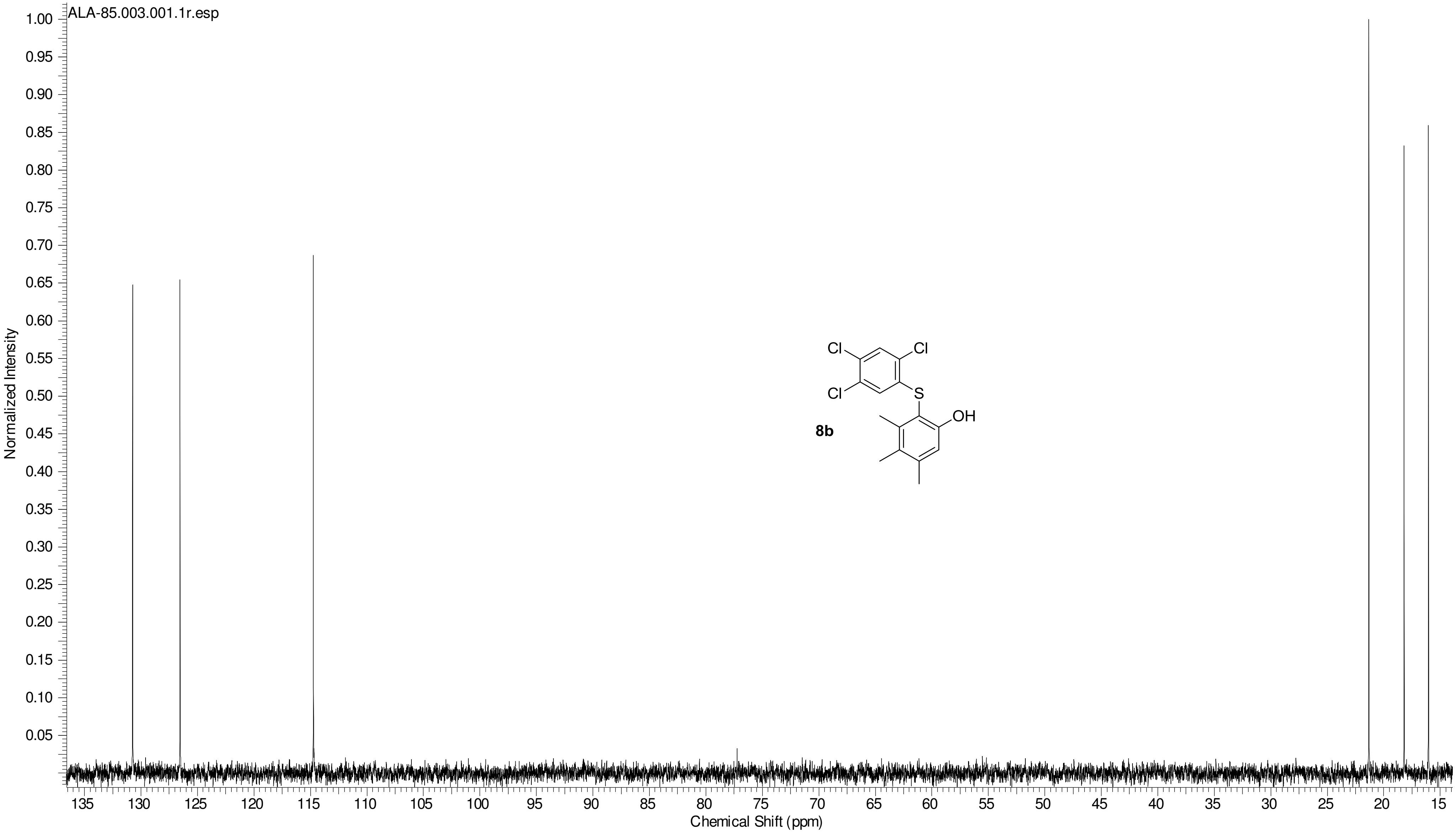
ALA-85.003.001.1r.esp
Normalized Intensity
Chemical Shift (ppm)
Cl
Cl
Cl
S
OH
8b

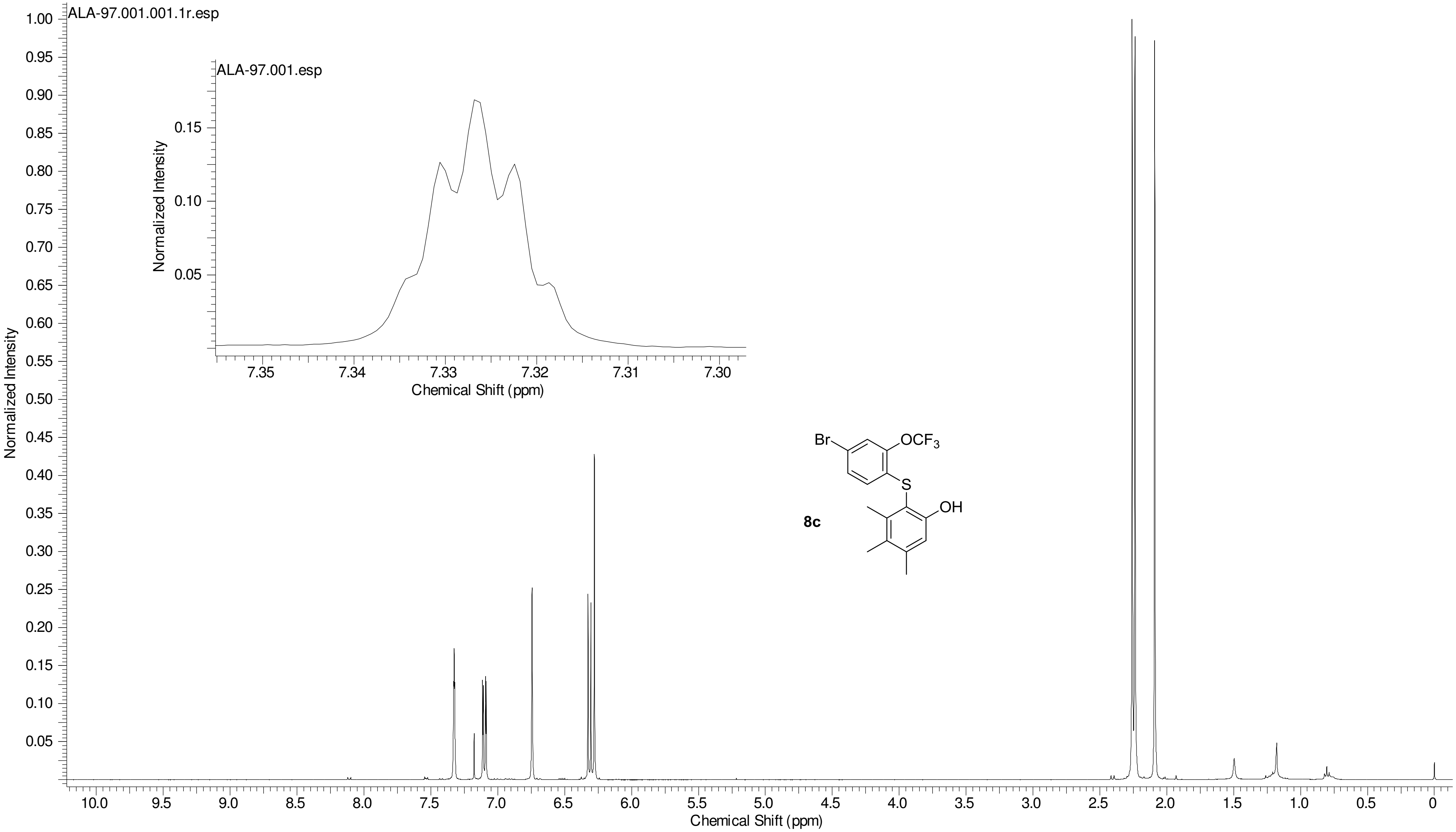
ALA-97.001.001.1r.esp
ALA-97.001.esp
Normalized Intensity
Chemical Shift (ppm)
Br
OCF3
S
OH
8c

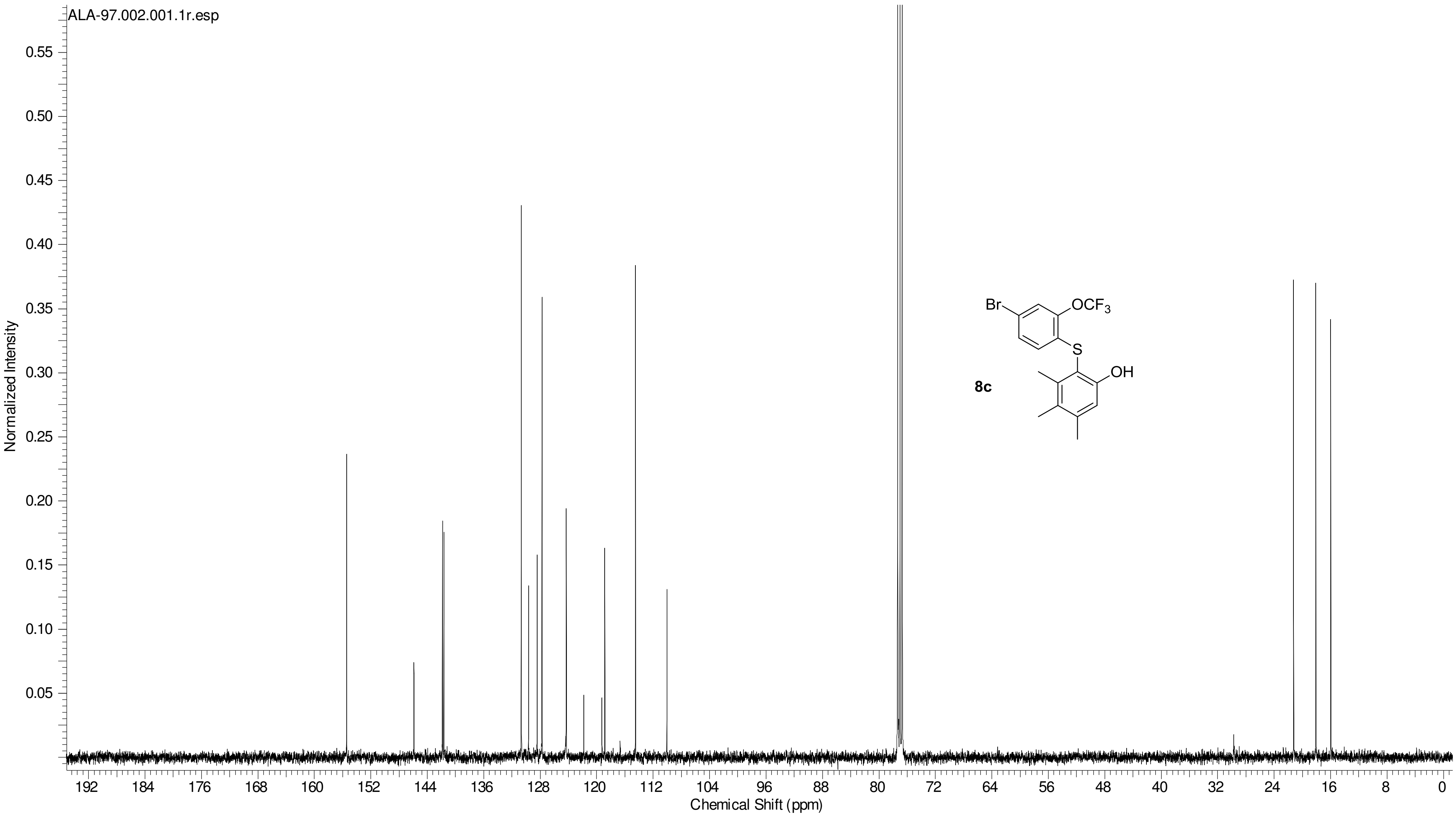

ALA-97.002.001.1r.esp
Normalized Intensity
Chemical Shift (ppm)
Br
OCF3
S
OH
8c

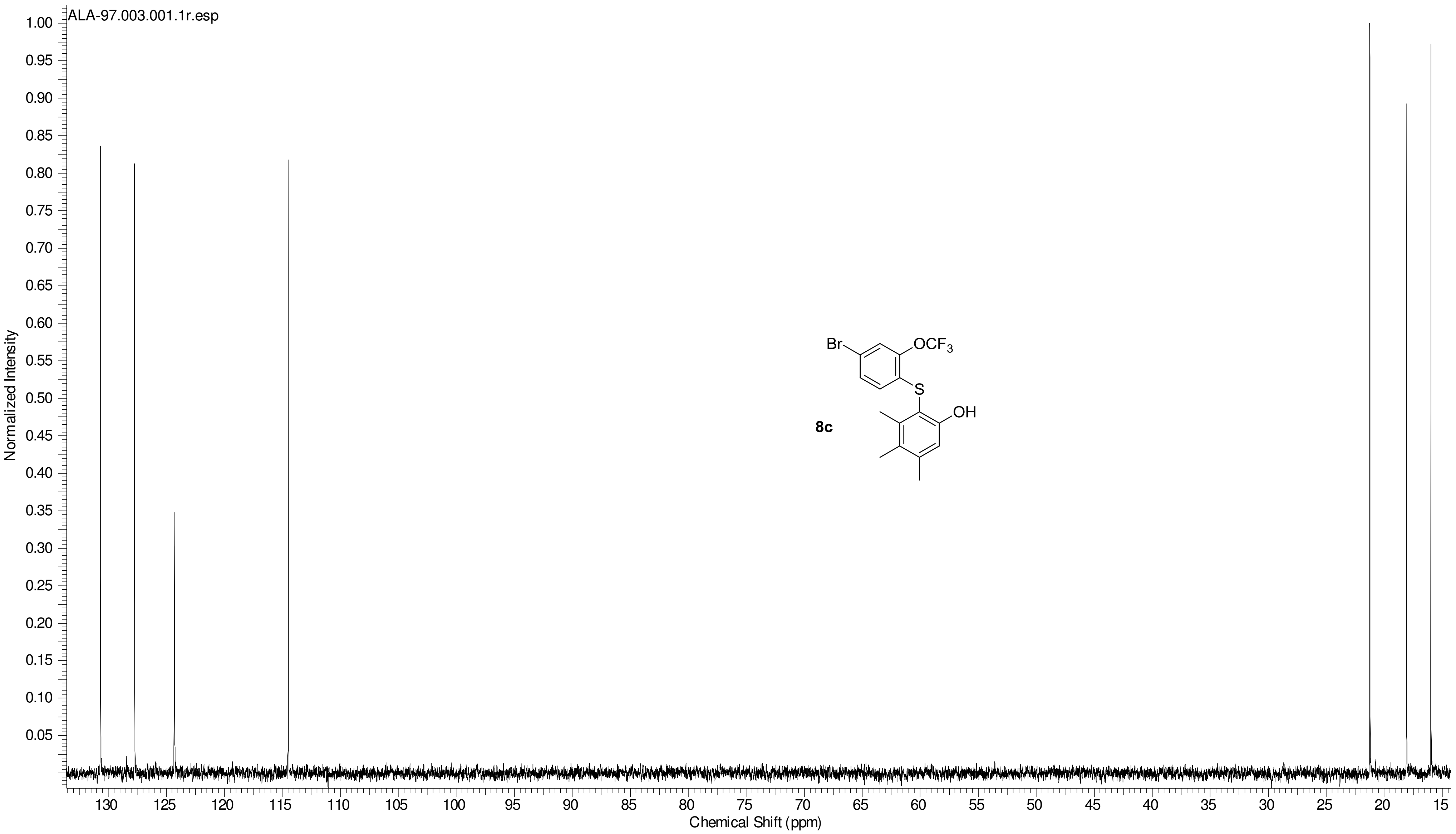

ALA-97.003.001.1r.esp
Normalized Intensity
Chemical Shift (ppm)
Br
OCF3
S
OH
8c

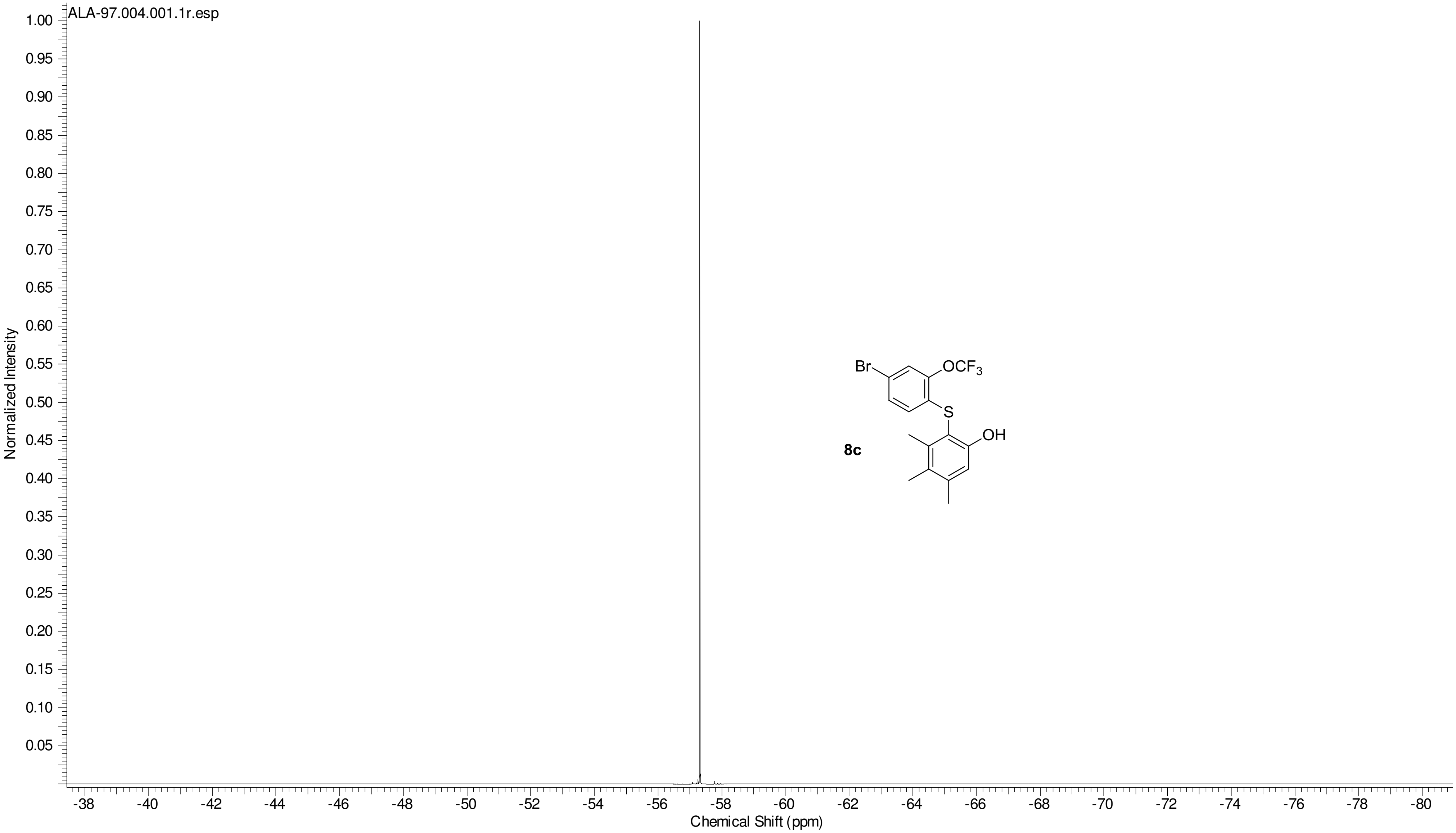
ALA-97.004.001.1r.esp
Normalized Intensity
Chemical Shift (ppm)
Br
OCF3
S
OH
8c

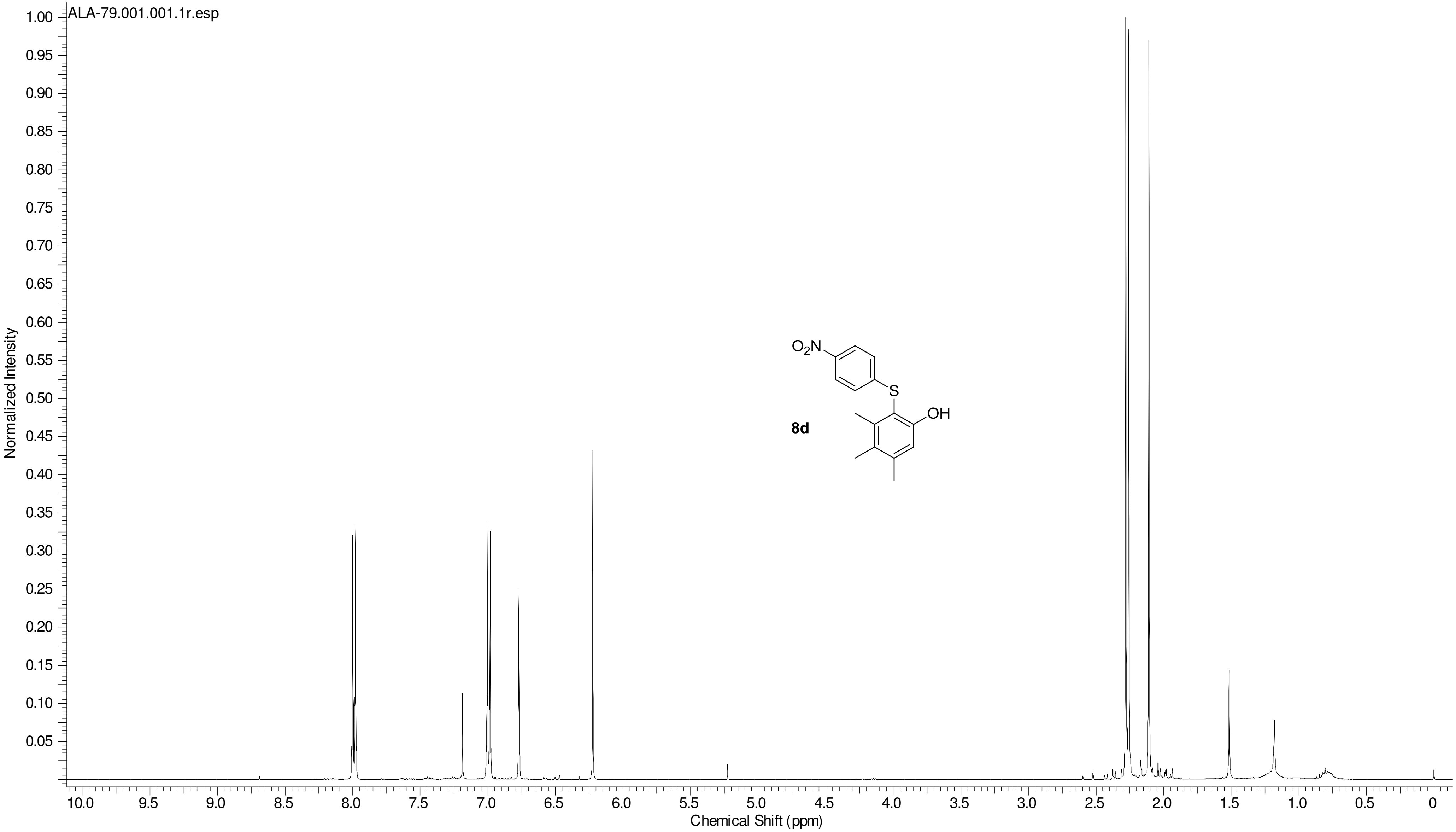

ALA-79.001.001.1r.esp
Normalized Intensity
Chemical Shift (ppm)
O2N
S
OH
8d

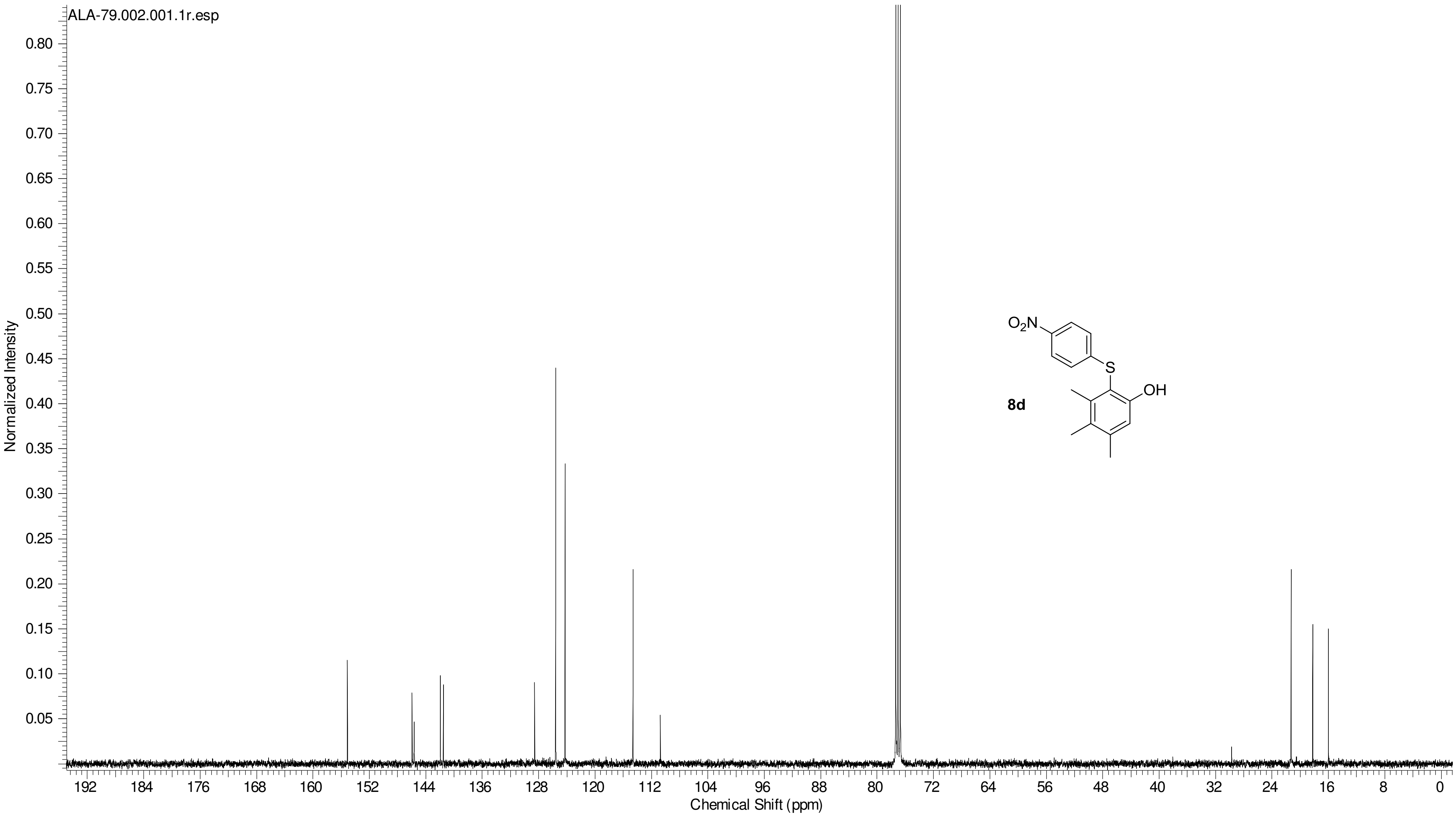
ALA-79.002.001.1r.esp
Normalized Intensity
Chemical Shift (ppm)
O_2N
S
OH
8d

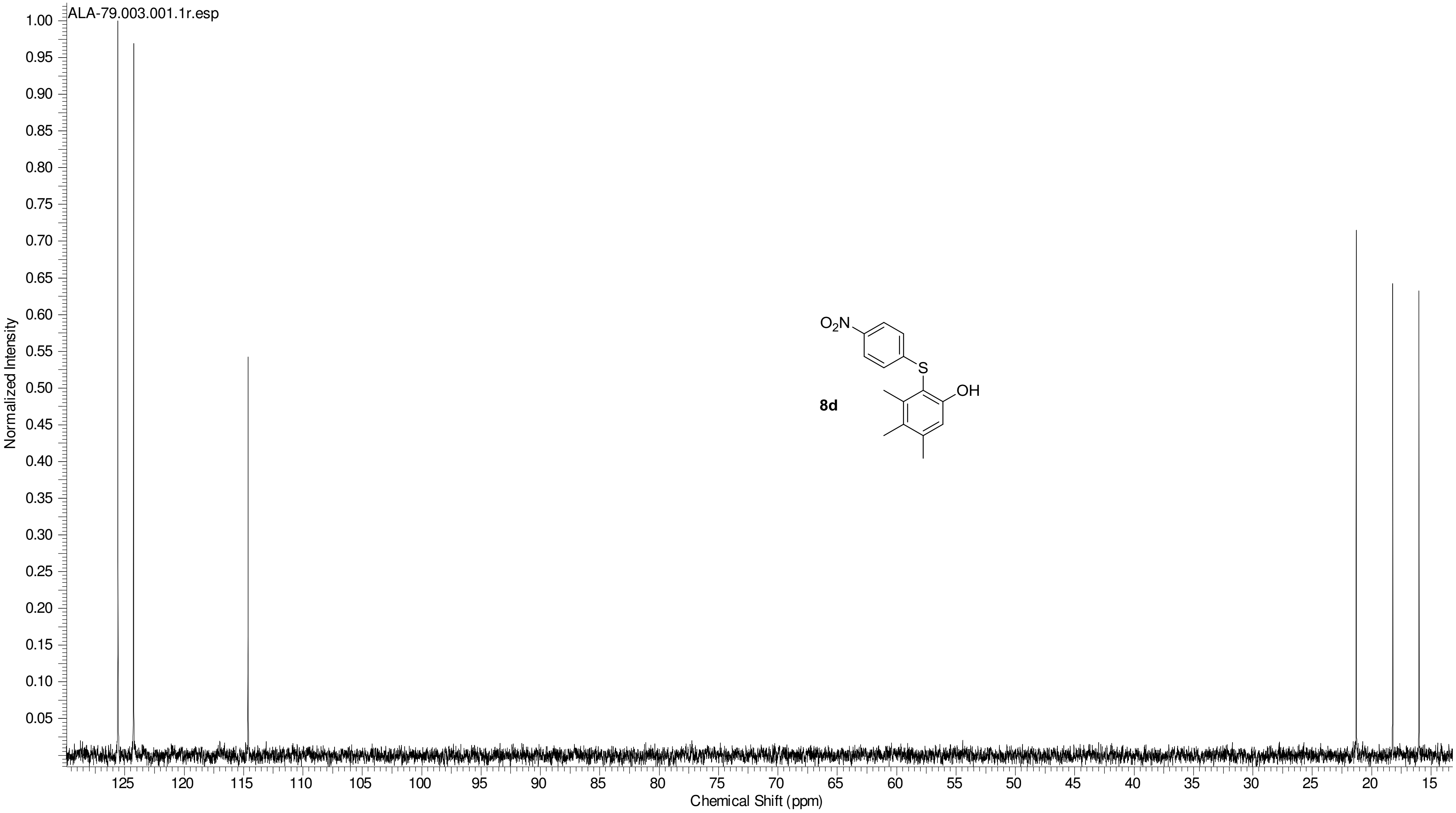
ALA-79.003.001.1r.esp
Normalized Intensity
Chemical Shift (ppm)
O2N
S
OH
8d

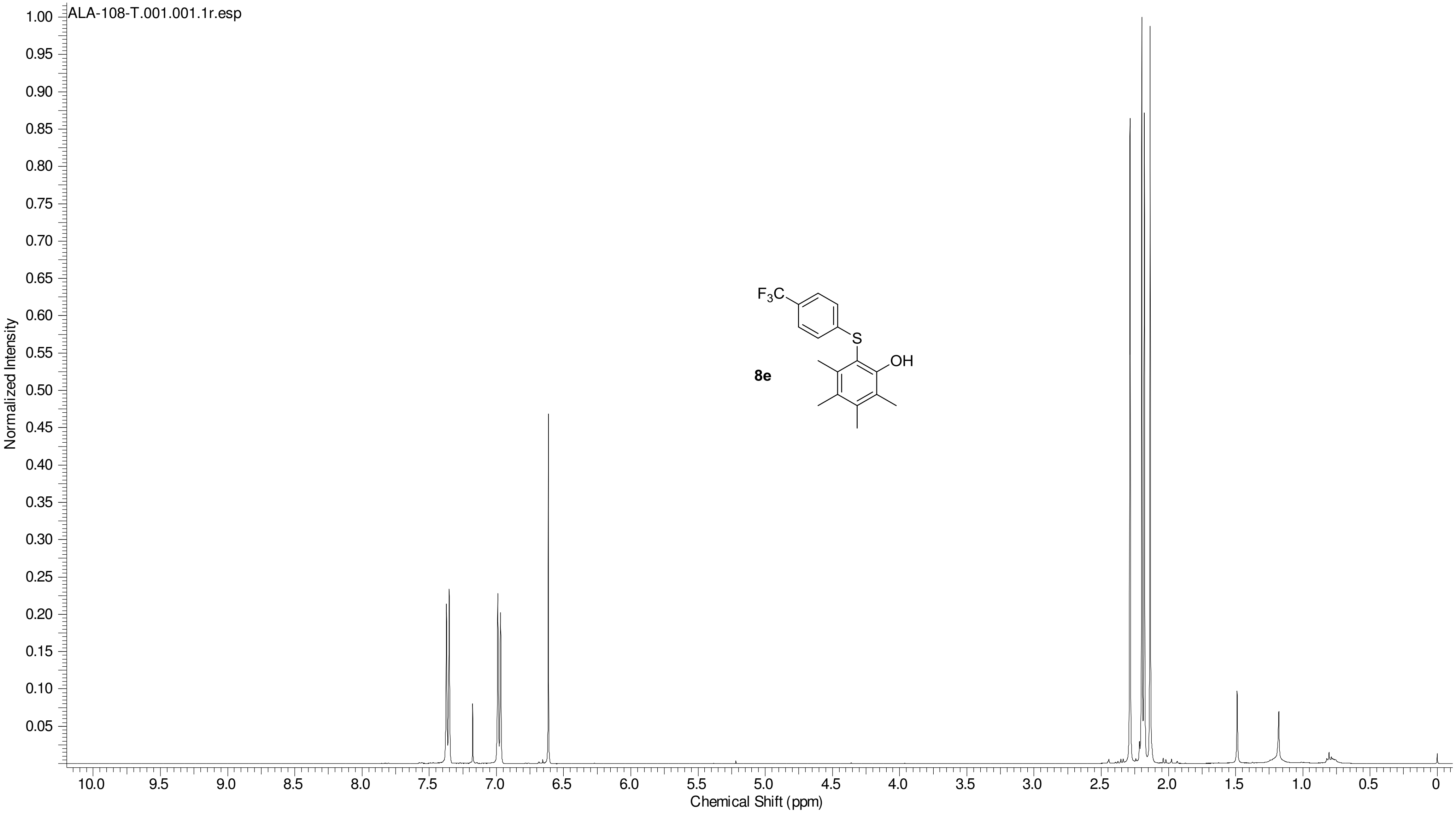

ALA-108-T.001.001.1r.esp
Normalized Intensity
1.00
0.95
0.90
0.85
0.80
0.75
0.70
0.65
0.60
0.55
0.50
0.45
0.40
0.35
0.30
0.25
0.20
0.15
0.10
0.05
F3C
S
OH
8e
10.0
9.5
9.0
8.5
8.0
7.5
7.0
6.5
6.0
5.5
5.0
4.5
4.0
3.5
3.0
2.5
2.0
1.5
1.0
0.5
0
Chemical Shift (ppm)

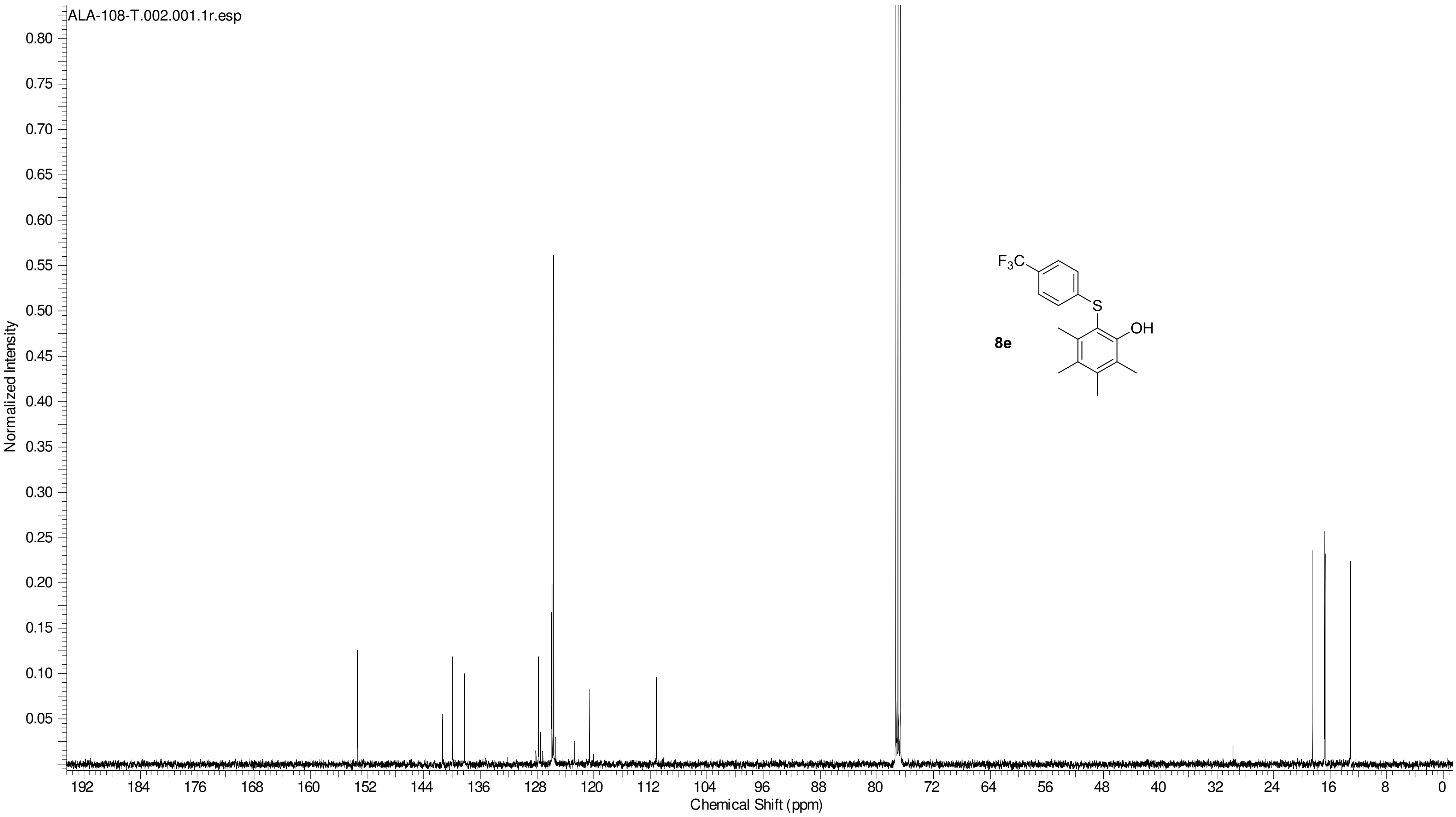

ALA-108-T.002.001.1r.esp
Normalized Intensity
Chemical Shift (ppm)
F3C
S
OH
8e

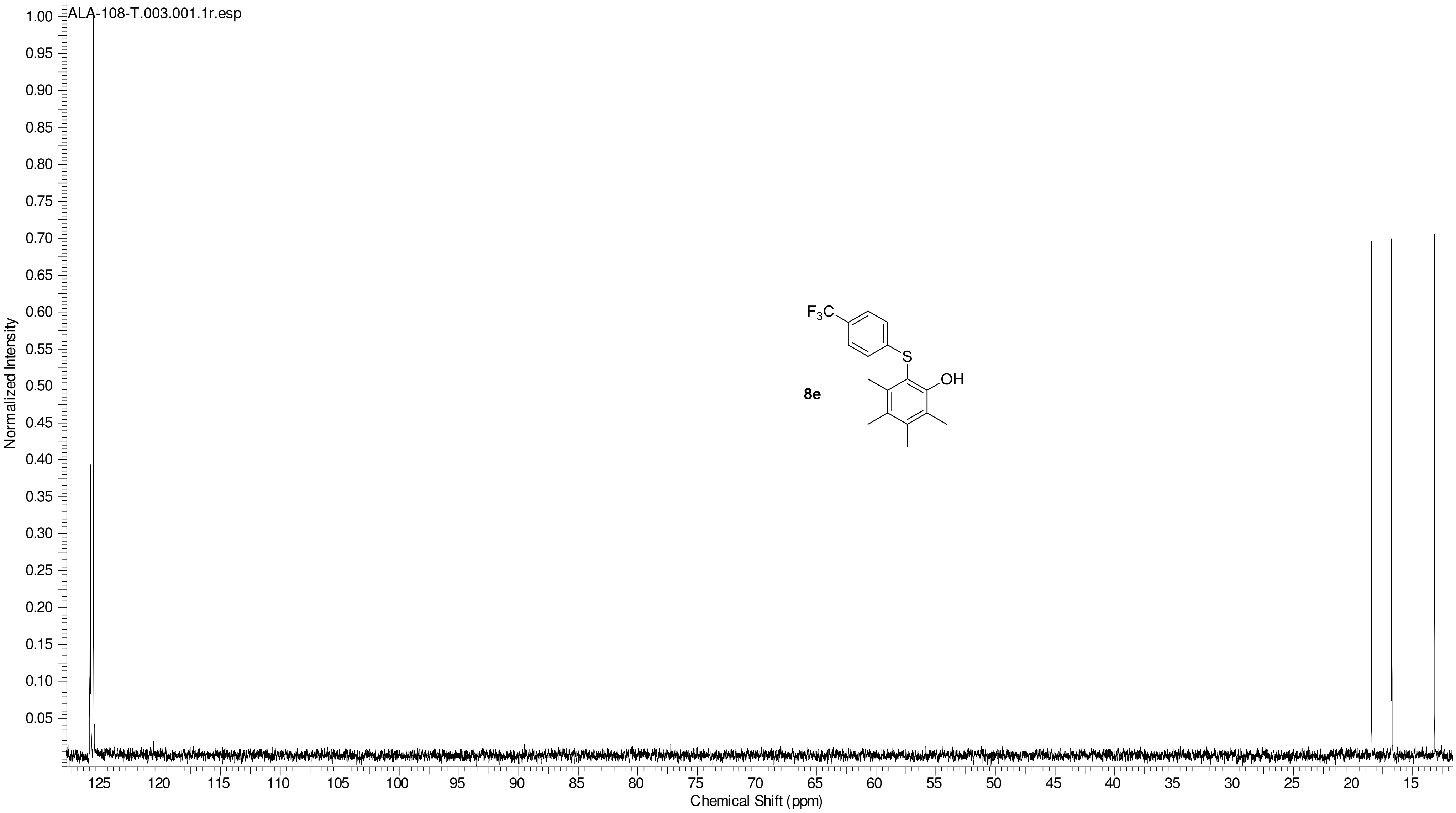
ALA-108-T.003.001.1r.esp
Normalized Intensity
Chemical Shift (ppm)
F3C
S
OH
8e

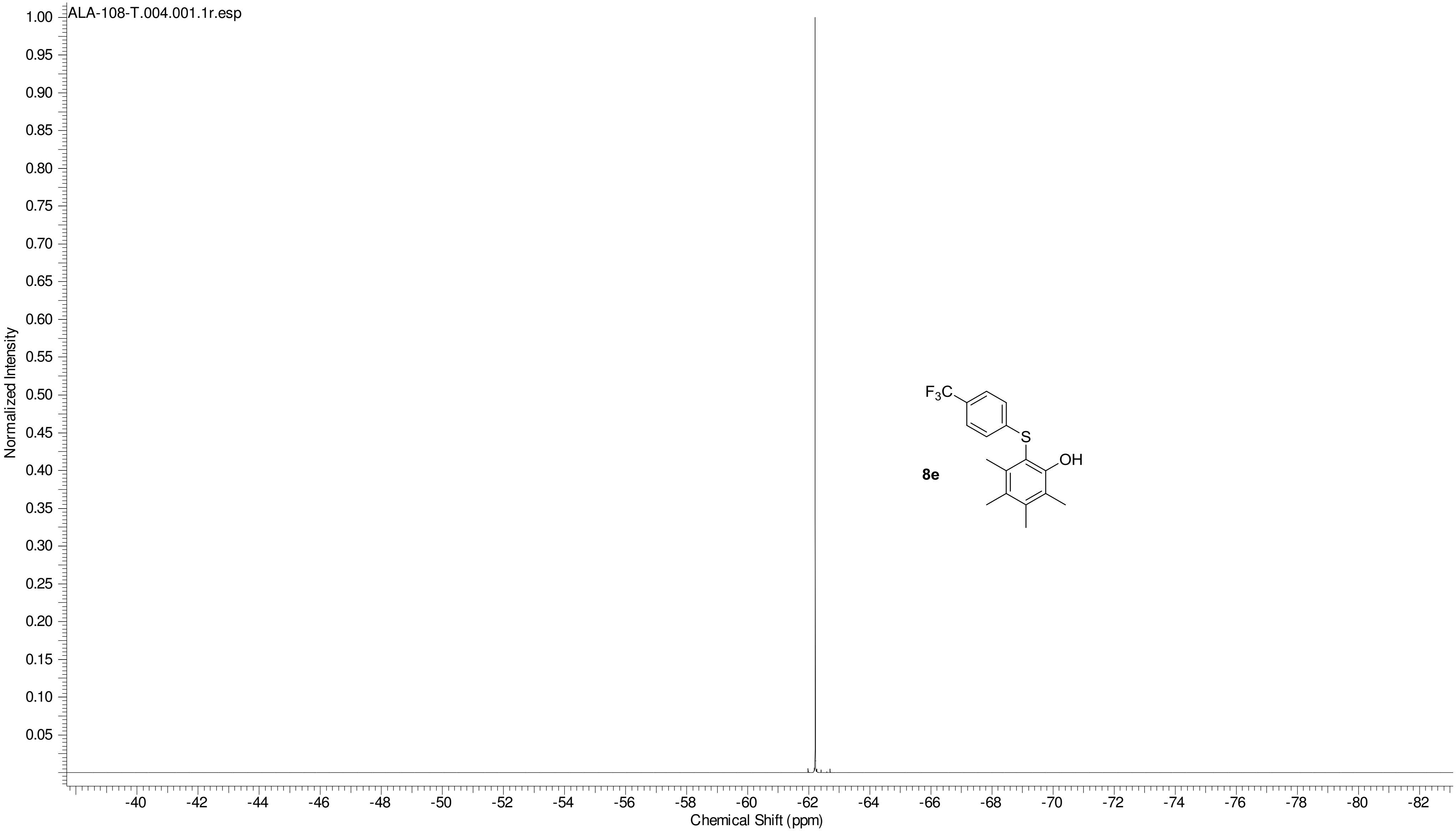
ALA-108-T.004.001.1r.esp
Normalized Intensity
Chemical Shift (ppm)
F3C
S
OH
8e

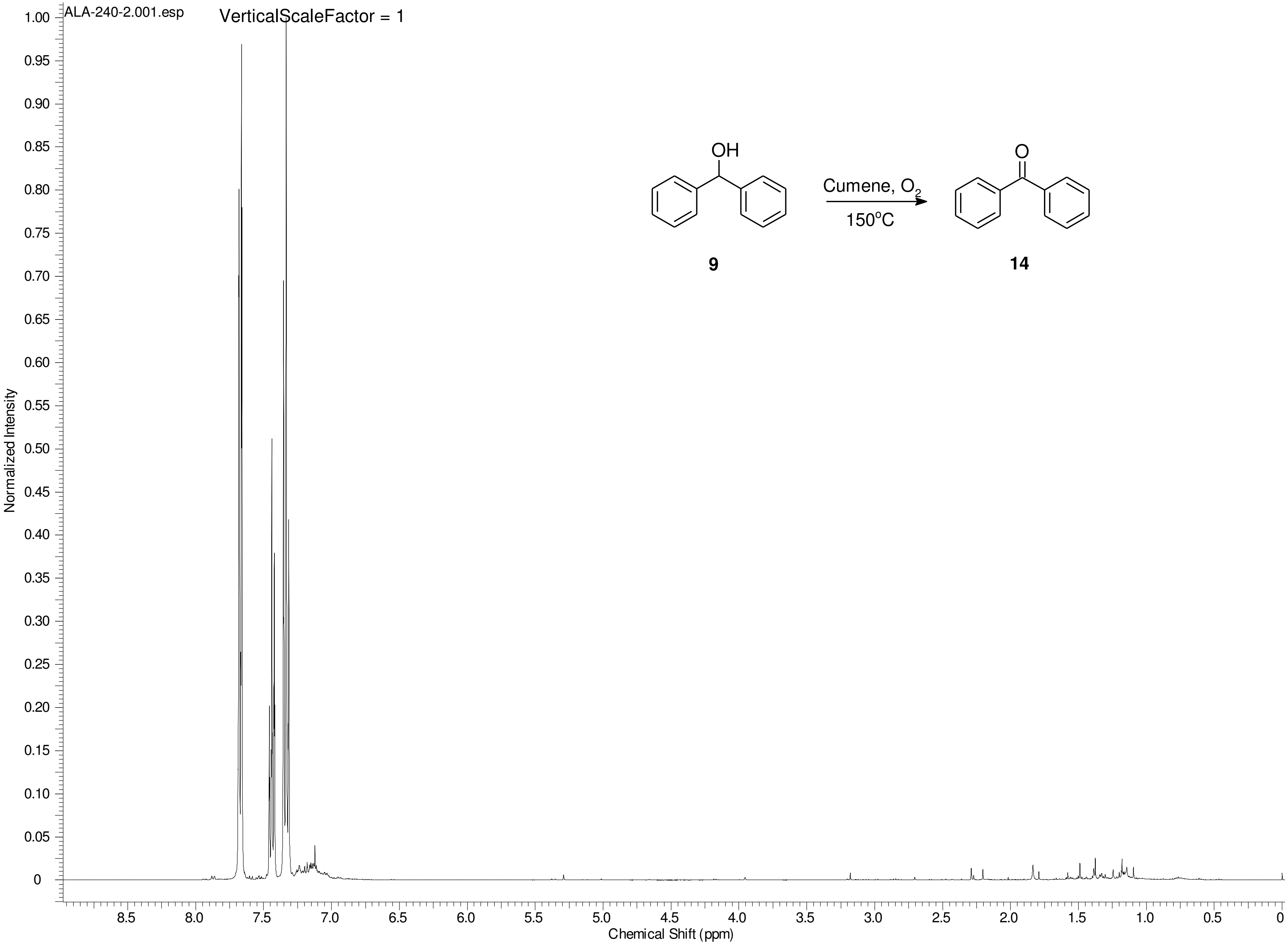
ALA-240-2.001.esp
VerticalScaleFactor = 1
OH
Cumene, O2
150°C
O
9
14
Normalized Intensity
Chemical Shift (ppm)

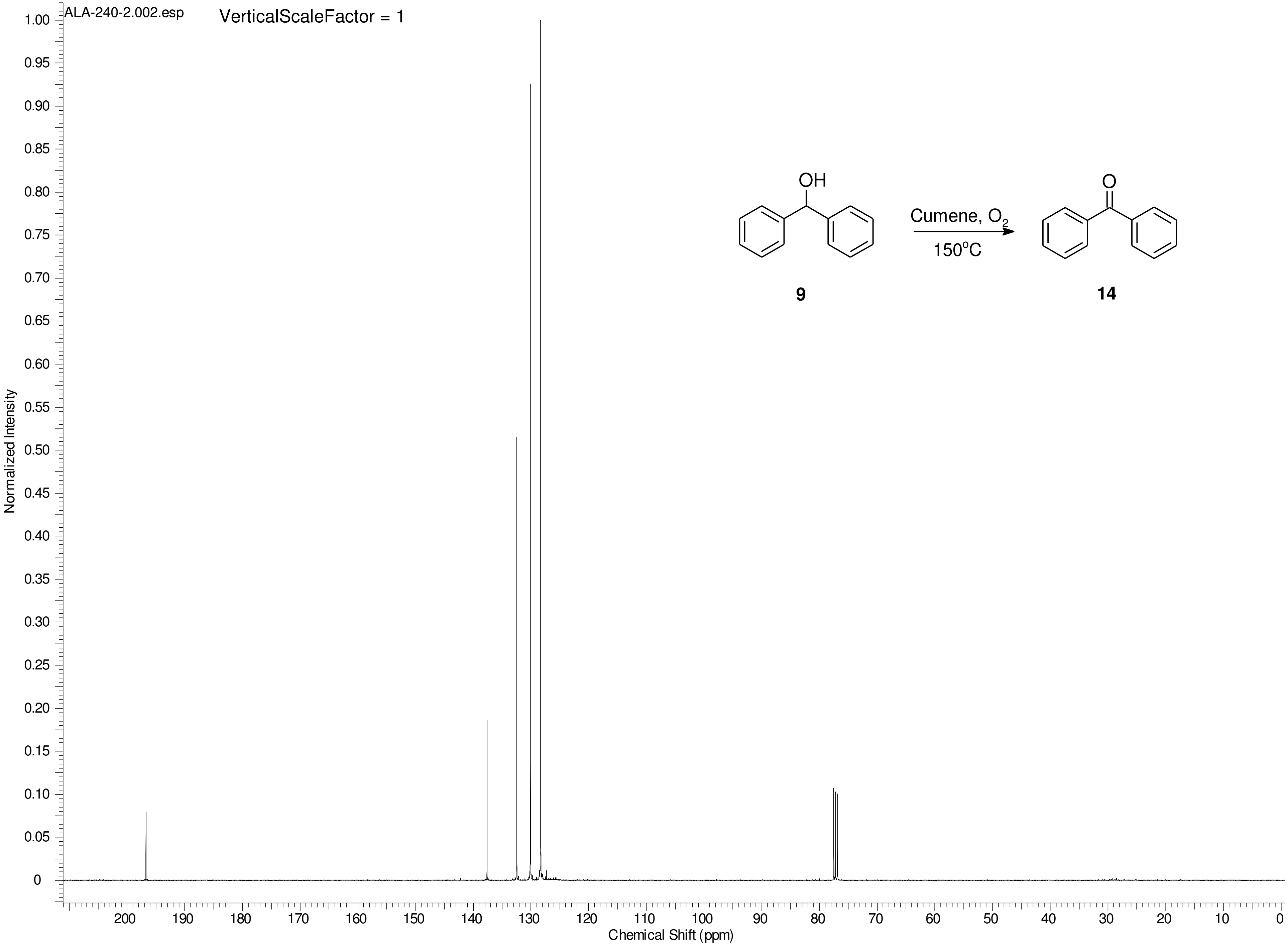

ALA-240-2.002.esp
VerticalScaleFactor = 1
OH
Cumene, O_2
150°C
O
9
14
Normalized Intensity
Chemical Shift (ppm)

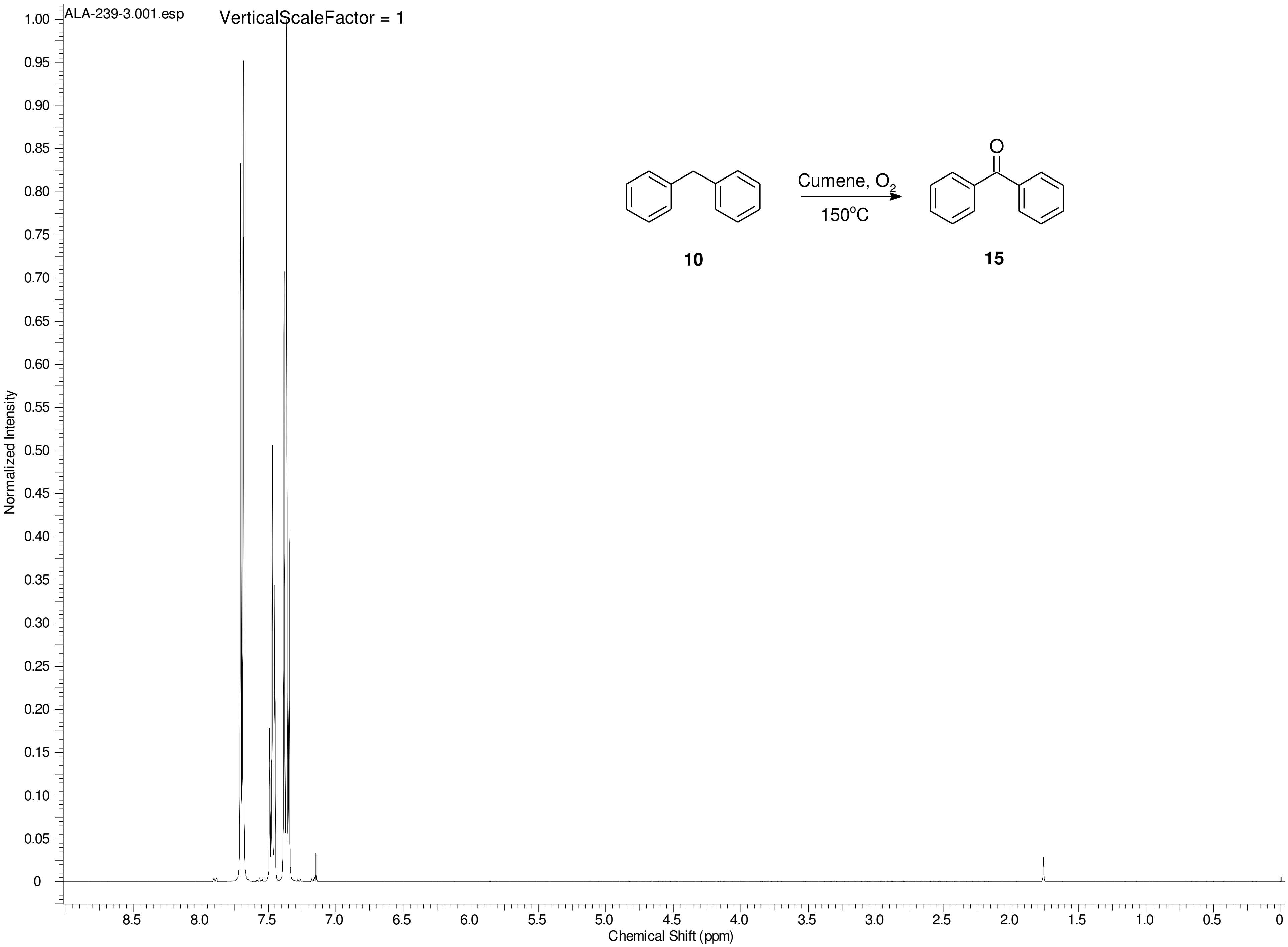
ALA-239-3.001.esp
VerticalScaleFactor = 1
Cumene, O_2
150°C
10
15
Normalized Intensity
Chemical Shift (ppm)

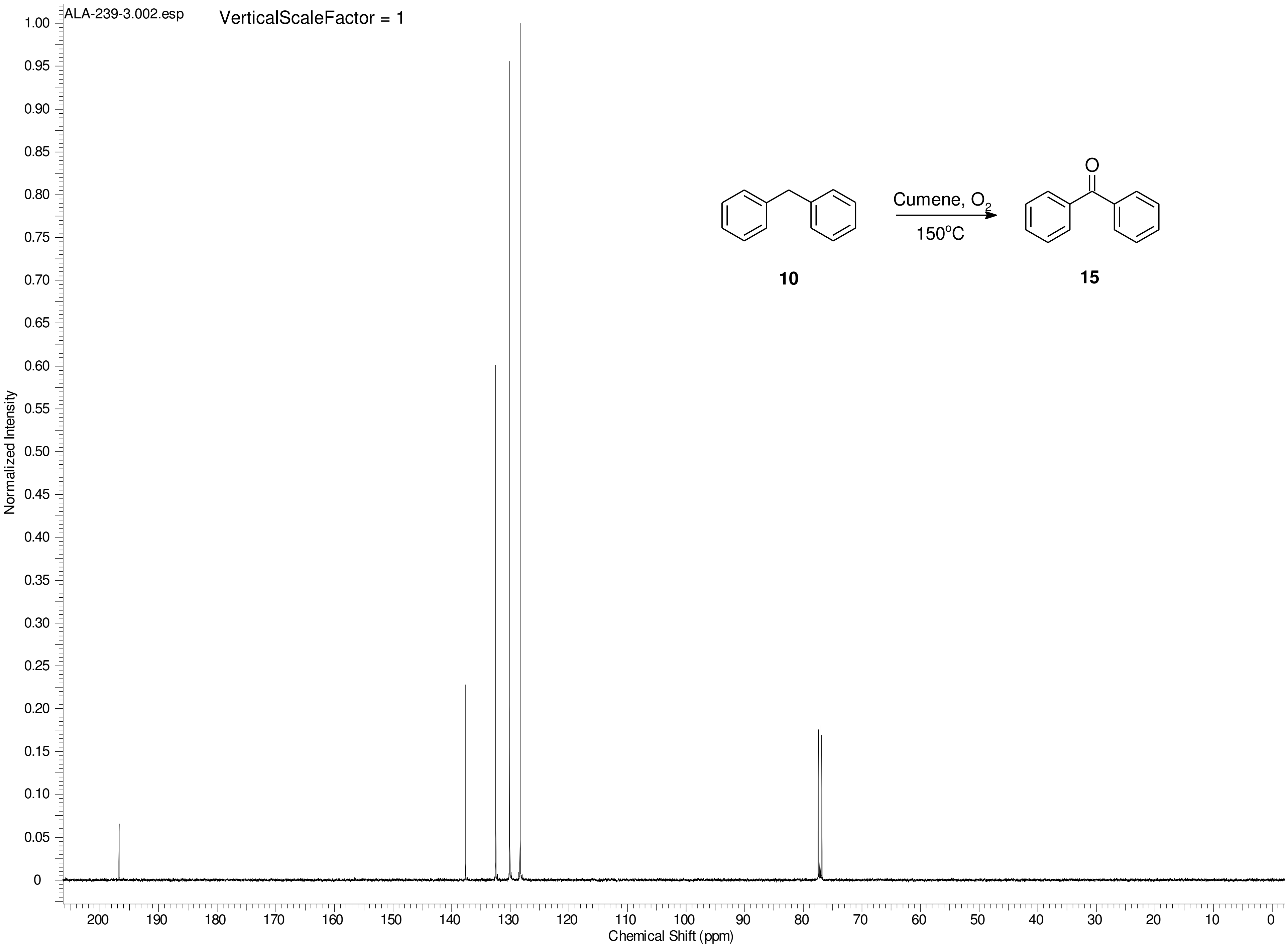

ALA-239-3.002.esp
VerticalScaleFactor = 1
Cumene, O_2
150°C
10
15
Normalized Intensity
Chemical Shift (ppm)

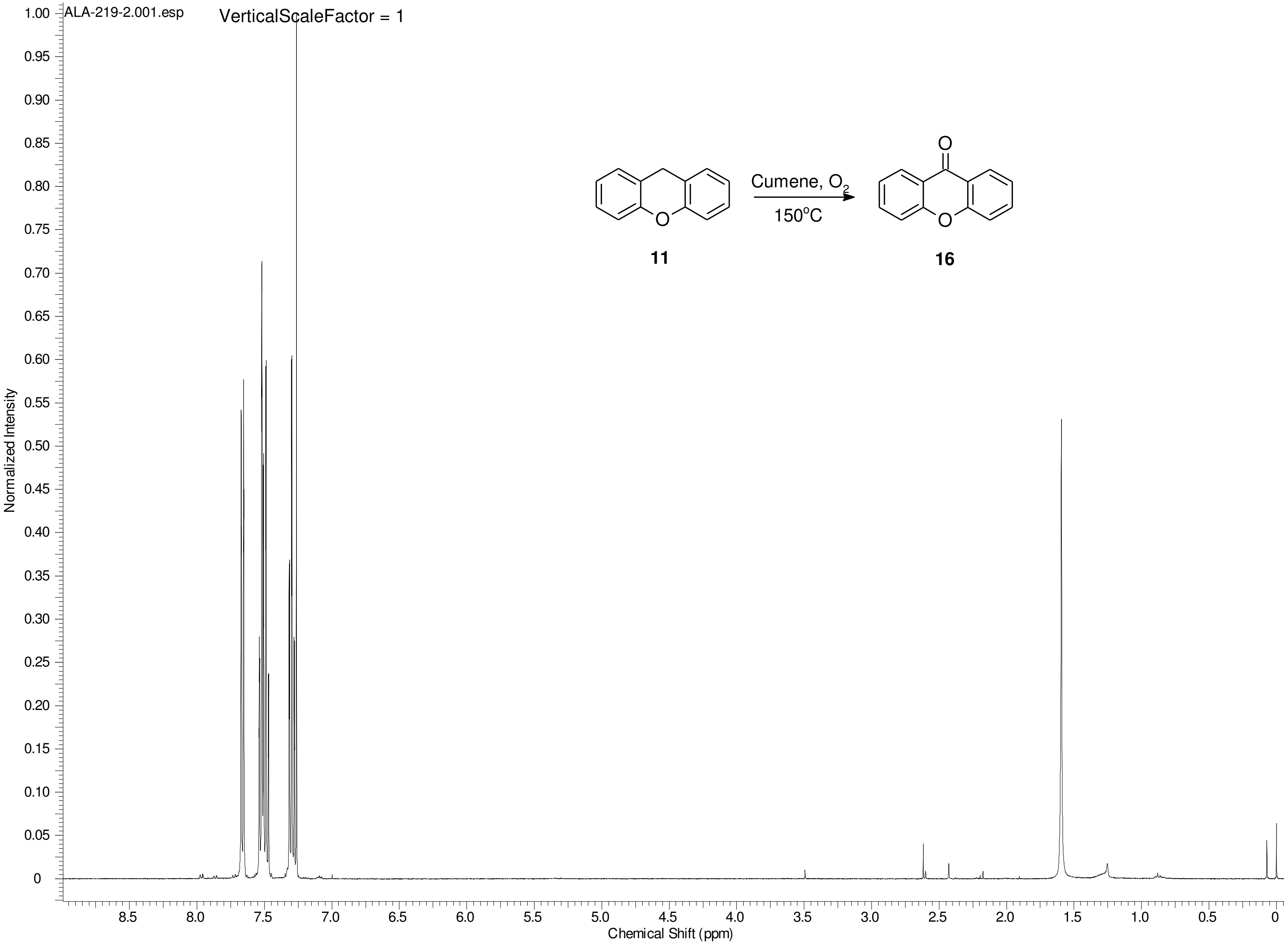
ALA-219-2.001.esp
VerticalScaleFactor = 1
Normalized Intensity
1.00
0.95
0.90
0.85
0.80
0.75
0.70
0.65
0.60
0.55
0.50
0.45
0.40
0.35
0.30
0.25
0.20
0.15
0.10
0.05
0
Cumene, O_2
150°C
11
16
8.5
8.0
7.5
7.0
6.5
6.0
5.5
5.0
4.5
4.0
3.5
3.0
2.5
2.0
1.5
1.0
0.5
0
Chemical Shift (ppm)

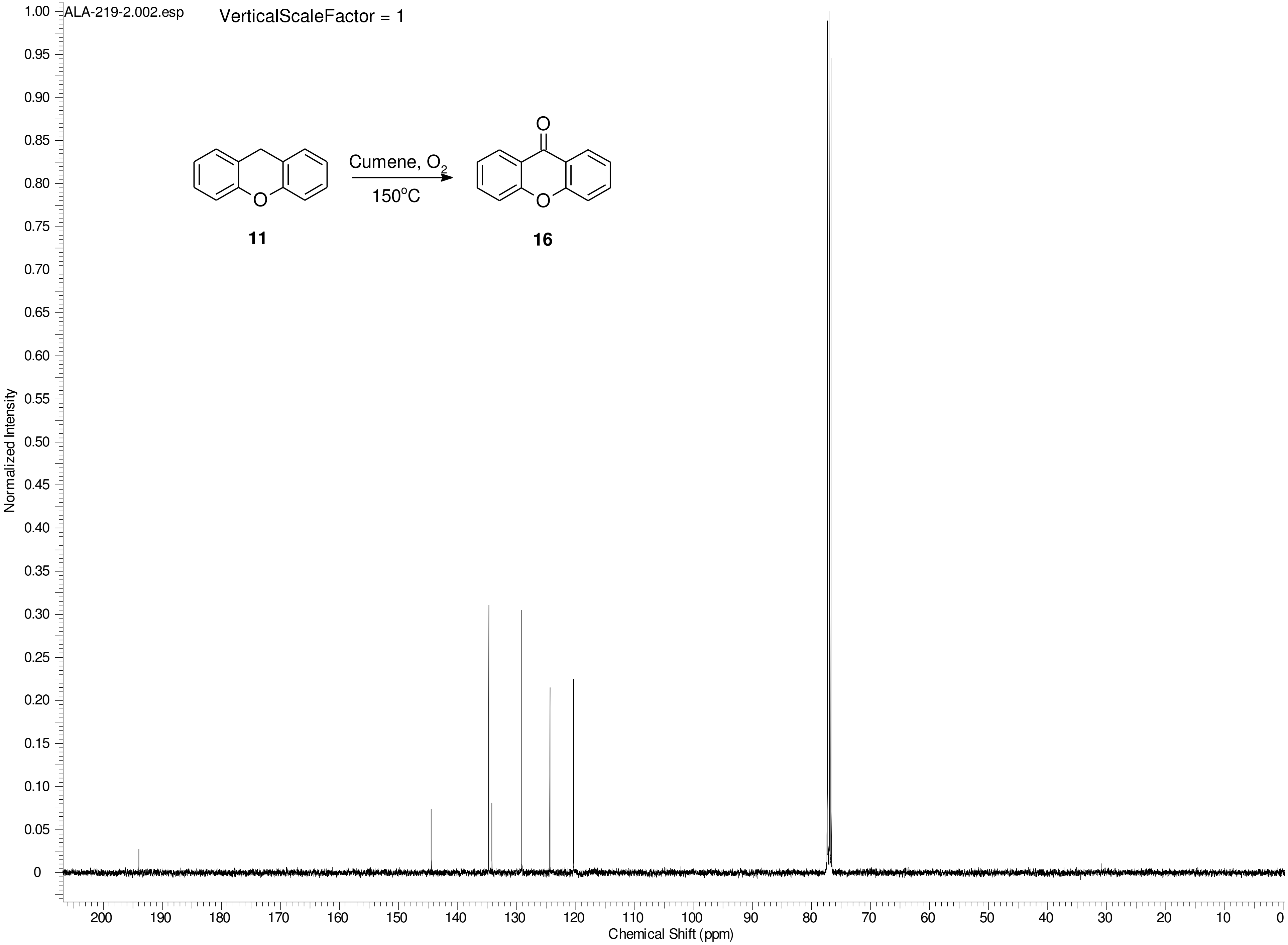
ALA-219-2.002.esp
VerticalScaleFactor = 1
Cumene, O_2
150°C
11
16
Normalized Intensity
Chemical Shift (ppm)

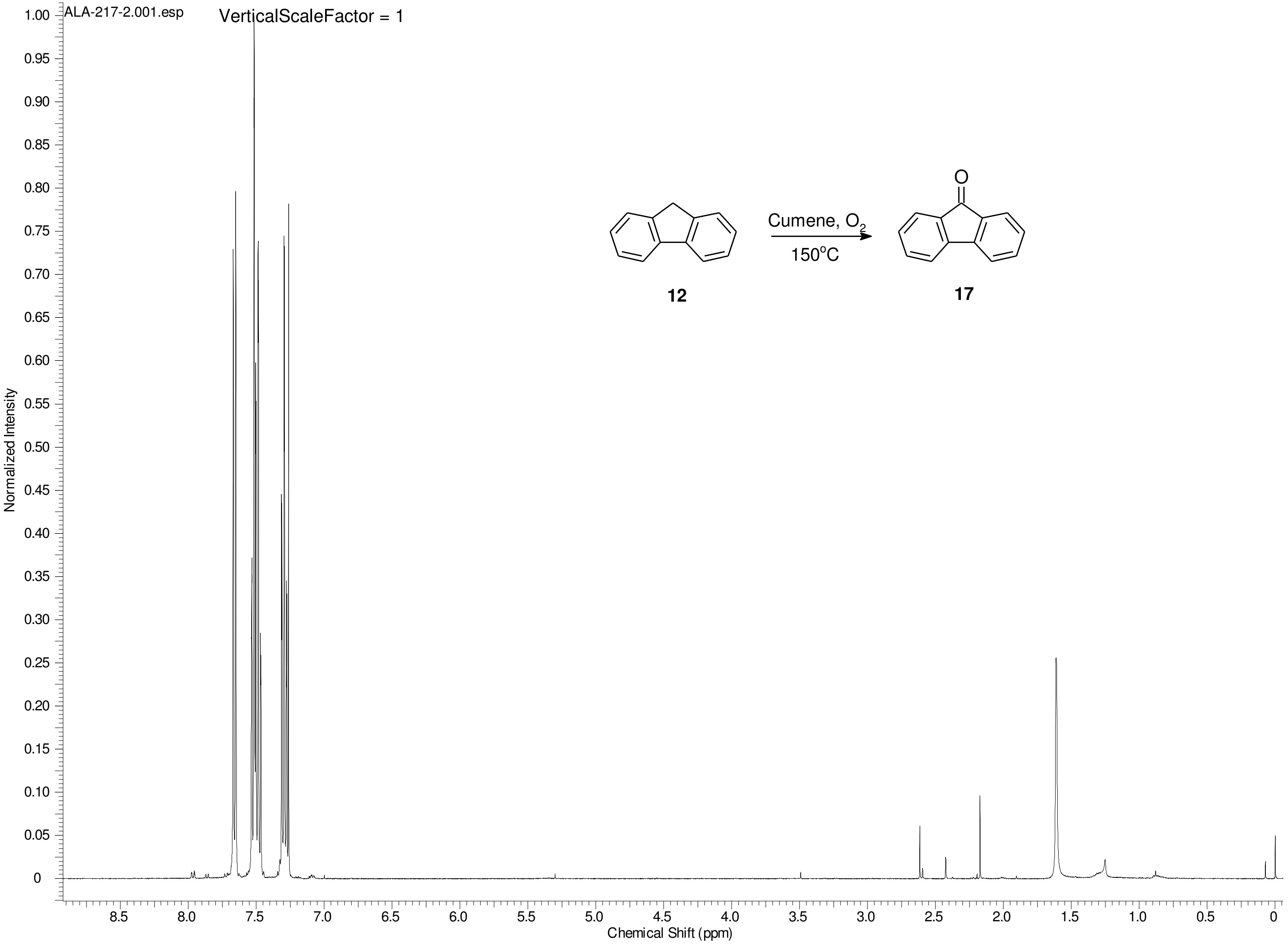

ALA-217-2.001.esp
VerticalScaleFactor = 1
Cumene, O_2
150°C
12
17
Normalized Intensity
Chemical Shift (ppm)

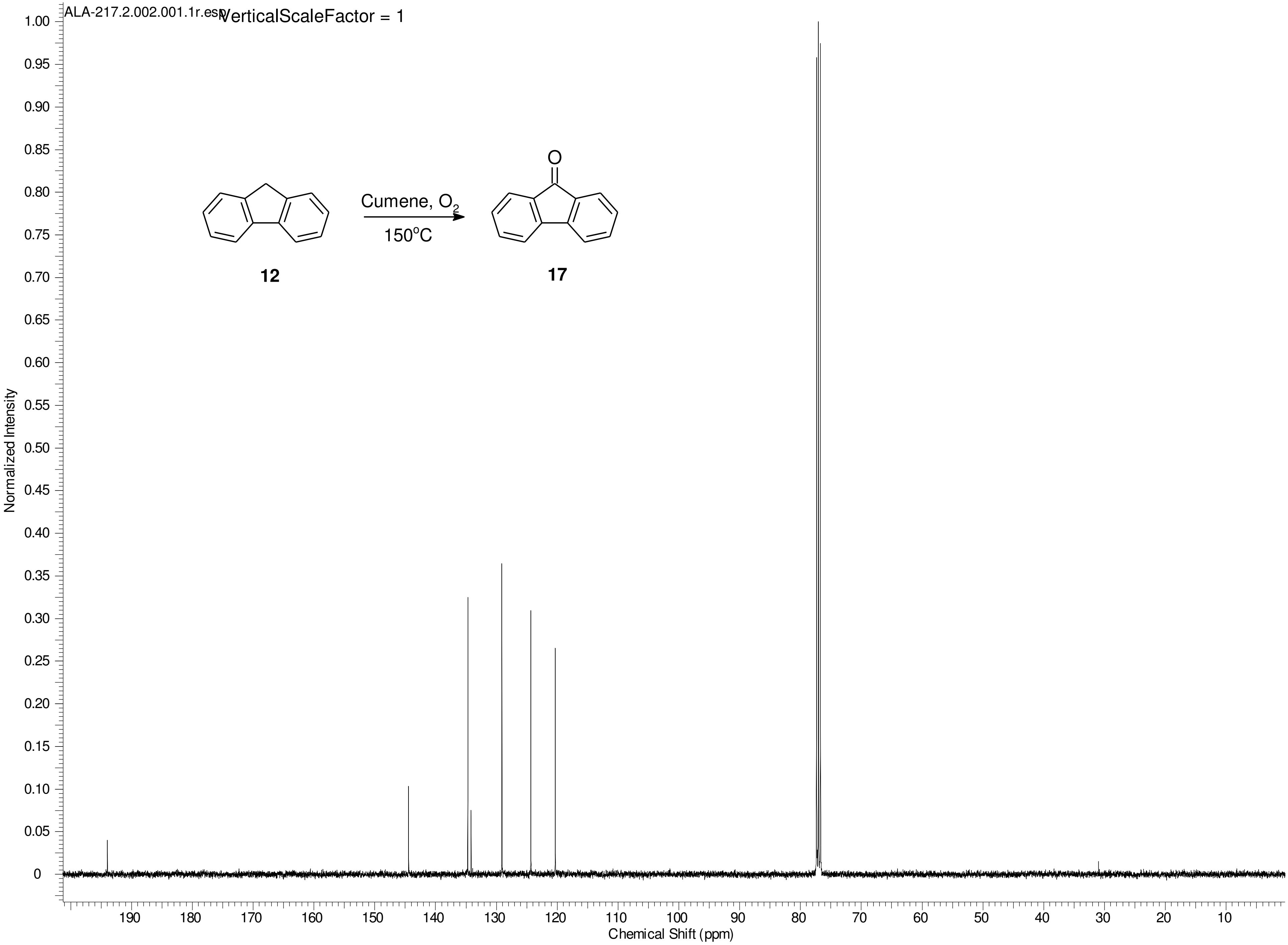
ALA-217.2.002.001.1r.esp
VerticalScaleFactor = 1
Cumene, O_2
150°C
12
17
O
Normalized Intensity
Chemical Shift (ppm)
1.00
0.95
0.90
0.85
0.80
0.75
0.70
0.65
0.60
0.55
0.50
0.45
0.40
0.35
0.30
0.25
0.20
0.15
0.10
0.05
0
190
180
170
160
150
140
130
120
110
100
90
80
70
60
50
40
30
20
10

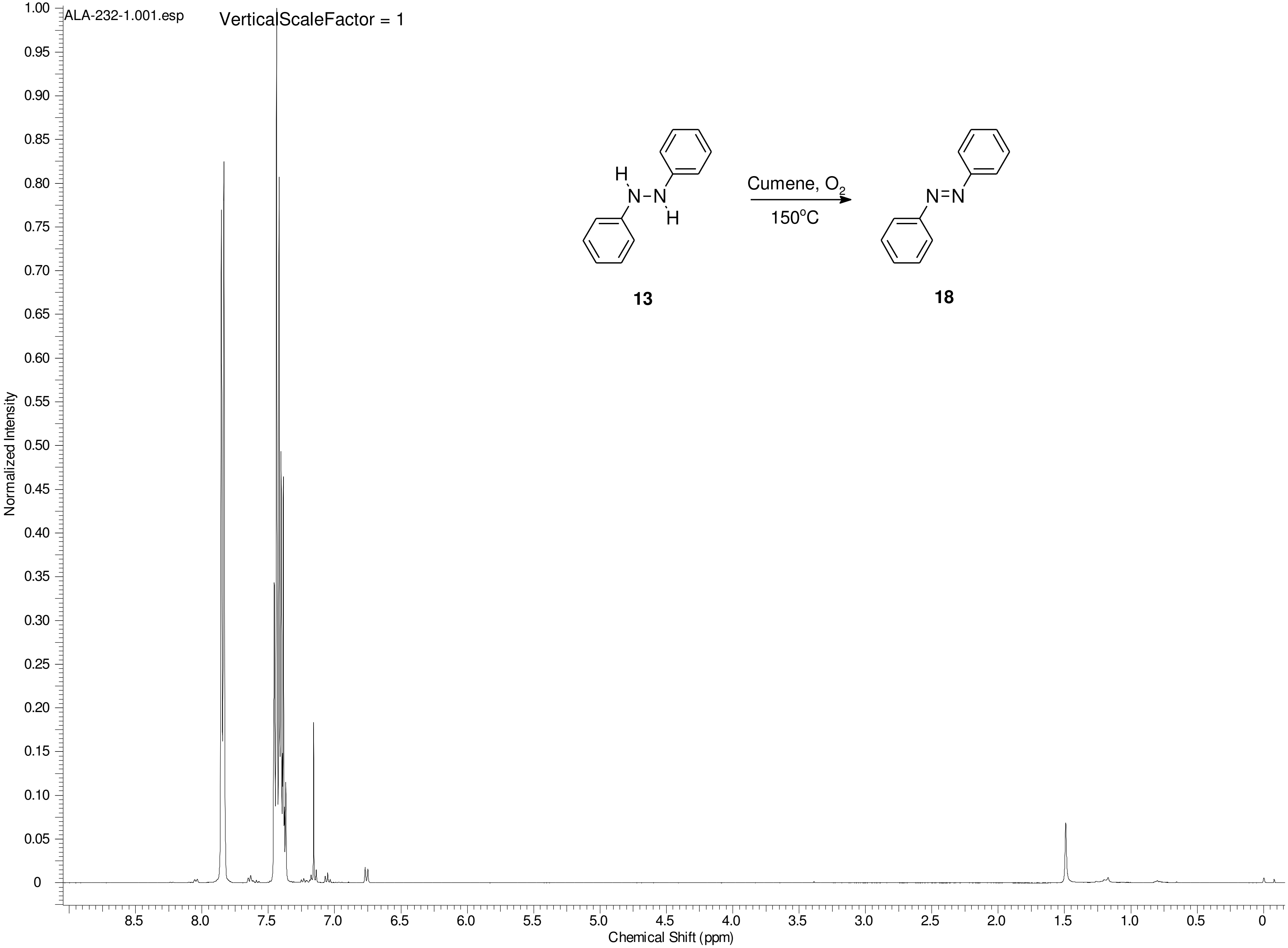
ALA-232-1.001.esp
VerticalScaleFactor = 1
Normalized Intensity
Chemical Shift (ppm)
Cumene, O_2
150°C
13
18

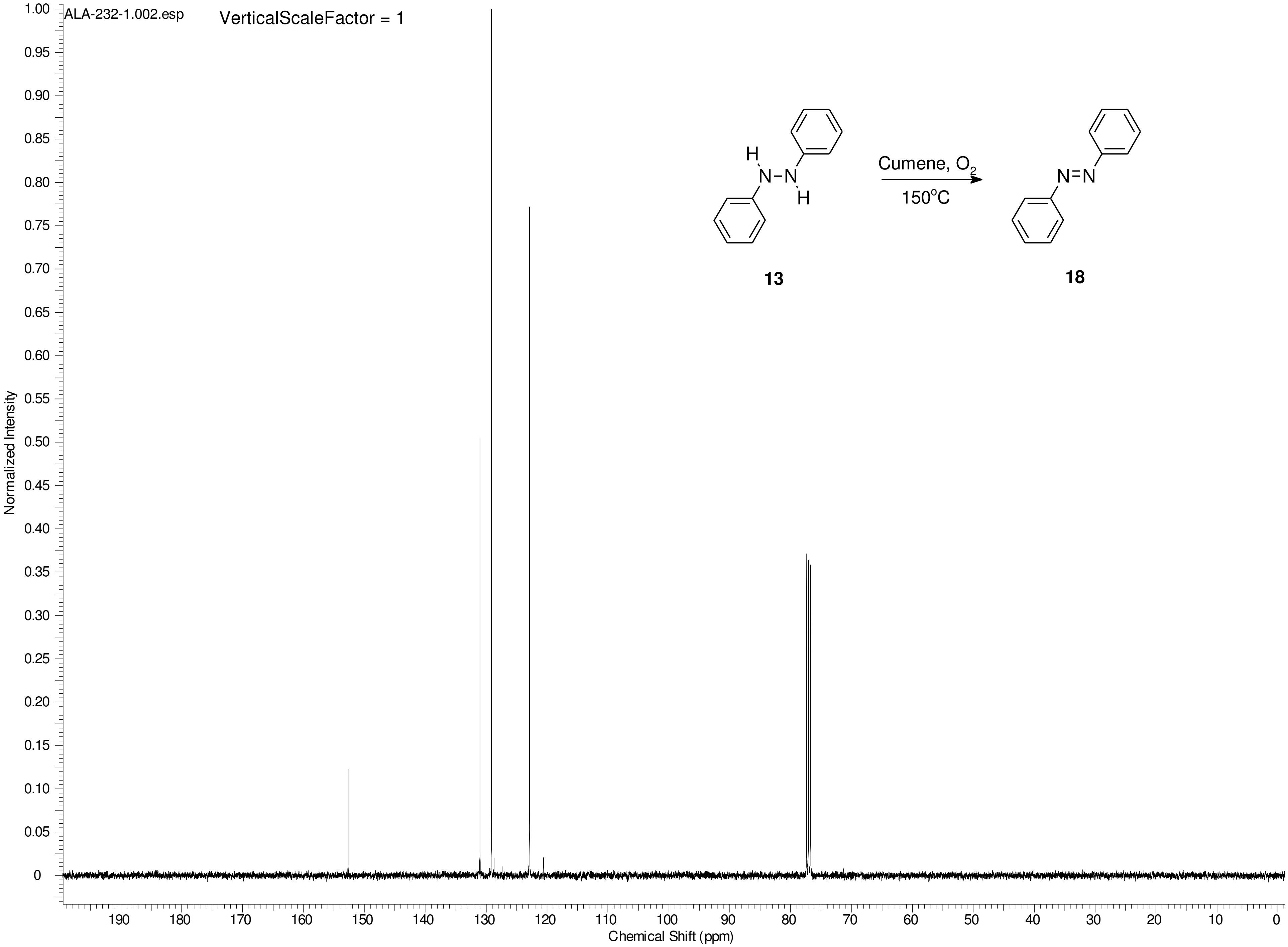

ALA-232-1.002.esp
VerticalScaleFactor = 1
Cumene, O2
150°C
13
18
Normalized Intensity
Chemical Shift (ppm)
